# Antibiotic-resistant characteristics and horizontal gene transfer ability analysis of extended-spectrum β-lactamase-producing *Escherichia coli* isolated from giant pandas

**DOI:** 10.3389/fvets.2024.1394814

**Published:** 2024-07-26

**Authors:** Haifeng Liu, Siping Fan, Xiaoli Zhang, Yu Yuan, Wenhao Zhong, Liqin Wang, Chengdong Wang, Ziyao Zhou, Shaqiu Zhang, Yi Geng, Guangneng Peng, Ya Wang, Kun Zhang, Qigui Yan, Yan Luo, Keyun Shi, Zhijun Zhong

**Affiliations:** ^1^College of Veterinary Medicine, Sichuan Agricultural University, Key Laboratory of Animal Disease and Human Health of Sichuan, Chengdu, China; ^2^Jiangsu Yixing People’s Hospital, Yixing, China; ^3^The Chengdu Zoo, Institute of Wild Animals, Chengdu, China; ^4^China Conservation and Research Centre for the Giant Panda, Key Laboratory of SFGA on the Giant-Panda, Ya'an, Sichuan, China

**Keywords:** *Escherichia coli*, extended-spectrum β-lactamase, antibiotic resistance gene, virulence-associated gen, horizontal gene transfer, giant panda

## Abstract

Extended-spectrum β-lactamase (ESBL)-producing *Escherichia coli* (ESBL-EC) is regarded as one of the most important priority pathogens within the One Health interface. However, few studies have investigated the occurrence of ESBL-EC in giant pandas, along with their antibiotic-resistant characteristics and horizontal gene transfer abilities. In this study, we successfully identified 12 ESBL-EC strains (8.33%, 12/144) out of 144 *E. coli* strains which isolated from giant pandas. We further detected antibiotic resistance genes (ARGs), virulence-associated genes (VAGs) and mobile genetic elements (MGEs) among the 12 ESBL-EC strains, and the results showed that 13 ARGs and 11 VAGs were detected, of which *bla*_CTX-M_ (100.00%, 12/12, with 5 variants observed) and *papA* (83.33%, 10/12) were the most prevalent, respectively. And *ISEcp1* (66.67%, 8/12) and *IS26* (66.67%, 8/12) were the predominant MGEs. Furthermore, horizontal gene transfer ability analysis of the 12 ESBL-EC showed that all *bla*_CTX-M_ genes could be transferred by conjugative plasmids, indicating high horizontal gene transfer ability. In addition, ARGs of *rmtB* and *sul2*, VAGs of *papA*, *fimC* and *ompT*, MGEs of *ISEcp1* and *IS26* were all found to be co-transferred with *bla*_CTX-M_. Phylogenetic analysis clustered these ESBL-EC strains into group B2 (75.00%, 9/12), D (16.67%, 2/12), and B1 (8.33%, 1/12), and 10 sequence types (STs) were identified among 12 ESBL-EC (including ST48, ST127, ST206, ST354, ST648, ST1706, and four new STs). Our present study showed that ESBL-EC strains from captive giant pandas are reservoirs of ARGs, VAGs and MGEs that can co-transfer with *bla*_CTX-M_ via plasmids. Transmissible ESBL-EC strains with high diversity of resistance and virulence elements are a potential threat to humans, animals and surrounding environment.

## 1 Introduction

Extended-spectrum β-lactamase (ESBL)-producing *Escherichia coli* (ESBL-EC), which is resistant to many β-lactamase antibiotics, is one of the top priority pathogens within the One Health interface, classified by the World Health Organization (WHO) (1, 2). In recent years, there has been a significant increase in the prevalence of ESBL-EC in animals, particularly in wildlife population (1, 3–6). This has sparked concerns among researchers and experts in the field of animal health, as the emergence of ESBL-producing bacteria has limited the treatment options available for bacterial infections in animals (7). The predominant ESBL genes include *bla*_TEM_, *bla*_SHV_, and *bla*_CTX-M_, of which *bla*_CTX-M_ is the most prevalent type in Enterobacteriaceae, especially in *E. coli* (7). These ESBL genes can be transferred between different bacteria via mobile genetic elements (MGEs), such as plasmids carrying antibiotic resistance genes (ARGs) and virulence-associated genes (VAGs), accelerating the occurrence of clinical ESBL-producing pathogen (8, 9).

The giant panda (*Ailuropoda melanoleuca*) is the national symbol of China and a popular attraction for tourists visiting zoos in China and other countries (10, 11). In addition, the wild release plan of captive giant pandas implemented by the Chinese government has raised concerns about the spread of antimicrobial resistance (AMR) bacteria to other wildlife and the natural environment, or the potential transmission of AMR bacteria from other wildlife to giant pandas (12, 13). However, there have been few of publications on the presence of ESBL-EC in giant pandas in recent years (14). Qin et al. (9) analyzed 96 *E. coli* strains isolated from healthy captive giant pandas from 2012 to 2013 and found that 25 of those were ESBL-EC, and three types of ESBL genes (*bla*_TEM_, *bla*_CTX-M_, and *bla*_OXA_) were detected. Another study of diseased captive giant pandas detected four ESBL-EC one atypical enteropathogenic *E. coli* isolated in 2015, and three extraintestinal pathogenic *E. coli* isolated in 2008 and 2012, and these four ESBL-EC were resistant to more than eight antibiotics, and two variants of *bla*_CTX-M_ (*bla*_CTX-M-55_ and *bla*_CTX-M-105_) were detected (14). The above studies indicate that ESBL-EC from healthy or diseased captive giant pandas carrying ESBL genes exhibit serious AMR and the occurrence of ESBL-EC poses a significant challenge to antibiotic treatment for giant pandas.

Our previous study showed the presence of antibiotic-resistant *E. coli* in clinically healthy captive giant pandas and demonstrated that *E. coli* strains were a pool of ARGs, VAGs, and MGEs (15). However, the characteristics of ESBL-EC from those captive giant pandas, especially regarding AMR characteristics including ARGs, VAGs, MGEs, phylogenetic groups, MLST, and the ability for horizontal gene transfer (HGT), remain unknown and need to be clarified. This study provides a deeper understanding of the AMR profile of ESBL-EC strains from captive giant pandas, offering insights into their potential impact on public health and environmental ecosystems.

## 2 Materials and methods

### 2.1 Isolation and identification of *E. coli*

From 2020 to 2021, 117 fresh fecal samples from different individuals were collected from captive giant pandas living at the Chengdu Research Base of Giant Panda Breeding (CRBGP). From 2018 to 2021, 27 fecal samples were collected from wild giant pandas living in the Sichuan Wolong National Nature Reserve. All giant pandas involved in this study were in a healthy state and did not exhibit any abnormal symptoms, as confirmed by a professional veterinarian. Isolation and identification of *E. coli* were performed as previously described (16–18). Briefly, fecal samples were immediately placed in sterile disposable sampling tubes, stored in a cooler at 2°C ~ 8°C, and transported to Sichuan Agricultural University for isolation and identification within 24 h. Samples were enriched in LB broth, and all the isolates were confirmed by Gram staining, MacConkey agar (Solarbio, Beijing), eosin methylene blue agar (Chromagar, France), and biochemical identification by API 20E system (BioMerieux, France) (18). The 16 S rRNA of all strains was further amplified to confirm the isolate as *E. coli* (16). These strains were stored in Luria-Bertani (LB) broth containing 50% glycerol at −20°C for further analysis.

### 2.2 Screening of ESBL-EC isolates

Phenotypic screening was measured using the double-disc diffusion test as recommended by the Clinical and Laboratory Standards Institute (CLSI, 2023). Clinical and Laboratory Standards Institute (2023). Performance standards for antimicrobial susceptibility testing, M100-33Ed, PA: Clinical and Laboratory Standards Institute. Clinical and Laboratory Standards Institute (2023). Performance standards for antimicrobial susceptibility testing, M100-33Ed, PA: Clinical and Laboratory Standards Institute. Briefly, antibiotic discs (Oxoid, Basingstoke, United Kingdom) of cefotaxime (CTX, 30 μg), cefotaxime plus clavulanic acid (CTL, 30/10 μg), ceftazidime (CAZ, 30 μg) and ceftazidime plus clavulanic acid (CAL, 30/10 μg) were used to screen for ESBL-EC isolates. When the diameter of the inhibition zone increased by ≥5 mm with clavulanic acid, compared with that without clavulanic acid, the isolate is considered as ESBL-EC.

### 2.3 Antimicrobial susceptibility testing

All ESBL-EC isolates were tested using the standard disk diffusion method recommended by the CLSI for susceptibility to fifteen antimicrobials. We used antimicrobial disks (Oxoid, Basingstoke, United Kingdom) in six categories: β-lactams (aztreonam, AZM, 30 μg; ampicillin, AMP, 10 μg; amoxicillin/clavulanic acid 2:1, AMC, 20/10 μg; ampicillin/sulbactam 1:1, SAM, 10/10 μg; cefazolin, CEZ, 20 μg; cefotaxime, CTX, 30 μg; ceftriaxone, CRO, 30 μg; ceftazidime, CAZ, 30 μg), aminoglycosides (gentamicin, GM, 10 μg; amikacin, AMK, 30 μg), quinolones (ciprofloxacin, CIP, 5 μg), tetracyclines (tetracycline, TET, 30 μg; doxycycline, DOX, 30 μg), sulfonamides (trimethoprim-sulfamethoxazole, SXT, 23.75/1.25 μg) and amide alcohols (chloramphenicol, CHL, 30 μg). Results were interpreted in according to CLSI 2023 criteria. *E. coli* ATCC25922 was used as a control. Fifteen antimicrobials were selected for the experiment: AZM, AMC, SAM, CEZ, CTX and CRO were used in the Chengdu Research Base of Giant Panda Breeding, and AMP, CAZ, GM, AMK, CIP, TET, DOX, SXT and CHL were found to be resistant to *E. coli* in giant pandas in our previous study (18). The multidrug resistant (MDR) strain was defined as being resistant to at least three antimicrobial categories (19).

### 2.4 Screening of ARGs, VAGs and MGEs from ESBL-EC isolates

Total genomic DNA was extracted from ESBL-EC isolates using the TaKaRa Bacteria DNA Kit (Takara Biomedical Technology Biotech, Beijing, China) according to the manufacturer’s instructions. DNA quality was checked by ultraviolet-absorbance (ND1000, Nanodrop, Thermo Fisher Scientific). DNA samples were stored at −20°C for subsequent polymerase chain reaction (PCR) detection.

We screened for 15 ARGs (including ESBL genes: *bla*_TEM_, *bla*_SHV_, *bla*_CTX-M_), 20 VAGs (5 categories) and 16 MGEs by PCR. Primers were synthesized by Huada Gene Technology Co., Ltd. (Shenzhen, China). Primers and the amplification conditions are shown in Supplementary Table S1. PCR products were separated by gel electrophoresis in a 1.0% agarose gel stained with GoldViewTM (Sangon Biotech, Shanghai, China) and photographed under ultraviolet light using a Bio-Rad ChemiDoc MP omnipotent imager (Bole, United States). All positive PCR products were sequenced with Sanger sequencing in both directions by Sangon Biotech (Shanghai, China). Sequences were analyzed online using the BLAST function of NCBI.^1^

### 2.5 Conjugation experiment and PCR-based replicon typing (PBRT)

To determine the transfer ability of resistance genes, all ESBL-EC isolates were selected as donors for conjugation. The azide-resistant *E. coli* J53 was used as recipient bacteria. Donor and recipient strains were grown separately overnight in 4 mL of LΒ-Broth. Volumes of 0.2 mL of donor and 0.8 mL of recipient strains were added to 4 mL of LB broth and cultured overnight. Transconjugants were selected on Azide dextrose agar plates (150 mg/mL; Qingdao Hope Bio-Technology Co., Ltd. Qingdao, China) supplemented with cefotaxime (4 mg/L; Shanghai Yuanye Bio-Technology Co., Ltd. Shanghai, China). Transfer frequencies were calculated per recipient cell. HGT frequency was calculated by dividing the number of transconjugants by the number of recipient. All transconjugants were confirmed by PCR for genes encoding ESBL production and tested for susceptibility to the same antibiotic used against the donor isolates. And the same ARGs, VAGs, MGEs carried by donor isolates were detected by PCR for all transconjugants.

The plasmid replicon types of ESBL-producing bacteria (donor) and their transconjugants were determined as previously described (20). Briefly, amplification by PCR was performed with 18 pairs of primers recognizing HI1, HI2, I1, X, L/M, N, FIA, FIB, W, Y, P, FIC, A/C, T, FIIA, FrepB, K, and B/O in 5 multiplex and 3 simplex reactions. The PCR products were analyzed as described in 2.4. The primers and the amplification conditions are shown in Supplementary Table S1.

### 2.6 Phylogenetic grouping and MLST typing of ESBL-EC isolates

Phylogenetic grouping for 12 ESBL-EC is categorized into four major phylogenetic classes (A, B1, B2 and D) using triplex PCR targeting three genes (*ChuA*, *yjaA* and *TSPE4.C2*) according to Clermont et al. (21). For multilocus sequence typing (MLST), PCR protocols were performed as previously described (22). All the primers and the amplification conditions are shown in Supplementary Table S1. All positive PCR products were sequenced with Sanger sequencing in direction by Sangon Biotech (Shanghai, China). Sequences of housekeeping gene for MLST were analyzed online using the pubMLST database.^2^

The goeBURST algorithm in phyloviz 2.0 was used for clustering analysis of STs for 12 ESBL-EC isolates, which divided the STs into several clusters consist of closely related STs with two allelic differences (23). A clonal complex is typically composed of a single predominant genotype and closely related genotype (24).

## 3 Results

### 3.1 Identification and antimicrobial susceptibility of ESBL-EC

A total of 144 *E. coli* isolates (one isolate per fecal sample) were obtained from 117 captive and 27 wild giant pandas, respectively. Twelve ESBL-EC isolates (10.26%, 12/117) were identified from captive giant pandas, while no ESBL-EC isolate was detected from wild giant pandas.

The antimicrobial susceptibility testing results of 12 ESBL-EC to 15 antibiotics in 6 categories were shown in Table 1. Top 5 resistance rates to 15 antimicrobial agents were AMP (100.00%, 12/12), CEZ (100.00%, 12/12), CTX (100.00%, 12/12), CRO (100.00%, 12/12), and TET (50.00%, 6/12). The resistance rate to CIP (8.33%, 1/12) was the lowest, and the remaining 9 antibiotics ranged from 41.67% (AZM, DOX) to 16.67% (SAM, CAZ) (Table 1). For the 6 antibiotic categories, the resistance rate to β-lactam antibiotics was the highest (100%, 12/12), followed by tetracyclines (50%, 6/12), aminoglycosides (33.33%, 4/12), sulfonamides (33.33%, 4/12) and amide alcohols (33.33%, 4/12). The resistance rate to quinolones antibiotics was the lowest (8.33%, 1/12). Phenotypic characterization of antibiotic resistance indicated that 50% ESBL-EC isolates (GP003, GP004, GP0012, GP022, GP030 and GP065) were classified as MDR strains, of which strain GP003 was resistant to 12 antibiotics.

**Table 1:** Resistance pattern of 12 ESBL-producing *E. coli* isolates from captive giant pandas.

Strains	Age	Gender	Resistance profiles	Antibiotic categories	MDR
GP001	36	Female	AMP/CEZ/CTX/CRO/GM/AMK	β-lactams/Aminoglycosides	-
GP003	33	Male	AZM/AMP/CEZ/CTX/CRO/CAZ/GM/AMK/TET/ DOX/SXT/CHL	β-lactams/Aminoglycosides/Tetracyclines/Sulfonamides/Amide alcohols	MDR
GP004	30	Female	AMP/AMC/CEZ/CTX/CRO/GM/AMK/TET/CHL	β-lactams/Aminoglycosides/Tetracyclines/Amide alcohols	MDR
GP012	20	Female	AMP/CEZ/CTX/CRO/TET/DOX/CHL	β-lactams/ Tetracyclines/Amide alcohols	MDR
GP014	19	Male	AMP/SAM/CEZ/CTX/CRO	β-lactams	-
GP022	15	Female	AZM/AMP/AMC/CEZ/CTX/CRO/CIP/TET/ DOX/SXT	β-lactams/Quinolones/ Tetracyclines/Sulfonamides	MDR
GP030	13	Female	AMP/AMC/SAM/CEZ/CTX/CRO/TET/DOX/SXT	β-lactams/ Tetracyclines/Sulfonamides	MDR
GP032	13	Female	AMP/AMC/CEZ/CTX/CRO	β-lactams	-
GP050	10	Female	AZM/AMP/CEZ/CTX/CRO	β-lactams	-
GP065	7	Female	AMP/CEZ/CTX/CRO/GM/AMK/TET/ DOX/SXT/CHL	β-lactams/Aminoglycosides/Tetracyclines/Sulfonamides/ Amide alcohols	MDR
GP095	3	Male	AZM/AMP/CEZ/CTX/CRO	β-lactams	-
GP101	3	Female	AZM/AMP/CEZ/CTX/CRO/CAZ	β-lactams	-

MDR stands for Multi-Drug Resistance in the context of bacteria, indicating resistance to 3 or more antibiotic classes.

### 3.2 Distribution of ARGs, VAGs and MGEs in ESBL-EC isolates

The detection rates of ARGs, VAGs and MGEs for 12 ESBL-EC isolates are shown in Table 2.We detected 13 out of 15 currently known ARGs, including β-lactamase: *bla*_CTX-M_ (100.00%, 12/12), *bla*_TEM_ (66.67%, 8/12), *bla*_SHV_ (8.33%, 1/12), tetracyclines: *tetA* (58.33%, 7/12), *tetC* (8.33%, 1/12), sulfonamides: *sul1* (58.33%, 7/12), *sul3* (33.33%, 4/12), *sul2* (8.33%, 1/12); quinolones: *qnrS* (50.00%, 6/12), *oqxAB* (8.33%, 1/12), amide alcohols: *cmlA* (50.00%, 6/12), *flor* (41.66%, 5/12), and aminoglycosides: *rmtB* (33.33%, 4/12). The *armA* of aminoglycoside ARGs and *qnrA* of quinolone ARGs were not detected. Furthermore, all ESBL-encoding genes were sequenced to identify the variants, totally 1 variant of *bla*_SHV_, 3 variants of *bla*_TEM_, and 5 variants of *bla*_CTX-M_ were detected. The most prominent *bla*_CTX-M_ variant observed was *bla*_CTX-M-55_ (33.33%, 4/12), followed by *bla*_CTX-M-13_ (25.00%, 3/12), *bla*_CTX-M-27_ (25.00%, 3/12), *bla*_CTX-M-14_ (8.33%, 1/12), and *bla*_CTX-M-15_ (8.33%, 1/12). For *bla*_TEM_, the most prominent variant was *bla*_TEM-1_ (50.00%, 6/12), followed by *bla*_TEM-135_ (8.33%, 1/12) and *bla*_TEM-176_ (8.33%, 1/12). For *bla*_SHV_, only *bla*_SHV-1_ (8.33%, 1/12) was detected.

**Table 2:** Distribution of ARGs, VAGs and MGEs in 12 ESBL-producing *E. coli* isolates from captive giant pandas.

Strains	ARGs	VAGs	MGEs
GP001	*bla*_CTX-M-14_ + *bla*_TEM-1_ + *rmtB*	*papA* + *fimC* + *astA* + *vat* + *fyuA* + *iroN* + *irp2* + *sitA* + *ompT* + *iss*	*trbC* + *ISEcp1* + *IS26* + *intI1*
GP003	*bla*_CTX-M-55_ + *bla*_TEM-1_ *+ rmtB* + *qnrS* + *tetA* + *sul3* + *cmlA* + *flor*	*papA* + *fimC* + *astA* + *iroN*	*ISEcp1* + *IS26*
GP004	*bla*_CTX-M-55_ + *bla*_TEM-1_ + *rmtB* + *qnrS* + *tetA* + *cmlA* + *flor*	*papA* + *astA* + *iroN* + *ompT*	*trbC* + *IS26* + *intI1*
GP012	*bla*_CTX-M-27_ + *bla*_TEM-135_ + *bla*_SHV-1_ + *qnrS* + *oqxAB* + *tetA* + *tetC* + *sul1* + *flor*	*eaeA* + *fimC* + *vat* + *fyuA* + *irp2* + *ompT* + *iss*	*trbC* + *tnpA/Tn21* + *IS1133* + *intI1*
GP014	*bla*_CTX-M-13_ + *sul1*	*papA* + *astA* + *fyuA*	*ISEcp1*
GP022	*bla*_CTX-M-15_ + *bla*_TEM-176_ + *qnrS* + *tetA* + *sul1* + *sul3* + *cmlA* + *flor*	*papA* + *fimC* + *fyuA* + *iroN* + *irp2* + *sitA* + *ompT*	*trbC* + *tnpA/Tn21* + *ISCR3/14* + *IS1133* + *ISEcp1* + *IS26*
GP030	*bla*_CTX-M-27_ + *qnrS* + *tetA* + *sul1* + *sul2* + *cmlA*	*papA* + *fimC* + *astA* + *vat* + *fyuA* + *iroN* + *sitA* + *ompT* + *iss*	*trbC* + *tnpA/Tn21* + *merA* + *IS26* + *intI1*
GP032	*bla*_CTX-M-27_ + *bla*_TEM-1_ + *tetA* + *sul3* + *cmlA*	*fimC*	*merA* + *IS26*
GP050	*bla*_CTX-M-13_ + *sul1*	*papA* + *fyuA* + *irp2* + *sitA*	*ISEcp1*
GP065	*bla*_CTX-M-55_ + *bla*_TEM-1_ + *rmtB* + *qnrS* + *tetA* + *sul3* + *cmlA* + *flor*	*papA* + *fimC* + *astA* + *iroN* + *ompT*	*trbC* + *ISEcp1* + *IS26* + *intI1*
GP095	*bla*_CTX-M-13_ + *sul1*	*papA* + *fimC* + *fyuA* + *irp2* + *sitA*	*trbC* + *ISEcp1*
GP101	*bla*_CTX-M-55_ + *bla*_TEM-1_ + *sul1*	*papA* + *astA* + *fyuA* + *irp2*	*ISEcp1* + *IS26*

A total of 11 VAGs in 4 categories out of 20 currently known VAGs in 5 categories were detected among 12 ESBL-EC isolates, with a maximum of 10 VAGs detected in strain GP001. The VAGs detected included adhesion-related genes: *papA* (83.33%, 10/12), *fimC* (66.67%. 8/12), *eaeA* (8.33%, 1/12); iron transport-related genes: *fyuA* (66.67%, 8/12), *iroN* (50.00%, 6/12), *Irp2* (50.00%, 6/12), *sitA* (41.67%, 5/12); invasion-and toxin-related genes: *astA* (58.33%, 7/12), *vat* (25.00%, 3/12), and antiserum survival factor: *ompT* (50.00%, 6/12), *iss* (25.00%, 3/12). The remaining 9 VAGs were not detected.

Eight out of 16 currently known MGEs were detected in 12 ESBL-EC isolates, including *ISEcp1* (66.67%, 8/12), *IS26* (66.67%, 8/12), *trbC* (58.33%, 7/12), *intI1* (41.67%, 5/12), *tnpA/Tn21* (25.00%, 3/12), *merA* (16.67%, 2/12), *IS1133* (16.67%, 2/12), and *ISCR3/14* (8.33%, 1/12). The other 8 MGEs were not detected. We further analyzed the integron gene cassettes of isolates that carried *intI1*, and no gene cassettes detected.

### 3.3 Conjugative transfer of plasmids with different replicon types

**Figure 1:**
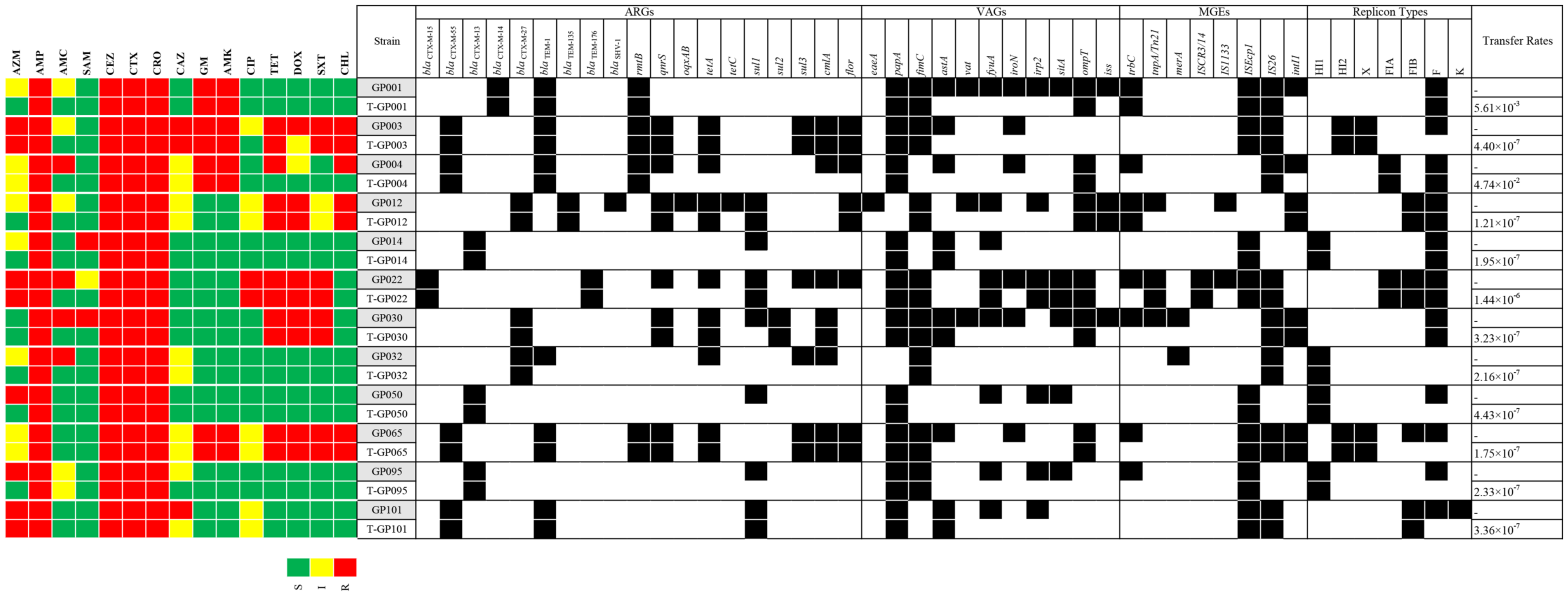
A heat-map showing the comparison of the twelve *E. coli* donors and the resultant transconjugants for antimicrobial resistance profile, ARGs, VAGs, MGEs, plasmid replicon types, and conjugative transfer rates. The “T” in front of the strain name represents the transconjugant. Black squares indicate the identified ARGs, VAGs, MGEs, and replicon types. S, susceptible; I, intermediate susceptible; R, resistant. Only positive ARGs, VAGs, MGEs, and replicon types are shown.

We further investigated the transfer ability of resistance genes. All the ESBL-EC isolates transferred their cefotaxime resistance determinant to the azide resistant *E. coli* J53 recipient, with transfer frequencies ranging from 1.21 × 10^−7^ (strain GP012) to 4.74 × 10^−2^ (strain GP004) (Figure 1). The 12 transconjugants were confirmed to possessed ESBL-producing phenotype and carried *bla*_CTX − M_ gene. In addition, resistance to aminoglycosides, quinolones, tetracyclines, sulfonamides, amide alcohols and other β-lactams were also co-transferred to the recipient along with cefotaxime resistance. The detail of transfer frequencies of ARGs, VAGs and MGEs was showed in Figure 1. Among them, the conjugation transfer frequencies of ARGs of *bla*_CTX − M_, *rmtB* and *sul2*, VAGs of *papA*, *fimC* and *ompT*, MGEs of *ISEcp1*, *IS26* and *ISCR3/14* were 100.00%. However, ARGs of *bla*_SHV_, *tetC* and *oqxAB*, VAGs of *iroN*, *vat* and *eaeA*, MGEs of *merA* and *IS1133* were not detected in the transconjugants, indicating that no horizontal transfer of these genes occurred. PCR-based replicon typing (PBRT) showed that the 12 ESBL-EC isolates contained plasmids with different replicons, including IncFrepB (91.67%, 11/12), IncHI1 (33.33%, 4/12), IncFIB (33.33%, 4/12), IncHI2 (16.67%, 2/12), IncX (16.67%, 2/12), IncFIA (16.67%, 2/12) and IncK (8.33%, 1/12). Moreover, PBRT of the transconjugants confirmed that among the 7 replicons, 6 conjugative plasmid types (IncFrepB, IncHI1, IncFIB, In-cHI2, IncX and IncFIA) can transfer the ESBL genes, only IncK was not conjugated.

### 3.4 Phylogenetic grouping and MLST characteristics

**Figure 2:**
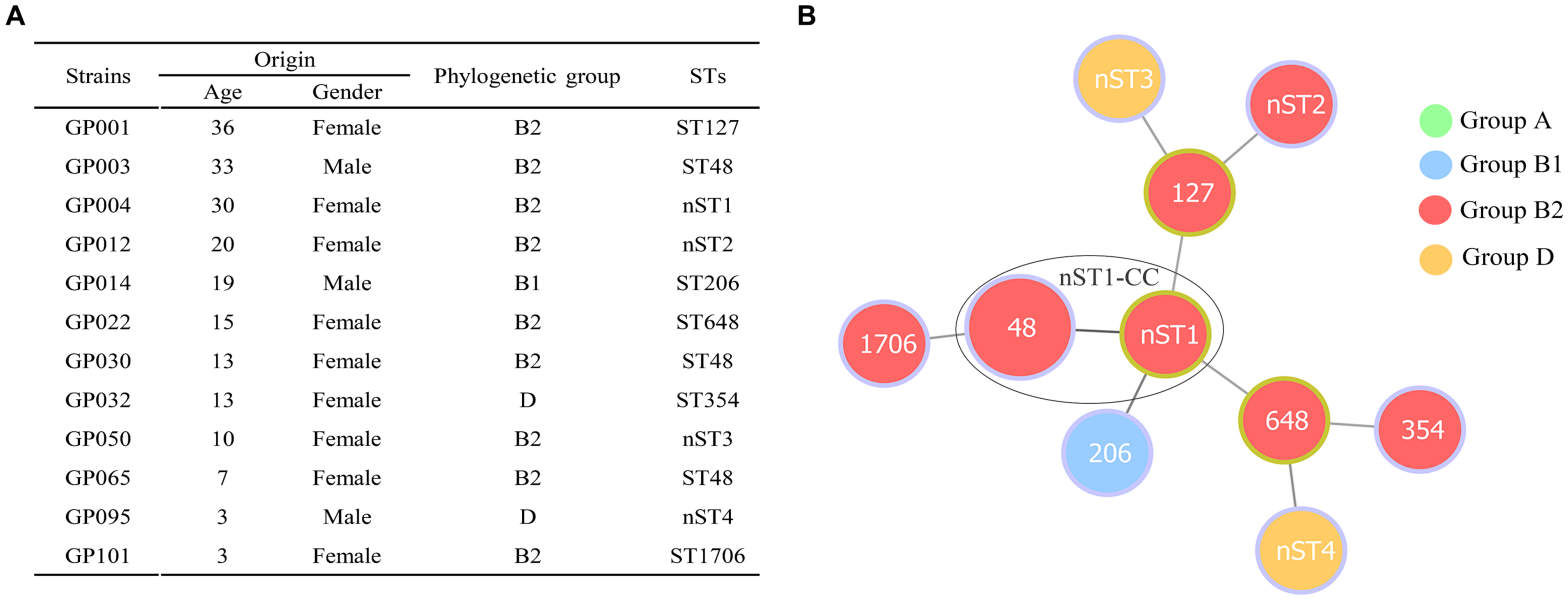
Distribution of phylogenetic groups and STs in 12 ESBL-producing *E. coli* isolates from giant pandas. **(A)** The detailed information of phylogenetic groups and STs in 12 ESBLs-EC isolates from giant pandas. **(B)** Minimum spanning tree of MLST types in 12 ESBLs-EC strains. The size of circle indicates the proportion of isolates belonging to the ST. The color within each circle represents phylogroups and indicates the proportion of isolates belonging to different phylogroups. Each link between circles indicates a mutational event and the distance is scaled as the number of allele differences between STs. The yellow-green outlines of the circles represent the founder ST of a clonal complex (CC), and the other STs (with purple outlines of the circles) are derived from the founder ST with two allelic differences. A high diversity of STs (10 STs were identified) was observed in 12 ESBLs-EC strains, ST48 being the most prevalent lineage. Only one clonal complex (nST1-CC, containing ST48 and nST1) was observed in the present study.

Phylogenetic screening of 12 ESBL-EC isolates confirmed that group B2 (75.00%, 9/12) was the most commonly observed phenotype, followed by group D (16.67%, 2/12) and group B1 (8.33%, 1/12) (Figure 2A). Group A was not detected in our study. MLST analysis showed that 10 different STs (containing 6 known STs and 4 new STs) were observed in 12 ESBL-EC isolates, of which ST48 was the most frequent (25%, 3/12), the other 9 STs contained only one strain. In addition, 4 new STs (containing GP004, GP012, GP050 and GP095) were observed and named as nST1, nST2, nST3, and nST4, respectively. By using goeBURST algorithm in phyloviz, only one clonal complex (nST1-CC, containing ST48 and the founder nST1) was observed among 10 STs (Figure 2B).

## 4 Conclusion

Our present study showed that ESBL-EC from giant pandas exhibited a diversity of ST clonal lineages and subtypes of *bla*_CTX-M_. ESBL-EC become a pool of ARGs, VAGs and MGEs that facilitate horizontal gene transfer mainly mediated by plasmids. Releasing captive giant pandas back into their natural habitat could potentially lead to the release of these bacteria into the environment, contributing to environmental pollution caused by AMR bacteria.

## 5 Discussion

The production of ESBLs is one of the most common markers of AMR in *Enterobacteriaceae* (25). ESBL-EC has been widely reported in captive wildlife, including giant pandas (5, 6, 14, 26, 27). In this study, we detected 12 ESBL-EC strains in captive giant pandas and found that the prevalence of ESBL-EC (10.26%, 12/117) was lower than that reported in other studies (26.04 and 80.00%, respectively) (9, 14). The emergence of ESBL-EC in captive giant pandas may originate from various sources, including exposure to antibiotics in captivity during veterinary care, cross-contamination from human contact, environmental reservoirs harboring resistant bacteria, and transmission from other animals (28). In addition, ESBL-EC has been widely detected in other wildlife, such as magnificent frigatebirds, carnivorous mammals (*Neovison vison* and *Martes foina*), owls, vultures and coatis (1, 29, 30). In our present study, no ESBL-EC was detected in 27 fecal samples from wild giant pandas. The difficulty in isolating ESBL-EC from wild pandas likely results from their limited exposure to human-related factors that contribute to antibiotic resistance, logistical challenges in obtaining samples non-invasively, and the potentially low abundance or intermittent shedding of these bacteria in wild populations (31). Nevertheless, continuous epidemiological surveillance for ESBL-EC in giant pandas are still required, especially as the giant panda reintroduction project in China is ongoing.

Among the ESBL-EC strains observed in our present study, 50.00% of the strains were MDR, which was lower than that in studies from other wild animals (69.05% ~ 100.00%) (3, 29, 32). The existence of MDR phenotypes revealed that the co-occurrence of ESBLs with other resistance traits in *E. coli* isolates results in the development of their resistance spectrum to β-lactams and other antimicrobial agents (33–35). To better understand the types of ESBLs, we further analyzed the ESBL genes in 12 ESBL-EC strains. Our result showed that *bla*_CTX-M_ (100.00%, 12/12) was the predominant ESBL gene. Sequence-based analysis showed 5 variants of *bla*_CTX-M_ (*bla*_CTX-M-55_, *bla*_CTX-M-13_, *bla*_CTX-M-27_, *bla*_CTX-M-14_ and *bla*_CTX-M-15_) exist in the 12 ESBL-EC, of which *bla*_CTX-M-55_ (33.33%, 4/12) was the most common. The prevalence of *bla*_CTX-M-55_ was also observed in other studies in ESBL-EC from diseased captive giant pandas (75.00%) (14), and other animals (swans, squirrel monkeys, black hat hanging monkeys, gibbon monkeys and phoenicopteridae, 34.80%), leading the authors to speculated that *bla*_CTX-M-55_ may become the major *bla*_CTX-M_ variant in Chinese zoo animals (6). The predominance of *bla*_CTX-M-55_ detected in our study provided further evidence for this speculation.

The spread of β-lactamases is often associated with plasmid-mediated horizontal transfer of ARGs encoding β-lactamase resistance, specifically the *bla*_CTX-M_ gene (33). In our study, conjugation experiments confirmed that the *bla*_CTX-M_ gene carried by ESBL-EC can be horizontally transferred by conjugation plasmids, and the transconjugants also showed ESBL-producing phenotypes. PCR-based replicon typing showed that the conjugative plasmids of ESBL-EC included IncFrepB, IncHI1, IncFIB, IncHI2, IncX, and IncFIA. These incompatibility-group types have also been identified in plasmids from ESBL-EC worldwide in previous studies (36–39).

Among the 12 ESBL-EC, the ARGs of *bla*_CTX − M_, *rmtB* and *sul2*, the VAGs of *papA*, fimC and *ompT*, and the MGEs of *ISEcp1*, *IS26* and *ISCR3/14* were all horizontally transferred which mediated by plasmid conjugation. All aminoglycoside (gentamicin and amikacin) resistant strains carrying the *rmtB* gene were co-transferred with the *bla*_CTX-M-55_ or *bla*_CTX-M-14_ gene. The other aminoglycoside-resistant encoding gene *armA*, which has been previously reported to be linked with *bla*_CTX-M_ and located in the same plasmid (40–42), while *armA* was not detected in our study. In addition, horizontal gene transfer facilitates the acquisition of virulence factors, and provides an evolutionary pathway for the development of pathogenicity (43). All of the *papA*, *fimC*, and *ompT* carried by ESBL-EC in this study can be horizontally transferred. In particular, the *papA* (encoding type P fimbriae) and *fimC* (encoding type I fimbriae) have been reported to be related to pathogenicity and colonization of fimbriae in extraintestinal infections caused by *E. coli* (44, 45). The *ompT* (encoding outer membrane protein T) has been reported to potentially contribute to bacterial cell attachment to host epithelial tissues (such as the urinary tract) and establishment a persistent bacterial infection (46). Therefore, co-transfer of *papA*, *fimC* and *ompT* with the *bla*_CTX-M_ gene may increase the pathogenicity of bacterial diseases and make them more difficulty to treat in captive giant pandas. It is worth noting that six (*papA*, *fimC*, *fyuA*, *irp2*, *sitA* and *ompT*) of the seven VAGs observed in strain GP022 were all successfully co-transferred with *bla*_CTX-M-15_. The *bla*_CTX-M-15_ gene has previously been reported to be extensively associated with highly virulent *E. coli* (such as B2-ST131 *E. coli*) (47), our present finding also suggests that the co-localization of VAGs and *bla*_CTX-M-15_ may potentially increase the virulence of *E. coli*. Regarding MGEs, all of the *ISEcp1* and *IS26* carried by ESBL-EC were co-transferred with *bla*_CTX-M_ gene in our study. It has been widely reported that *ISEcp1* and *IS26* are located upstream of *bla*_CTX-M_ and play a key role in the dissemination of *bla*_CTX-M_ (33, 48–50), and *ISEcp1* can enhance the expression of *bla*_CTX-M_ (49). Moreover, the TrbC protein is essential for the conjugative transfer of the IncF plasmid (51). In our present study, *trbC* was also observed to co-transfer with *bla*_CTX-M-14_ and *bla*_CTX-M-27_, leading us to deduce that *trbC* may be involved in the plasmid-mediated HGT of the *bla*_CTX-M_ gene between different strains.

The population structure of ESBL-EC clones can be determined by phylogenetic grouping and MLST (7). Our results showed that ESBL-EC belonged predominantly to group B2 (75.00%), which was consistent with previous studies from waterfowl birds, companion animals, and broiler chickens (52–54). We also used MLST to better understand the clonal lineages of the 12 ESBL-EC. Twelve ESBL-EC belonged to 10 different STs, including six known STs and four new STs. Three isolates detected in our study belonged to ST127, ST354 and ST648, which were among the top 20 ExPEC lineages worldwide and were responsible for the majority of extraintestinal diseases, contributing significantly to the global burden of infectious disease (55). In particular, the isolate (GP022) encoding *bla*_CTX-M-15_ belongs to clone B2-ST648, and clone ST648 is mostly combined with MDR and virulence, which is one of the most common international epidemic high-risk clone lineages at the human-animal-environmental interface worldwide (1, 56, 57). To the best of our knowledge, this is the first report of the *E. coli* B2-ST648 isolate encoding *bla*_CTX-M-15_ from captive giant pandas. In addition, the remaining STs (ST48, ST206, and ST1706) identified in our study have also been detected in *E. coli* from humans and other animals (6, 58–60). In general, ESBL-EC detected in captive giant pandas exhibited a diversity of clonal lineages, which may be due to the extensive spread of ESBL-EC mediated by the HGT of ESBL genes.

Our study revealed the diversity of ESBL-EC from captive giant pandas, along with their carriage of ARGs, VAGs and MGEs. This suggests that pandas in zoo environments could potentially serve as reservoirs for the spread of ARGs, posing risks to public health. Consequently, releasing ESBL-EC positive pandas from the zoo requires cautious consideration and thorough risk assessment to prevent the potential introduction of AMR bacteria into natural ecosystems.

## Data availability statement

All strain sequencing data has been deposited in the NCBI database, accessible using the accession numbers: PP988284-PP988307. Other data for this study are available upon reasonable request from the corresponding authors.

## Author contributions

HL: Data curation, Writing – original draft, Writing – review & editing. SF: Data curation, Writing – original draft. XZ: Conceptualization, Methodology, Software, Writing – original draft. YY: Data curation, Writing – original draft. WZ: Supervision, Writing – original draft. LW: Investigation, Visualization, Writing – original draft. CW: Supervision, Writing – original draft. ZiZ: Supervision, Writing – original draft. SZ: Software, Validation, Writing – original draft. YG: Software, Validation, Writing – original draft. GP: Software, Validation, Writing – original draft. YW: Investigation, Visualization, Writing – original draft. KZ: Investigation, Visualization, Writing – original draft. QY: Writing – review & editing. YL: Investigation, Visualization, Writing – original draft. KS: Methodology, Software, Writing – original draft, Conceptualization. ZhZ: Conceptualization, Methodology, Software, Writing – original draft, Writing – review & editing.

## Ethics statement

The animal study was approved by the Sichuan Agricultural University Institutional Animal Care and Use Committee (No. DYY-20130306). The study was conducted in accordance with the local legislation and institutional requirements.

## Footnotes

^1^ http://blast.ncbi.nlm.nih.gov

^2^ https://pubmlst.org
