# Supplementary material for: Antibiotic-resistant characteristics and horizontal gene transfer ability analysis of extended-spectrum β-lactamase-producing Escherichia coli isolated from giant pandas
Source: Front Vet Sci. 2024 Jul 26;11:1394814. doi: 10.3389/fvets.2024.1394814 (PMC11310934; doi:10.3389/fvets.2024.1394814)
Supplement: Supplementary file 1 [file Data_Sheet_1.docx]

>seq1[organism=Giant Panda Escherichia coli] Giant Panda Escherichia coli strain GP001, adenylate kinase gene.

TCTATTTCCGCAAATTATCTTGCCATTAACCGTTTCAGCCTCAGGTGCCTTTCTTGAGGCAATCGCCTGTTGGTGGTATCGTTTATCGCTTTTTCAAAAAATTCGACACATTTTAAGGGGATTTTCGCAATGCGTATCATTCTGCTTGGCGCTCCGGGCGCGGGGAAAGGGACTCAGGCTCAGTTCATCATGGAGAAATATGGTATTCCGCAAATCTCCACTGGCGATATGCTGCGTGCTGCGGTCAAATCTGGCTCCGAGCTGGGTAAACAAGCAAAAGACATTATGGATGCTGGCAAACTGGTCACCGACGAACTGGTGATCGCGCTGGTTAAAGAGCGCATTGCTCAGGAAGACTGCCGCAACGGTTTCCTGTTGGACGGCTTCCCGCGTACCATTCCGCAGGCAGACGCGATGAAAGAAGCGGGCATCAATGTTGATTACGTTCTGGAATTCGACGTACCGGACGAACTGATTGTTGACCGTATCGTAGGCCGCCGCGTTCACGCGCCGTCTGGTCGTGTTTATCACGTTAAATTCAATCCGCCGAAAGTAGAAGGCAAAGACGACGTTACCGGTGAAGAGCTGACTACCCGTAAAGACGATCAGGAAGAGACCGTACGTAAACGTCTGGTTGAATACCATCAGATGACTGCACCGCTGATCGGCTACTACTCCAAAGAAGCGGAAGCGGGTAACACCAAATACGCGAAAGTTGACGGCACCAAGCCGGTGGCTGAAGTTCGGCT

>seq2[organism=Giant Panda Escherichia coli] Giant Panda Escherichia coli strain GP003, adenylate kinase gene.

TATTTCCGCAAATTATCTCGCCATTAACCGTTTCAGCCTCAGGTGCCTTTCTTGAGGCAATCGCCTGTTGGTGGTATCGTTTATCGCTTTTTCAAAAAATTCGACACATTTTAAGGGGATTTTCGCAATGCGTATCATTCTGCTTGGCGCTCCGGGCGCGGGGAAAGGGACTCAGGCTCAGTTCATCATGGAGAAATATGGTATTCCGCAAATCTCCACTGGCGATATGCTGCGTGCTGCGGTCAAATCTGGCTCCGAGCTGGGTAAACAAGCAAAAGACATTATGGATGCTGGCAAACTGGTCACCGACGAACTGGTGATCGCGCTGGTTAAAGAGCGCATTGCTCAGGAAGACTGCCGTAATGGTTTCCTGTTGGACGGCTTCCCGCGTACCATTCCGCAGGCAGACGCGATGAAAGAAGCGGGCATCAATGTTGATTACGTTCTGGAATTCGACGTACCGGACGAACTGATTGTTGATCGTATCGTAGGCCGCCGCGTTCATGCGCCGTCTGGTCGTGTTTATCACGTTAAATTCAATCCGCCGAAAGTAGAAGGCAAAGACGACGTTACCGGTGAAGAACTGACTACCCGTAAAGACGATCAGGAAGAAACCGTGCGTAAACGTCTGGTTGAATACCATCAGATGACTGCACCGCTGATCGGCTACTACTCCAAAGAAGCGGAAGCGGGTAACACCAAATACGCGAAAGTTGACGGCACCAAGCCGGTTGCTGAAGTTCCCC

>seq3[organism=Giant Panda Escherichia coli] Giant Panda Escherichia coli strain GP004, adenylate kinase gene.

TATTTCCGCAAATTATCTCGCCATTAACCGTTTCAGCCTCAGGTGCCTTTCTTGAGGCAATCGCCTGTTGGTGGTATCGTTTATCGCTTTTTCAAAAAATTCGACACATTTTAAGGGGATTTTCGCAATGCGTATCATTCTGCTTGGCGCTCCGGGCGCGGGGAAAGGGACTCAGGCTCAGTTCATCATGGAGAAATATGGTATTCCGCAAATCTCCACTGGCGATATGCTGCGTGCTGCGGTCAAATCTGGCTCCGAGCTGGGTAAACAAGCAAAAGACATTATGGATGCTGGCAAACTGGTCACCGACGAACTGGTGATCGCGCTGGTTAAAGAGCGCATTGCTCAGGAAGACTGCCGTAATGGTTTCCTGTTGGACGGCTTCCCGCGTACCATTCCGCAGGCAGACGCGATGAAAGAAGCGGGCATCAATGTTGATTACGTTCTGGAATTCGACGTACCGGACGAACTGATTGTTGATCGTATCGTAGGCCGCCGCGTTCATGCGCCGTCTGGTCGTGTTTATCACGTTAAATTCAATCCGCCGAAAGTAGAAGGCAAAGACGACGTTACCGGTGAAGAACTGACTACCCGTAAAGACGATCAGGAAGAAACCGTGCGTAAACGTCTGGTTGAATACCATCAGATGACTGCACCGCTGATCGGCTACTACTCCAAAGAAGCGGAAGCGGGTAACACCAAATACGCGAAAGTTGACGGCACCAAGCCGGTTGCTGAAGTCGCGT

>seq4[organism=Giant Panda Escherichia coli] Giant Panda Escherichia coli strain GP012, adenylate kinase gene.

TTTTTACTCACTATTTGTATTTCCGCAAATTATCTCGCCATTAACCGTTTCAGCCTCAGGTGCCTTTCTTGAGGCAATCGCCTGTTGGTGGTATCGTTTATCGCTTTTTCAAAAAATTCGACACATTTTAAGGGGATTTTCGCAATGCGTATCATTCTGCTTGGCGCTCCGGGCGCGGGGAAAGGGACTCAGGCTCAGTTCATCATGGAGAAATATGGTATTCCGCAAATCTCCACTGGCGATATGCTGCGTGCTGCGGTCAAATCTGGCTCCGAGCTGGGTAAACAAGCAAAAGACATTATGGATGCTGGCAAACTGGTCACCGACGAACTGGTGATCGCGCTGGTTAAAGAGCGCATTGCTCAGGAAGACTGCCGCAACGGTTTCCTGTTGGACGGCTTCCCGCGTACCATTCCGCAGGCAGACGCGATGAAAGAAGCGGGCATCAATGTTGATTACGTTCTGGAATTCGACGTACCGGACGAACTGATTGTTGACCGTATCGTAGGCCGCCGCGTTCACGCGCCGTCTGGTCGTGTTTATCACGTTAAATTCAATCCGCCGAAAGTAGAAGGCAAAGACGACGTTACCGGTGAAGAGCTGACTACCCGTAAAGACGATCAGGAAGAGACCGTACGTAAACGTCTGGTTGAATACCATCAGATGACTGCACCGCTGATCGGCTACTACTCCAAAGAAGCGGAAGCGGGTAACACCAAATACGCGAAAGTTGACGGCACCAAGCCGGTTGCTGAAGTTCCC

>seq5[organism=Giant Panda Escherichia coli] Giant Panda Escherichia coli strain GP014, adenylate kinase gene.

TCTATTTCCGCAAATTATCTCGCCATTAACCGTTTCAGCCTCAGGTGCCTTTCTTGAGGCAATCGCCTGTTGGTGGTATCGTTTATCGCTTTTTCAAAAAATTCGACACATTTTAAGGGGATTTTCGCAATGCGTATCATTCTGCTTGGCGCTCCGGGCGCGGGGAAAGGGACTCAGGCTCAGTTCATCATGGAGAAATATGGTATTCCGCAAATCTCCACTGGCGATATGCTGCGTGCTGCGGTCAAATCTGGCTCCGAGCTGGGTAAACAAGCAAAAGACATTATGGATGCTGGCAAACTGGTCACCGACGAACTGGTGATCGCGCTGGTTAAAGAGCGCATTGCTCAGGAAGACTGCCGTAATGGTTTCCTGTTGGACGGCTTCCCGCGTACCATTCCGCAGGCAGACGCGATGAAAGAAGCGGGCATCAATGTTGATTACGTTCTGGAATTCGACGTACCGGACGAACTGATTGTTGATCGTATCGTAGGCCGCCGCGTTCATGCGCCGTCTGGTCGTGTTTATCACGTTAAATTCAATCCGCCGAAAGTAGAAGGCAAAGACGACGTTACCGGTGAAGAACTGACTACCCGTAAAGACGATCAGGAAGAAACCGTGCGTAAACGTCTGGTTGAATACCATCAGATGACTGCACCGCTGATCGGCTACTACTCCAAAGAAGCGGAAGCGGGTAACACCAAATACGCGAAAGTTGACGGCACCAAGCCGGTTGCTGAAGTCCC

>seq6[organism=Giant Panda Escherichia coli] Giant Panda Escherichia coli strain GP022, adenylate kinase gene.

TATTTCCGCAAATTATCTCGCCATTAACCGTTTCAGCCCCAGGTGCCTTTCTTGAGGCAATCGCTTGTTGGTGGTATCGTTTATCGCTTTTTCAAAAAATTCGACACATTTTAAGGGGATTTTCGCAATGCGTATCATTCTGCTTGGCGCTCCGGGCGCGGGGAAAGGGACTCAGGCTCAGTTCATCATGGAGAAATATGGTATTCCGCAAATCTCCACTGGCGATATGCTGCGTGCTGCGGTCAAATCTGGCTCCGAGCTGGGTAAACAAGCAAAAGACATTATGGATGCTGGCAAACTGGTCACCGACGAACTGGTGATCGCGCTGGTTAAAGAGCGCATTGCTCAGGAAGACTGCCGTAATGGTTTCCTGTTGGACGGCTTCCCGCGTACCATTCCGCAGGCAGACGCGATGAAAGAAGCGGGCATCAATGTTGATTACGTTCTGGAATTCGACGTACCGGACGAACTGATTGTTGATCGTATCGTCGGCCGCCGCGTTCATGCGCCGTCTGGTCGTGTTTATCACGTTAAATTCAATCCGCCTAAAGTAGAAGGCAAAGACGACGTTACCGGTGAAGAACTGACTACCCGTAAAGACGATCAGGAAGAAACCGTGCGTAAACGTCTGGTTGAATACCATCAGATGACTGCACCGCTGATCGGCTACTACTCCAAAGAAGCGGAAGCAGGTAACACCAAATACGCGAAAGTTGACGGCACCAAGCCGGTGGCTGAAGTTCGGC

>seq7[organism=Giant Panda Escherichia coli] Giant Panda Escherichia coli strain GP030, adenylate kinase gene.

TTCTATTCCGCAAATTATCTCGCCATTAACCGTTTCAGCCTCAGGTGCCTTTCTTGAGGCAATCGCCTGTTGGTGGTATCGTTTATCGCTTTTTCAAAAAATTCGACACATTTTAAGGGGATTTTCGCAATGCGTATCATTCTGCTTGGCGCTCCGGGCGCGGGGAAAGGGACTCAGGCTCAGTTCATCATGGAGAAATATGGTATTCCGCAAATCTCCACTGGCGATATGCTGCGTGCTGCGGTCAAATCTGGCTCCGAGCTGGGTAAACAAGCAAAAGACATTATGGATGCTGGCAAACTGGTCACCGACGAACTGGTGATCGCGCTGGTTAAAGAGCGCATTGCTCAGGAAGACTGCCGTAATGGTTTCCTGTTGGACGGCTTCCCGCGTACCATTCCGCAGGCAGACGCGATGAAAGAAGCGGGCATCAATGTTGATTACGTTCTGGAATTCGACGTACCGGACGAACTGATTGTTGATCGTATCGTAGGCCGCCGCGTTCATGCGCCGTCTGGTCGTGTTTATCACGTTAAATTCAATCCGCCGAAAGTAGAAGGCAAAGACGACGTTACCGGTGAAGAACTGACTACCCGTAAAGACGATCAGGAAGAAACCGTGCGTAAACGTCTGGTTGAATACCATCAGATGACTGCACCGCTGATCGGCTACTACTCCAAAGAAGCGGAAGCGGGTAACACCAAATACGCGAAAGTTGACGGCACCAAGCCGGTTGCTGAAGTTCCCC

>seq8[organism=Giant Panda Escherichia coli] Giant Panda Escherichia coli strain GP032, adenylate kinase gene.

TCTATTTCCGCAAATTATCTCGCCATTAACCGTTTCAGCCCCAGGTGCCTTTCTTGAGGCAATCGCTTGTTGGTGGTATCGTTTATCGCTTTTTCAAAAAATTCGACACATTTTAAGGGGATTTTCGCAATGCGTATCATTCTGCTTGGCGCTCCGGGCGCGGGGAAAGGGACTCAGGCTCAGTTCATCATGGAGAAATATGGTATTCCGCAAATCTCCACTGGCGATATGCTGCGTGCTGCGGTCAAATCTGGCTCCGAGCTGGGTAAACAAGCAAAAGACATTATGGATGCTGGCAAACTGGTCACCGACGAACTGGTGATCGCGCTGGTTAAAGAGCGCATTGCTCAGGAAGACTGCCGTAATGGTTTCCTGTTGGACGGCTTCCCGCGTACCATTCCGCAGGCAGACGCGATGAAAGAAGCGGGCATCAATGTTGATTACGTTCTGGAATTCGACGTACCGGACGAACTGATTGTTGATCGTATTGTAGGCCGCCGCGTTCATGCGCCGTCTGGTCGTGTTTATCACGTTAAATTCAATCCGCCTAAAGTAGAAGGCAAAGACGACGTTACCGGTGAAGAACTGACTACCCGTAAAGACGATCAGGAAGAAACCGTGCGTAAACGTCTGGTTGAATACCATCAGATGACTGCACCGCTGATCGGCTACTACTCCAAAGAAGCGGAAGCAGGTAACACCAAATACGCGAAAGTTGACGGCACCAAGCCGGTTGCTGAAGTTCCCTCTG

>seq9[organism=Giant Panda Escherichia coli] Giant Panda Escherichia coli strain GP050, adenylate kinase gene.

ATTTCCGCAAATTATCTTGCCATTAACCGTTTCAGCCTCAGGTGCCTTTCTTGAGGCAATCGCCTGTTGGTGGTATCGTTTATCGCTTTTTCAAAAAATTCGACACATTTTAAGGGGATTTTCGCAATGCGTATCATTCTGCTTGGCGCTCCGGGCGCGGGGAAAGGGACTCAGGCTCAGTTCATCATGGAGAAATATGGTATTCCGCAAATCTCCACTGGCGATATGCTGCGTGCTGCGGTCAAATCTGGCTCCGAGCTGGGTAAACAAGCAAAAGACATTATGGATGCTGGCAAACTGGTCACCGACGAACTGGTGATCGCGCTGGTTAAAGAGCGCATTGCTCAGGAAGACTGCCGCAACGGTTTCCTGTTGGACGGCTTCCCGCGTACCATTCCGCAGGCAGACGCGATGAAAGAAGCGGGCATCAATGTTGATTACGTTCTGGAATTCGACGTACCGGACGAACTGATTGTTGACCGTATCGTAGGCCGCCGCGTTCACGCGCCGTCTGGTCGTGTTTATCACGTTAAATTCAATCCGCCGAAAGTAGAAGGCAAAGACGACGTTACCGGTGAAGAACTGACTACCCGTAAAGACGATCAGGAAGAGACCGTACGTAAACGTCTGGTTGAATACCATCAGATGACTGCACCGCTGATCGGCTACTACTCCAAAGAAGCGGAAGCGGGTAACACCAAATACGCGAAAGTTGACGGCACCAAGCCGGTGGCTGAAGTTCCCC

>seq10[organism=Giant Panda Escherichia coli] Giant Panda Escherichia coli strain GP065, adenylate kinase gene.

TTCTATTCCGCAAATTATCTCGCCATTAACCGTTTCAGCCTCAGGTGCCTTTCTTGAGGCAATCGCCTGTTGGTGGTATCGTTTATCGCTTTTTCAAAAAATTCGACACATTTTAAGGGGATTTTCGCAATGCGTATCATTCTGCTTGGCGCTCCGGGCGCGGGGAAAGGGACTCAGGCTCAGTTCATCATGGAGAAATATGGTATTCCGCAAATCTCCACTGGCGATATGCTGCGTGCTGCGGTCAAATCTGGCTCCGAGCTGGGTAAACAAGCAAAAGACATTATGGATGCTGGCAAACTGGTCACCGACGAACTGGTGATCGCGCTGGTTAAAGAGCGCATTGCTCAGGAAGACTGCCGTAATGGTTTCCTGTTGGACGGCTTCCCGCGTACCATTCCGCAGGCAGACGCGATGAAAGAAGCGGGCATCAATGTTGATTACGTTCTGGAATTCGACGTACCGGACGAACTGATTGTTGATCGTATCGTAGGCCGCCGCGTTCATGCGCCGTCTGGTCGTGTTTATCACGTTAAATTCAATCCGCCGAAAGTAGAAGGCAAAGACGACGTTACCGGTGAAGAACTGACTACCCGTAAAGACGATCAGGAAGAAACCGTGCGTAAACGTCTGGTTGAATACCATCAGATGACTGCACCGCTGATCGGCTACTACTCCAAAGAAGCGGAAGCGGGTAACACCAAATACGCGAAAGTTGACGGCACCAAGCCGGTTGCTGAAGTTCCCC

>seq11[organism=Giant Panda Escherichia coli] Giant Panda Escherichia coli strain GP095, adenylate kinase gene.

TTCTATTTCCGCAAATTATCTCGCCATTAACCGTTTCAGCCCCAGGTGCCTTTCTTGAGGCAATCGCTTGTTGGTGGTATCGTTTATCGCTTTTTCAAAAAATTCGACACATTTTAAGGGGATTTTCGCAATGCGTATCATTCTGCTTGGCGCTCCGGGCGCGGGGAAAGGGACTCAGGCTCAGTTCATCATGGAGAAATATGGTATTCCGCAAATCTCCACTGGCGATATGCTGCGTGCCGCAGTCAAATCTGGCTCCGAGCTGGGTAAACAAGCTAAAGACATTATGGACGCTGGCAAACTGGTCACCGATGAACTGGTGATCGCGCTGGTTAAAGAGCGCATTGCTCAGGAAGATTGCCGTAATGGTTTTCTGTTGGACGGCTTCCCGCGTACCATTCCGCAGGCAGACGCGATGAAAGAAGCGGGCATCAATGTTGATTACGTTCTGGAATTCGACGTACCGGACGAGCTGATTGTTGATCGTATCGTCGGTCGCCGCGTTCACGCGCCGTCTGGTCGTGTATATCACGTTAAATTCAATCCGCCGAAAGTAGAAGGCAAAGACGACGTTACCGGTGAAGAACTGACTACCCGTAAAGACGATCAGGAAGAAACCGTACGTAAGCGTCTGGTTGAATACCATCAGATGACTGCACCGCTGATTGGCTACTACTCCAAAGAAGCAGAAGCGGGTAACACCAAATACGCGAAAGTTGACGGCACCAAGCCGGTTGCTGAAGTTCGGTTGA

>seq12[organism=Giant Panda Escherichia coli] Giant Panda Escherichia coli strain GP101, adenylate kinase gene.

ATTCCGCAAATTATCTCGCCATTAACCGTTTCAGCCTCAGGTGCCTTTCTTGAGGCAATCGCCTGTTGGTGGTATCGTTTATCGCTTTTTCAAAAAATTCGACACATTTTAAGGGGATTTTCGCAATGCGTATCATTCTGCTTGGCGCTCCGGGCGCGGGGAAAGGGACTCAGGCTCAGTTCATCATGGAGAAATATGGTATTCCGCAAATCTCCACTGGCGATATGCTGCGTGCTGCGGTCAAATCTGGCTCCGAGCTGGGTAAACAAGCAAAAGACATTATGGATGCTGGCAAACTGGTCACCGACGAACTGGTGATCGCGCTGGTTAAAGAGCGCATTGCTCAGGAAGACTGCCGTAATGGTTTCCTGTTGGACGGCTTCCCGCGTACCATTCCGCAGGCAGACGCGATGAAAGAAGCGGGCATCAATGTTGATTACGTTCTGGAATTCGACGTACCGGACGAACTGATTGTTGATCGTATCGTAGGCCGCCGCGTTCATGCGCCGTCTGGTCGTGTTTATCACGTTAAATTCAATCCGCCGAAAGTAGAAGGCAAAGACGACGTTACCGGTGAAGAACTGACTACCCGTAAAGACGATCAGGAAGAAACCGTGCGTAAACGTCTGGTTGAATACCATCAGATGACTGCACCGCTGATCGGCTACTACTCCAAAGAAGCGGAAGCGGGTAACACCAAATACGCGAAAGTTGACGGCACCAAGCCGGTTGCTGAAGTTCCC
