# Supplementary material for: Antibiotic-resistant characteristics and horizontal gene transfer ability analysis of extended-spectrum β-lactamase-producing Escherichia coli isolated from giant pandas
Source: Front Vet Sci. 2024 Jul 26;11:1394814. doi: 10.3389/fvets.2024.1394814 (PMC11310934; doi:10.3389/fvets.2024.1394814)
Supplement: Supplementary file 2 [file Data_Sheet_2.docx]

>seq1[organism=Giant Panda Escherichia coli] Giant Panda Escherichia coli strain GP001, class II fumarate hydratase gene.

TTCCACGCTTCAAATTTGTTCGGTGCGGTAACAAACGGTGCACAGGTAATGACTGCCAGTTCATCTGCTACGCGACGCGCATATTCCGGATGGGTATTTAGTCCAGTACCCACCGCTGTACCGCCCAGAGCCAGTTCCGCTACGTGAGGCAGGCTGTATTCGATATGTTTGAGATTATGCTCCAGCATCGCTACCCAGCCGGAAATCTCCTGCCCCAGCGTTAACGGCGTCGCGTCCTGCAAGTGGGTACGACCGATTTTGACGATATCGGCAAATGCGCGGGATTTTTCACTCAGCGTCTGGGTCAGGGTTTTAAGTTGTGGAATGAGTTGCTTGCGCAGCGCCAGTAGTGCCGCAACGTGCATCGCCGTCGGAAAGACATCGTTGGAACTTTGGCTTTTGTTCACGTCGTCGTTAGGGTGAACTTTACGTTCCATCCCGCGCACGCCGCCGAGTAATTCACTGGCCCGGTTAGCCAGCACTTCATTCATGTTCATGTTACTTTGCGTGCCGGAGCCGGTCTGCCAGATAGCCAGCGGGAATTCGTCGTCATGCTGTCCTGCCAGTACTTCATCCGCCGCCTGCCGAATGGCGCTCGCTTTCTCTTCAGACAACAAGCCTAAATCTTCATTAACTTTTGCCGCTGCGCGCTTGGTTAGCGCCAGCGCATGAATCAGTGAGGTGGGCATTTTCTCCGTCGAAATGCGGAAATGCTCCAGCGAGCGCTGAGTTTGTGCGCCCCACAGCTTATCTGCCGGGACATCAATCGCTCCCATCGAATCTTTTCCC

>seq2[organism=Giant Panda Escherichia coli] Giant Panda Escherichia coli strain GP003, class II fumarate hydratase gene.

CCACGCTTCAAATTTGTTCGGCGCGGTAACAAACGGTGCACAGGTAATGACTGCCAGTTCATCTGCTACGCGACGCGCATACTCCGGATGGGTATTTAGTCCAGTACCCACCGCTGTACCGCCAAGAGCCAGTTCCGCTACGTGAGGCAGGCTGTATTCGATATGTTTGAGATTATGCTCGAGCATCGCTACCCAGCCGGAAATCTCCTGCCCCAGCGTTAACGGCGTGGCATCCTGCAAGTGAGTACGACCAATTTTGACGATATCGGCAAAAGCACGGGATTTCTCATTCAGTGTCTGTGTCAGGGTTTTAAGCTGAGGAATGAGTTGCTTGCGCAGCGCCAGCAGCGCCGCAACGTGCATCGCCGTCGGAAAGACATCGTTGGAACTTTGGCTTTTGTTCACGTCGTCGTTAGGGTGAACTTTACGTTCCATCCCGCGCACACCGCCGAGTAATTCACTGGCCCGGTTAGCCAGCACTTCGTTCATGTTCATGTTACTTTGCGTGCCGGAGCCGGTCTGCCAGATAGCCAGCGGGAATTCGTCGTCATGCTGTCCTGCCAGTACTTCATCCGCCGCCTGACGAATGGCGCTCGCTTTCTCTTCAGACAACAAGCCTAAATCTTCATTAACTTTTGCCGCTGCACGCTTGGTTAGCGCCAGCGCATGAATCAGTGAGGTGGGCATTTTCTCCGTCGAAATGCGGAAATGCTCCAGCGAGCGTTGAGTTTGTGCGCCCCACAGCTTATCTGCCGGGACATCAATCGCCCCCATCGAATCTTTTG

>seq3[organism=Giant Panda Escherichia coli] Giant Panda Escherichia coli strain GP004, class II fumarate hydratase gene.

TTCCACGCTTCAAATTTGTTCGGCGCGGTAACAAACGGTGCACAGGTAATGACTGCCAGTTCATCTGCTACGCGACGCGCATACTCCGGATGGGTATTTAGTCCAGTACCCACCGCTGTACCGCCAAGAGCCAGTTCCGCTACGTGAGGCAGGCTGTATTCGATATGTTTGAGATTATGCTCGAGCATCGCTACCCAGCCGGAAATCTCCTGCCCCAGCGTTAACGGCGTGGCATCCTGCAAGTGAGTACGACCAATTTTGACGATATCGGCAAAAGCACGGGATTTCTCATTCAGTGTCTGTGTCAGGGTTTTAAGCTGAGGAATGAGTTGCTTGCGCAGCGCCAGCAGCGCCGCAACGTGCATCGCCGTCGGAAAGACATCGTTGGAACTTTGGCTTTTGTTCACGTCGTCGTTAGGGTGAACTTTACGTTCCATCCCGCGCACACCGCCGAGTAATTCACTGGCCCGGTTAGCCAGCACTTCGTTCATGTTCATGTTACTTTGCGTGCCGGAGCCGGTCTGCCAGATAGCCAGCGGGAATTCGTCGTCATGCTGTCCTGCCAGTACTTCATCCGCCGCCTGACGAATGGCGCTCGCTTTCTCTTCAGACAACAAGCCTAAATCTTCATTAACTTTTGCCGCTGCACGCTTGGTTAGCGCCAGCGCATGAATCAGTGAGGTGGGCATTTTCTCCGTCGAAATGCGGAAATGCTCCAGCGAGCGTTGAGTTTGTGCGCCCCACAGCTTATCTGCCGGGACATCAATCGCCCCCATCGAATCTTTTG

>seq4[organism=Giant Panda Escherichia coli] Giant Panda Escherichia coli strain GP012, class II fumarate hydratase gene.

CACGCTTCAAATTTGTTCGGTGCGGTAACAAACGGTGCACAGGTAATGACTGCCAGTTCATCTGCTACGCGACGCGCATACTCCGGATGGGTATTTAGTCCAGTACCCACCGCTGTACCGCCCAGAGCCAGTTCCGCTACGTGAGGCAGGCTGTATTCGATATGTTTGAGATTATGCTCCAGCATCGCTACCCAGCCGGAAATCTCCTGCCCCAGCGTTAACGGCGTGGCGTCCTGCAAGTGGGTACGACCGATTTTGACGATATCAGCAAATGCGCGGGATTTTTCACTCAGCGTCTGGGTCAGGGTTTTAAGTTGTGGAATGAGTTGCTTGCGCAGCGCCAGTAGTGCCGCAACGTGCATCGCCGTCGGAAAGACATCGTTGGAACTTTGGCTTTTGTTCACGTCGTCGTTAGGGTGAACTTTACGTTCCATCCCGCGCACGCCACCGAGTAATTCACTGGCCCGGTTAGCCAGCACTTCGTTCATATTCATGTTACTTTGCGTGCCGGAGCCGGTCTGCCAGATAGCCAGCGGGAATTCGTCGTCATGCTGTCCTGCCAGTACTTCATCCGCCGCCTGCCGAATGGCGCTCGCTTTCTCTTCAGACAACAAGCCTAAATCTTCATTAACTTTTGCCGCTGCGCGCTTGGTTAGCGCCAGCGCATGAATCAGTGAGGTGGGCATTTTCTCCGTCGAAATGCGGAAATGCTCCAGCGAGCGCTGAGTTTGTGCGCCCCACAGCTTATCTGCCGGGACATCAATCGCTCCCATCGAATCTTTC

>seq5[organism=Giant Panda Escherichia coli] Giant Panda Escherichia coli strain GP014, class II fumarate hydratase gene.

TGTCCACGCTTCAAATTTGTTCGGCGCGGTAACAAACGGTGCACAGGTAATGACTGCCAGTTCATCTGCTACGCGACGCGCATACTCCGGATGGGTATTTAGTCCAGTACCCACCGCTGTACCGCCAAGAGCCAGTTCCGCTACGTGAGGCAGGCTGTATTCGATATGTTTGAGATTATACTCGAGCATCGCTACCCAGCCGGAAATCTCCTGCCCCAGTGTTAGCGGCGTGGCGTCCTGCAAGTGGGTTCGACCGATTTTGACGATATCGGCAAATGCACGCGATTTTTCACTCAGCGTCTGTGTCAGGGTTTTAAGCTGCGGAATGAGTTGCTTGCGCAGCGCCAGCAGCGCCGCAACGTGCATCGCCGTCGGAAAGACATCGTTGGAACTTTGGCTTTTGTTCACGTCGTCGTTAGGGTGAACTTTACGTTCCATCCCGCGCACGCCGCCGAGTAATTCACTGGCCCGGTTAGCCAGCACTTCGTTCATGTTCATGTTACTTTGCGTGCCGGAGCCGGTCTGCCAGATAGCCAGCGGGAATTCGTCGTCATGCTGTCCTGCCAGTACTTCATCCGCCGCCTGCCGAATGGCGCTCGCTTTCTCTTCAGACAACAAGCCTAAATCTTCATTAACTTTTGCCGCTGCGCGCTTGGTTAGCGCCAGCGCATGAATCAGTGAGGTGGGCATTTTCTCCGTCGAAATGCGGAAATGCTCCAGCGAGCGTTGAGTTTGTGCGCCCCACAGCTTATCTGCCGGGACATCAATCGCCCCCATCGAATCTTTC

>seq6[organism=Giant Panda Escherichia coli] Giant Panda Escherichia coli strain GP022, class II fumarate hydratase gene.

GACGCTTCAAATTTGTTCGGCGCGGTAACAAACGGTGCACAGGTAATGACTGCCAGTTCATCTGCTACGCGACGCGCATACTCCGGATGGGTATTTAGTCCAGTACCCACCGCTGTACCGCCAAGAGCCAGTTCCGCTACGTGAGGCAGGCTGTATTCGATATGTTTGAGATTATGCTCGAGCATCGCTACCCAGCCGGAAATCTCCTGCCCTAGTGTTAGCGGCGTGGCGTCCTGCAAGTGGGTTCGACCGATTTTGACGATATCGGCAAATGCACGCGATTTTTCACTCAGTGTCTGTGTCAGGGTTTTAAGCTGCGGAATGAGTTGCTTGCGCAGCGCCAGCAGCGCCGCAACGTGCATCGCCGTCGGAAAGACATCGTTGGAACTTTGGCTTTTGTTCACGTCGTCGTTAGGGTGAACTTTACGTTCCATCCCGCGCACGCCGCCGAGTAATTCACTGGCCCGGTTAGCCAGCACTTCGTTCATGTTCATGTTACTTTGCGTGCCGGAGCCGGTCTGCCAGATAGCCAGCGGGAATTCGTCGTCATGCTGTCCTGCCAGTACTTCATCCGCCGCCTGCCGAATGGCGCTCGCTTTCTCTTCAGACAACAAGCCTAAATCTTCATTAACTTTTGCCGCTGCGCGCTTGGTTAGCGCCAGCGCATGAATCAGTGAGGTGGGCATTTTCTCCGTCGAAATGCGGAAATGCTCCAGCGAGCGTTGAGTTTGTGCGCCCCACAGCTTATCTGCCGGGACATCAATCGCCCCCATCGAATCTTTC

>seq7[organism=Giant Panda Escherichia coli] Giant Panda Escherichia coli strain GP030, class II fumarate hydratase gene.

GTTCACGCTTCAAATTTGTTCGGCGCGGTAACAAACGGTGCACAGGTAATGACTGCCAGTTCATCTGCTACGCGACGCGCATACTCCGGATGGGTATTTAGTCCAGTACCCACCGCTGTACCGCCAAGAGCCAGTTCCGCTACGTGAGGCAGGCTGTATTCGATATGTTTGAGATTATGCTCGAGCATCGCTACCCAGCCGGAAATCTCCTGCCCCAGCGTTAACGGCGTGGCATCCTGCAAGTGAGTACGACCAATTTTGACGATATCGGCAAAAGCACGGGATTTCTCATTCAGTGTCTGTGTCAGGGTTTTAAGCTGAGGAATGAGTTGCTTGCGCAGCGCCAGCAGCGCCGCAACGTGCATCGCCGTCGGAAAGACATCGTTGGAACTTTGGCTTTTGTTCACGTCGTCGTTAGGGTGAACTTTACGTTCCATCCCGCGCACACCGCCGAGTAATTCACTGGCCCGGTTAGCCAGCACTTCGTTCATGTTCATGTTACTTTGCGTGCCGGAGCCGGTCTGCCAGATAGCCAGCGGGAATTCGTCGTCATGCTGTCCTGCCAGTACTTCATCCGCCGCCTGACGAATGGCGCTCGCTTTCTCTTCAGACAACAAGCCTAAATCTTCATTAACTTTTGCCGCTGCACGCTTGGTTAGCGCCAGCGCATGAATCAGTGAGGTGGGCATTTTCTCCGTCGAAATGCGGAAATGCTCCAGCGAGCGTTGAGTTTGTGCGCCCCACAGCTTATCTGCCGGGACATCAATCGCCCCCATCGAATCTTT

>seq8[organism=Giant Panda Escherichia coli] Giant Panda Escherichia coli strain GP032, class II fumarate hydratase gene.

CACGCTTCAAATTTGTTCGGCGCGGTAACAAACGGAGCGCAGGTAATGACTGCCAGTTCATCTGCTACTCGACGCGCATACTCCGGATGGGTATTTAGTCCAGTACCCACCGCTGTACCGCCAAGAGCCAGTTCCGCTACATGCGGCAGGCTGTATTCGATATGTTTGAGATTATGCTCGAGCATCGCTACCCAGCCGGAAATCTCCTGCCCCAGTGTTAGCGGCGTGGCATCCTGCAAGTGGGTTCGACCGATTTTGACGATATCGGCAAATGCACGGGATTTCTCATTCAGTGTCTGTGTCAGGGTTTTAAGCTGCGGAATGAGTTGCTTGCGCAGCGCCAGTAGCGCCGCAACGTGCATCGCCGTCGGAAAGACATCATTGGAACTTTGGCTTTTGTTCACGTCGTCGTTAGGGTGAACTTTACGCTCCATCCCGCGCACGCCGCCAAGTAATTCACTGGCCCGATTAGCCAGCACTTCGTTCATGTTCATATTACTTTGCGTGCCGGAGCCGGTCTGCCAGATAGCCAGCGGGAATTCGTCGTCATGCTGTCCTGCCAGTACTTCATCCGCCGCCTGACGAATGGCGCTCGCTTTCTCTTCAGACAACAAGCCTAAATCTTCATTAACTTTTGCCGCTGCGCGCTTGGTTAGTGCCAGCGCATGAATCAGTGAGGTGGGCATTTTCTCCGTCGAAATGCGGAAATGCTCCAGCGAGCGTTGAGTTTGTGCGCCCCACAGCTTATCTGCCGGGACATCAATCGCCCCCATCGAATCTTTT

>seq9[organism=Giant Panda Escherichia coli] Giant Panda Escherichia coli strain GP050, class II fumarate hydratase gene.

TTGCCACGCTTCAAATTTGTTCGGCGCGGTAACAAACGGTGCACAGGTAATGACTGCCAGTTCATCTGCTACGCGACGCGCATATTCCGGATGGGTATTTAGTCCAGTACCCACCGCTGTACCGCCCAGAGCCAGTTCCGCTACGTGAGGCAGGCTGTATTCGATATGTTTGAGATTATGCTCCAGCATCGCTACCCAGCCGGAAATCTCCTGCCCCAGCGTTAACGGCGTGGCGTCCTGCAAGTGGGTACGACCGATTTTGACGATATCGGCAAATGCGCGGGATTTTTCACTCAGCGTCTGGGTCAGGGTTTTAAGTTGTGGAATGAGTTGCTTGCGCAGCGCCAGCAGCGCCGCAACGTGCATCGCCGTCGGAAAGACATCGTTGGAACTTTGGCTTTTGTTCACGTCGTCGTTAGGGTGAACTTTACGTTCCATCCCGCGCACGCCGCCGAGTAATTCACTGGCCCGGTTAGCCAGCACTTCGTTCATGTTCATGTTACTTTGCGTGCCGGAGCCGGTCTGCCAGATAGCCAACGGGAATTCGTCGTCATGCTGTCCTGCCAGTACTTCATCCGCCGCCTGCCGAATGGCGCTCGCTTTCTCTTCAGACAACAAGCCTAAATCTTCATTAACTTTTGCCGCTGCGCGCTTGGTTAGCGCCAGCGCATGAATCAGTGAGGTGGGCATTTTCTCCGTCGAAATGCGGAAATGCTCCAGCGAGCGTTGAGTTTGTGCGCCCCACAGCTTATCTGCCGGGACATCAATCGCCCCCATCGAATCTTTT

>seq10[organism=Giant Panda Escherichia coli] Giant Panda Escherichia coli strain GP065, class II fumarate hydratase gene.

GTCCACGCTTCAAATTTGTTCGGCGCGGTAACAAACGGTGCACAGGTAATGACTGCCAGTTCATCTGCTACGCGACGCGCATACTCCGGATGGGTATTTAGTCCAGTACCCACCGCTGTACCGCCAAGAGCCAGTTCCGCTACGTGAGGCAGGCTGTATTCGATATGTTTGAGATTATGCTCGAGCATCGCTACCCAGCCGGAAATCTCCTGCCCCAGCGTTAACGGCGTGGCATCCTGCAAGTGAGTACGACCAATTTTGACGATATCGGCAAAAGCACGGGATTTCTCATTCAGTGTCTGTGTCAGGGTTTTAAGCTGAGGAATGAGTTGCTTGCGCAGCGCCAGCAGCGCCGCAACGTGCATCGCCGTCGGAAAGACATCGTTGGAACTTTGGCTTTTGTTCACGTCGTCGTTAGGGTGAACTTTACGTTCCATCCCGCGCACACCGCCGAGTAATTCACTGGCCCGGTTAGCCAGCACTTCGTTCATGTTCATGTTACTTTGCGTGCCGGAGCCGGTCTGCCAGATAGCCAGCGGGAATTCGTCGTCATGCTGTCCTGCCAGTACTTCATCCGCCGCCTGACGAATGGCGCTCGCTTTCTCTTCAGACAACAAGCCTAAATCTTCATTAACTTTTGCCGCTGCACGCTTGGTTAGCGCCAGCGCATGAATCAGTGAGGTGGGCATTTTCTCCGTCGAAATGCGGAAATGCTCCAGCGAGCGTTGAGTTTGTGCGCCCCACAGCTTATCTGCCGGGACATCAATCGCCCCCATCGAATCTTTTGC

>seq11[organism=Giant Panda Escherichia coli] Giant Panda Escherichia coli strain GP095, class II fumarate hydratase gene.

TTCCACGCTTCAAATTTGTTCGGCGCGGTAACAAACGGTGCACAGGTAATGACTGCCAGTTCATCTGCTACGCGACGCGCATACTCCGGATGGGTATTTAGTCCAGTACCCACCGCTGTACCGCCAAGAGCCAGTTCCGCTACGTGAGGCAGGCTGTATTCGATATGTTTGAGATTATGCTCGAGCATCGCTACCCAGCCGGAAATCTCCTGCCCTAGTGTTAGCGGCGTGGCGTCCTGCAAGTGGGTTCGACCGATTTTGACGATATCGGCAAATGCACGCGATTTTTCACTCAGTGTCTGTGTCAGGGTTTTAAGCTGCGGAATGAGTTGCTTGCGCAGCGCCAGCAGCGCCGCAACGTGCATCGCCGTCGGAAAGACATCGTTGGAACTTTGGCTTTTGTTCACGTCGTCGTTAGGGTGAACTTTACGTTCCATCCCGCGCACGCCGCCGAGTAATTCACTGGCCCGGTTAGCCAGCACTTCGTTCATGTTCATGTTACTTTGCGTGCCGGAGCCGGTCTGCCAGATAGCCAGCGGGAATTCGTCGTCATGCTGTCCTGCCAGTACTTCATCCGCCGCCTGCCGAATGGCGCTCGCTTTCTCTTCAGACAACAAGCCTAAATCTTCATTAACTTTTGCCGCTGCGCGCTTGGTTAGCGCCAGCGCATGAATCAGTGAGGTGGGCATTTTCTCCGTCGAAATGCGGAAATGCTCCAGCGAGCGTTGAGTTTGTGCGCCCCACAGCTTATCTGCCGGGACATCAATCGCCCCCATCGAATCTTTTCG

>seq12[organism=Giant Panda Escherichia coli] Giant Panda Escherichia coli strain GP101, class II fumarate hydratase gene.

TTTCCACGCTTCAAATTTGTTCGGCGCGGTAACAAACGGTGCACAGGTAATGACTGCCAGTTCATCTGCTACGCGACGCGCATACTCCGGATGGGTATTTAGTCCAGTACCCACCGCTGTACCGCCAAGAGCCAGTTCCGCTACGTGAGGCAGGCTGTATTCGATATGTTTGAGATTATGCTCGAGCATCGCTACCCAGCCGGAAATCTCCTGCCCCAGTGTTAGCGGCGTGGCATCCTGCAAGTGGGTTCGACCGATTTTGACGATATCGGCAAATGCACGCGATTTTTCACTCAGTGTCTGTGTCAGGGTTTTAAGCTGCGGAATGAGTTGCTTGCGCAGCGCCAGCAGCGCCGCAACGTGCATCGCCGTCGGAAAGACATCGTTGGAACTTTGGCTTTTGTTCACGTCGTCGTTAGGGTGAACTTTACGTTCCATCCCGCGCACGCCGCCGAGTAATTCACTGGCCCGGTTAGCCAGCACTTCGTTCATGTTCATGTTACTTTGCGTGCCGGAGCCGGTCTGCCAGATAGCCAGCGGGAATTCGTCGTCATGCTGTCCTGCCAGTACTTCATCCGCCGCCTGCCGAATGGCGCTCGCTTTCTCTTCAGACAACAAGCCTAAATCTTCATTAACTTTTGCCGCTGCGCGCTTGGTTAGCGCCAGCGCATGAATCAGTGAGGTGGGCATTTTCTCCGTCGAAATGCGGAAATGCTCCAGCGAGCGTTGAGTTTGTGCGCCCCACAGCTTATCTGCCGGGACATCAATCGCCCCCATCGAATCTTCCC
