# Supplementary material for: Antibiotic-resistant characteristics and horizontal gene transfer ability analysis of extended-spectrum β-lactamase-producing Escherichia coli isolated from giant pandas
Source: Front Vet Sci. 2024 Jul 26;11:1394814. doi: 10.3389/fvets.2024.1394814 (PMC11310934; doi:10.3389/fvets.2024.1394814)
Supplement: Supplementary file 3 [file Data_Sheet_3.docx]

>seq1[organism=Giant Panda Escherichia coli] Giant Panda Escherichia coli strain GP001, DNA topoisomerase (ATP-hydrolyzing) subunit B gene.

TTCGGGCGAACCAACCATGACGGCACCGGTCTGCACCACATGGTATTCGAGGTGGTAGATAACGCTATCGACGAAGCGCTCGCGGGTCACTGTAAAGAAATTATCGTCACCATTCACGCCGATAACTCTGTCTCCGTACAGGATGATGGGCGCGGCATTCCGACCGGTATTCACCCGGAAGAGGGCGTATCGGCGGCGGAAGTGATCATGACCGTTCTGCACGCAGGCGGTAAATTTGACGATAACTCCTATAAAGTGTCCGGCGGTCTGCACGGCGTTGGTGTTTCGGTAGTAAACGCCCTGTCGCAAAAACTGGAGCTGGTTATCCAGCGCGAGGGTAAAATTCACCGTCAGATCTACGAACACGGTGTACCGCAGGCCCCGCTGGCGGTTACCGGCGAGACTGAAAAAACCGGCACTATGGTGCGTTTCTGGCCAAGCCTTGAAACCTTCACCAATGTGACCGAGTTCGAATATGACATTCTGGCGAAACGTCTGCGTGAGTTGTCGTTCCTCAACTCCGGCGTTTCCATTCGTCTGCGCGACAAGCGCGACGGCAAAGAAGACCACTTCCACTATGAAGGCGGCATCAAGGCGTTCGTTGAATATCTGAACAAGAACAAAACGCCGATCCACCCGAATATCTTCTACTTCTCCACCGAAAAAGACGGTATTGGCGTCGAAGTGGCGTTGCAGTGGAACGATGGCTTCCAGGAAAACATCTACTGCTTTACCAACAACATTCCGCAGCGTGACGGCGGTACTCACCTGGCAGGTTTCCGTGCGGCGATGACCCGTACTCTGAACGCCTACATGGACAAAGAAGGCTACAGCAAAAAAGCCAAAGTCAGCGCCACCGGTGACGATGCGCGTAAAAGCCCCTATTTATAA

>seq2[organism=Giant Panda Escherichia coli] Giant Panda Escherichia coli strain GP003, DNA topoisomerase (ATP-hydrolyzing) subunit B gene.

CATGACGGCACCGGTCTGCACCACATGGTATTCGAGGTGGTAGATAACGCTATCGACGAAGCGCTCGCGGGTCACTGTAAAGAAATTATCGTCACCATTCACGCCGATAACTCTGTCTCTGTACAGGATGACGGGCGCGGCATTCCGACCGGTATTCACCCGGAAGAGGGCGTATCGGCGGCGGAAGTGATCATGACCGTTCTGCACGCAGGCGGTAAATTTGACGATAACTCCTATAAAGTGTCCGGCGGTCTGCACGGCGTTGGTGTTTCGGTAGTAAACGCCCTGTCGCAAAAACTGGAGCTGGTTATCCAGCGCGAGGGTAAAATTCACCGTCAGATCTACGAACACGGTGTACCGCAGGCCCCGCTGGCGGTTACCGGCGAGACTGAAAAAACCGGCACCATGGTGCGTTTCTGGCCCAGCCTCGAAACCTTCACCAATGTGACCGAGTTCGAATATGAAATTCTGGCGAAACGTCTGCGTGAGTTGTCGTTCCTCAACTCCGGCGTTTCCATTCGTCTGCGCGACAAGCGCGACGGCAAAGAAGACCACTTCCACTATGAAGGCGGCATCAAGGCGTTCGTTGAATATCTGAACAAGAACAAAACGCCGATCCACCCGAATATCTTCTACTTCTCCACTGAAAAAGACGGTATTGGCGTCGAAGTGGCGTTGCAGTGGAACGATGGCTTCCAGGAAAACATCTACTGCTTTACCAACAACATTCCGCAGCGTGACGGCGGTACTCACCTGGCAGGCTTCCGTGCGGCGATGACCCGTACCCTGAACGCCTACATGGACAAAGAAGGCTACAGCAAAAAAGCCAAAGTCAGCGCCACCGGTGACGATGCGCGTAGGGGCCCTGAATATAAA

>seq3[organism=Giant Panda Escherichia coli] Giant Panda Escherichia coli strain GP004, DNA topoisomerase (ATP-hydrolyzing) subunit B gene.

CACCATGACGGCACCGGTCTGCACCACATGGTATTCGAGGTGGTAGATAACGCTATCGACGAAGCGCTCGCGGGTCACTGTAAAGAAATTATCGTCACCATTCACGCCGATAACTCTGTCTCTGTACAGGATGACGGGCGCGGCATTCCGACCGGTATTCACCCGGAAGAGGGCGTATCGGCGGCGGAAGTGATCATGACCGTTCTGCACGCAGGCGGTAAATTTGACGATAACTCCTATAAAGTGTCCGGCGGTCTGCACGGCGTTGGTGTTTCGGTAGTAAACGCCCTGTCGCAAAAACTGGAGCTGGTTATCCAGCGCGAAGGTAAAATTCACCGTCAGATCTACGAACACGGTGTACCGCAAGCCCCGCTGGCGGTTACCGGCGAGACTGAAAAAACCGGCACCATGGTGCGTTTCTGGCCCAGCCTCGAAACCTTCACCAATGTGACCGAGTTCGAATATGAAATTCTGGCGAAACGTCTGCGTGAGTTGTCGTTCCTCAACTCCGGCGTTTCCATTCGTCTGCGCGACAAGCGCGACGGCAAAGAAGACCACTTCCACTATGAAGGCGGCATCAAGGCGTTCGTTGAATATCTGAACAAGAACAAAACGCCGATCCACCCGAATATCTTCTACTTCTCCACTGAAAAAGACGGTATTGGCGTCGAAGTGGCGTTGCAGTGGAACGATGGCTTCCAGGAAAACATCTACTGCTTTACCAACAACATTCCGCAGCGTGACGGCGGTACTCACCTGGCAGGCTTCCGTGCGGCGATGACCCGTACCCTGAACGCCTACATGGACAAAGAAGGCTACAGCAAAAAAGCCAAAGTCAGCGCCACCGGTGACGATGCGCGTAAGGGCGCCCTGATTAGA

>seq4[organism=Giant Panda Escherichia coli] Giant Panda Escherichia coli strain GP012, DNA topoisomerase (ATP-hydrolyzing) subunit B gene.

TTCGGGCGACCCCCATGACGGCACCGGTCTGCACCACATGGTATTCGAGGTGGTAGATAACGCTATCGACGAAGCGCTCGCGGGTCACTGTAAAGAAATTATCGTCACCATTCACGCCGATAACTCTGTCTCTGTACAGGATGACGGGCGCGGCATTCCGACCGGTATTCACCCGGAAGAGGGCGTATCGGCGGCGGAAGTGATCATGACCGTTCTGCACGCAGGCGGTAAATTTGACGATAACTCCTATAAAGTGTCCGGCGGTCTGCACGGCGTTGGTGTTTCGGTAGTAAACGCCCTGTCGCAAAAACTGGAGCTGGTTATCCAGCGCGAGGGTAAAATTCACCGTCAGATCTACGAACACGGTGTACCGCAGGCCCCGCTGGCGGTTACCGGCGAGACTGAAAAAACCGGCACCATGGTGCGTTTCTGGCCAAGCCTTGAAACCTTCACCAATGTGACCGAGTTCGAATATGACATTCTGGCGAAACGTCTGCGTGAGTTGTCGTTCCTCAACTCCGGCGTTTCCATTCGTCTGCGCGACAAGCGCGACGGCAAAGAAGACCACTTCCACTATGAAGGCGGCATCAAGGCGTTCGTTGAATATCTGAACAAGAACAAAACGCCGATCCACCCGAATATCTTCTACTTCTCCACCGAAAAAGACGGTATTGGCGTCGAAGTGGCGTTGCAGTGGAACGATGGCTTCCAGGAAAACATCTACTGCTTTACCAACAACATTCCGCAGCGTGATGGCGGTACTCACCTGGCAGGCTTCCGTGCGGCGATGACCCGCACCCTGAACGCCTACATGGACAAAGAAGGCTACAGCAAAAAAGCCAAAGTCAGCGCCACCGGTGACGATGCGCTAAGGGGCTTTAATATA

>seq5[organism=Giant Panda Escherichia coli] Giant Panda Escherichia coli strain GP014, DNA topoisomerase (ATP-hydrolyzing) subunit B gene.

TCGGGCAGAACACCATGACGGCACCGGTCTGCACCACATGGTATTCGAGGTGGTAGATAACGCTATCGACGAAGCGCTCGCGGGTCACTGTAAAGAAATTATCGTCACCATTCACGCCGATAACTCTGTCTCTGTACAGGATGACGGGCGCGGCATTCCGACCGGTATTCACCCGGAAGAGGGCGTATCGGCGGCGGAAGTGATCATGACCGTTCTGCACGCAGGCGGTAAATTTGACGATAACTCCTATAAAGTGTCCGGCGGTCTGCACGGCGTTGGTGTTTCGGTAGTAAACGCCCTGTCGCAAAAACTGGAGCTGGTTATCCAGCGCGAGGGTAAAATTCACCGTCAGATCTACGAACACGGTGTACCGCAGGCTCCGCTGGCGGTTACCGGCGAGACTGAAAAAACCGGCACCATGGTGCGTTTCTGGCCCAGCCTCGAAACCTTCACCAATGTGACCGAGTTCGAATATGAAATTCTGGCGAAACGTCTGCGTGAGTTGTCGTTCCTCAACTCCGGCGTTTCCATTCGTCTGCGCGACAAGCGCGACGGCAAAGAAGACCACTTCCACTATGAAGGCGGCATCAAGGCGTTCGTTGAATATCTGAACAAGAACAAAACGCCGATCCACCCGAATATCTTCTACTTCTCCACTGAAAAAGACGGTATTGGCGTCGAAGTGGCGTTGCAGTGGAACGATGGCTTCCAGGAAAACATCTACTGCTTTACCAACAACATTCCGCAGCGTGACGGCGGTACTCACCTGGCAGGCTTCCGTGCGGCGATGACCCGTACCCTGAACGCCTACATGGACAAAGAAGGCTACAGCAAAAAAGCCAAAGTCAGCGCCACCGGTGACGATGCGCGTAGGGCGCCTTGAAATAAAA

>seq6[organism=Giant Panda Escherichia coli] Giant Panda Escherichia coli strain GP022, DNA topoisomerase (ATP-hydrolyzing) subunit B gene.

CTCGGGGGAAAAACCATGACGGCACCGGTCTGCACCACATGGTATTCGAGGTGGTAGATAACGCTATCGACGAAGCGCTCGCGGGTCACTGTAAAGAAATTATCGTCACCATTCACGCCGATAACTCTGTCTCTGTACAGGATGACGGGCGCGGTATTCCGACCGGTATTCACCCGGAAGAGGGCGTATCGGCGGCGGAAGTGATCATGACCGTTCTGCACGCAGGCGGTAAATTTGACGATAACTCCTATAAAGTGTCCGGCGGTCTGCACGGCGTTGGTGTTTCGGTAGTAAACGCCCTGTCGCAAAAACTGGAGCTGGTTATCCAGCGCGAGGGTAAAATTCACCGTCAGATCTACGAACACGGTGTACCGCAGGCTCCGCTGGCGGTTACCGGCGAGACTGAAAAAACCGGCACCATGGTGCGTTTCTGGCCCAGCCTCGAAACCTTCACCAATGTGACCGAGTTCGAATATGAAATTCTGGCGAAACGTCTGCGTGAGTTGTCGTTCCTCAACTCCGGCGTTTCCATTCGTCTGCGCGATAAGCGCGACGGCAAAGAAGACCACTTCCACTATGAAGGCGGCATCAAGGCGTTCGTTGAATATCTGAACAAGAACAAAACGCCGATCCACCCGAATATCTTCTACTTCTCCACTGAAAAAGACGGTATTGGCGTCGAAGTGGCGTTGCAGTGGAACGATGGCTTCCAGGAAAACATCTACTGCTTTACCAACAACATTCCGCAGCGTGACGGCGGTACTCACCTGGCAGGCTTCCGTGCGGCGATGACCCGTACCCTGAACGCCTACATGGACAAAGAAGGCTACAGCAAAAAAGCCAAAGTCAGCGCCACCGGTGACGATGCGCGTAAAGGCCCTGAATATAA

>seq7[organism=Giant Panda Escherichia coli] Giant Panda Escherichia coli strain GP030, DNA topoisomerase (ATP-hydrolyzing) subunit B gene.

CATGACGGCACCGGTCTGCACCACATGGTATTCGAGGTGGTAGATAACGCTATCGACGAAGCGCTCGCGGGTCACTGTAAAGAAATTATCGTCACCATTCACGCCGATAACTCTGTCTCTGTACAGGATGACGGGCGCGGCATTCCGACCGGTATTCACCCGGAAGAGGGCGTATCGGCGGCGGAAGTGATCATGACCGTTCTGCACGCAGGCGGTAAATTTGACGATAACTCCTATAAAGTGTCCGGCGGTCTGCACGGCGTTGGTGTTTCGGTAGTAAACGCCCTGTCGCAAAAACTGGAGCTGGTTATCCAGCGCGAGGGTAAAATTCACCGTCAGATCTACGAACACGGTGTACCGCAGGCCCCGCTGGCGGTTACCGGCGAGACTGAAAAAACCGGCACCATGGTGCGTTTCTGGCCCAGCCTCGAAACCTTCACCAATGTGACCGAGTTCGAATATGAAATTCTGGCGAAACGTCTGCGTGAGTTGTCGTTCCTCAACTCCGGCGTTTCCATTCGTCTGCGCGACAAGCGCGACGGCAAAGAAGACCACTTCCACTATGAAGGCGGCATCAAGGCGTTCGTTGAATATCTGAACAAGAACAAAACGCCGATCCACCCGAATATCTTCTACTTCTCCACTGAAAAAGACGGTATTGGCGTCGAAGTGGCGTTGCAGTGGAACGATGGCTTCCAGGAAAACATCTACTGCTTTACCAACAACATTCCGCAGCGTGACGGCGGTACTCACCTGGCAGGCTTCCGTGCGGCGATGACCCGTACCCTGAACGCCTACATGGACAAAGAAGGCTACAGCAAAAAAAGCCAAAGTCAGCGCCACCGGTGACGATGCGCGTAAGAGGGCCCTGTAAATAA

>seq8[organism=Giant Panda Escherichia coli] Giant Panda Escherichia coli strain GP032, DNA topoisomerase (ATP-hydrolyzing) subunit B gene.

CAGACGGCACCGGTCTGCACCACATGGTATTCGAGGTGGTAGATAACGCTATCGACGAAGCGCTCGCGGGTCACTGTAAAGAAATTATCGTCACCATTCACGCCGATAACTCTGTCTCTGTACAGGATGACGGGCGCGGCATTCCGACCGGTATTCACCCGGAAGAGGGCGTATCGGCGGCGGAAGTGATCATGACCGTTCTGCACGCAGGCGGTAAATTTGACGATAACTCCTATAAAGTGTCCGGCGGTCTGCACGGCGTTGGTGTTTCGGTAGTAAACGCCCTGTCGCAAAAACTGGAGCTGGTTATCCAGCGCGAGGGTAAAATTCACCGTCAGATCTACGAACACGGTGTACCGCAGGCCCCGCTGGCGGTTACCGGCGAGACTGAAAAAACCGGCACCATGGTGCGTTTCTGGCCAAGCCTTGAAACCTTCACCAATGTGACCGAGTTCGAATATGACATTCTGGCGAAACGTCTGCGTGAGTTGTCGTTCCTCAACTCCGGCGTTTCCATTCGTCTGCGCGATAAGCGCGACGGCAAAGAAGACCACTTCCACTATGAAGGCGGCATCAAGGCGTTCGTTGAATATCTGAACAAGAACAAAACGCCGATCCACCCGAATATCTTCTACTTCTCCACTGAAAAAGACGGTATTGGCGTCGAAGTGGCGTTGCAGTGGAACGATGGCTTCCAGGAAAACATCTACTGCTTTACCAACAACATTCCGCAGCGTGACGGCGGTACTCACCTGGCAGGCTTCCGTGCGGCGATGACCCGTACCCTGAACGCCTACATGGACAAAGAAGGCTACAGCAAAAAAGCCAAAGTCAGCGCCACCGGTGACGATGCGCGTAAAAGGCCCTGTTATAAAAA

>seq9[organism=Giant Panda Escherichia coli] Giant Panda Escherichia coli strain GP050, DNA topoisomerase (ATP-hydrolyzing) subunit B gene.

CAGACGGCACCGGTCTGCACCACATGGTATTCGAGGTGGTAGATAACGCTATCGACGAAGCGCTCGCGGGTCACTGTAAAGAAATTATCGTCACCATTCACGCCGATAACTCTGTCTCTGTACAGGATGACGGGCGCGGCATTCCGACCGGTATTCACCCGGAAGAGGGCGTATCGGCGGCGGAAGTGATCATGACCGTTCTGCACGCAGGCGGTAAATTTGACGATAACTCCTATAAAGTGTCCGGCGGTCTGCACGGCGTTGGTGTTTCGGTAGTAAACGCCCTGTCGCAAAAACTGGAGCTGGTTATCCAGCGCGAGGGTAAAATTCACCGTCAGATCTACGAACACGGTGTACCGCAGGCCCCGCTGGCGGTTACCGGCGAGACTGAAAAAACCGGCACTATGGTGCGTTTCTGGCCAAGCCTTGAAACCTTCACCAATGTGACCGAGTTCGAATATGACATTCTGGCGAAACGTCTGCGTGAGTTGTCGTTCCTCAACTCCGGCGTTTCCATTCGTCTGCGCGACAAGCGCGACGGCAAAGAAGACCACTTCCACTATGAAGGCGGCATCAAGGCGTTCGTTGAATATCTGAACAAGAACAAAACGCCGATCCACCCGAATATCTTCTACTTCTCCACCGAAAAAGACGGTATTGGCGTCGAAGTGGCGTTGCAGTGGAACGATGGCTTCCAGGAAAACATCTACTGCTTTACCAACAACATTCCGCAGCGTGACGGCGGTACTCACCTGGCAGGTTTCCGTGCGGCGATGACCCGTACTCTGAACGCCTACATGGACAAAGAAGGCTACAGCAAAAAAGCCAAAGTCAGCGCCACCGGTGACGATGCGCGTAGAGGGCCCTAAATATAAA

>seq10[organism=Giant Panda Escherichia coli] Giant Panda Escherichia coli strain GP065, DNA topoisomerase (ATP-hydrolyzing) subunit B gene.

TTCGGGCGAACAACGCATGACGGCACCGGTCTGCACCACATGGTATTCGAGGTGGTAGATAACGCTATCGACGAAGCGCTCGCGGGTCACTGTAAAGAAATTATCGTCACCATTCACGCCGATAACTCTGTCTCTGTACAGGATGACGGGCGCGGCATTCCGACCGGTATTCACCCGGAAGAGGGCGTATCGGCGGCGGAAGTGATCATGACCGTTCTGCACGCAGGCGGTAAATTTGACGATAACTCCTATAAAGTGTCCGGCGGTCTGCACGGCGTTGGTGTTTCGGTAGTAAACGCCCTGTCGCAAAAACTGGAGCTGGTTATCCAGCGCGAGGGTAAAATTCACCGTCAGATCTACGAACACGGTGTACCGCAGGCCCCGCTGGCGGTTACCGGCGAGACTGAAAAAACCGGCACCATGGTGCGTTTCTGGCCCAGCCTCGAAACCTTCACCAATGTGACCGAGTTCGAATATGAAATTCTGGCGAAACGTCTGCGTGAGTTGTCGTTCCTCAACTCCGGCGTTTCCATTCGTCTGCGCGACAAGCGCGACGGCAAAGAAGACCACTTCCACTATGAAGGCGGCATCAAGGCGTTCGTTGAATATCTGAACAAGAACAAAACGCCGATCCACCCGAATATCTTCTACTTCTCCACTGAAAAAGACGGTATTGGCGTCGAAGTGGCGTTGCAGTGGAACGATGGCTTCCAGGAAAACATCTACTGCTTTACCAACAACATTCCGCAGCGTGACGGCGGTACTCACCTGGCAGGCTTCCGTGCGGCGATGACCCGTACCCTGAACGCCTACATGGACAAAGAAGGCTACAGCAAAAAAGCCAAAGTCAGCGCCACCGGTGACGATGCGCGGAAGGGGCTTGATTAA

>seq11[organism=Giant Panda Escherichia coli] Giant Panda Escherichia coli strain GP095, DNA topoisomerase (ATP-hydrolyzing) subunit B gene.

ACACAGACGGCACCGGTCTGCACCACATGGTATTCGAGGTGGTAGATAACGCTATCGACGAAGCGCTCGCGGGTCACTGTAAAGAAATTATCGTCACCATTCACGCCGATAACTCTGTCTCTGTACAGGATGACGGGCGCGGCATTCCGACCGGTATTCACCCGGAAGAGGGCGTATCGGCGGCGGAAGTGATCATGACCGTTCTGCACGCAGGCGGTAAATTTGACGATAACTCCTATAAAGTGTCCGGCGGTCTGCACGGCGTTGGTGTTTCGGTAGTAAACGCCCTGTCGCAAAAACTGGAGCTGGTTATCCAGCGCGAGGGTAAAATTCACCGTCAGATCTACGAACACGGTGTACCGCAGGCTCCGCTGGCGGTTACCGGCGAGACTGAAAAAACAGGCACCATGGTGCGTTTCTGGCCCAGCCTCGAAACCTTCACCAATGTGACCGAGTTCGAATATGAAATTCTGGCGAAACGTCTGCGTGAGTTGTCGTTCCTCAACTCCGGCGTTTCCATTCGTCTGCGCGATAAGCGCGACGGCAAAGAAGACCACTTCCACTATGAAGGCGGCATCAAGGCGTTCGTTGAATATCTGAACAAGAACAAAACGCCGATCCACCCGAATATCTTCTACTTCTCCACTGAAAAAGACGGTATTGGCGTCGAAGTGGCGTTGCAGTGGAACGATGGCTTCCAGGAAAACATCTACTGCTTTACCAACAACATTCCGCAGCGTGACGGCGGTACTCACCTGGCAGGCTTCCGTGCGGCGATGACCCGTACCCTGAACGCCTACATGGACAAAGAAGGCTACAGCAAAAAAGCCAAAGTCAGCGCCACCGGTGACGATGCGCGGAAGGGGGCTGATATAAA

>seq12[organism=Giant Panda Escherichia coli] Giant Panda Escherichia coli strain GP101, DNA topoisomerase (ATP-hydrolyzing) subunit B gene.

ACCATGACGGCACCGGTCTGCACCACATGGTATTCGAGGTGGTAGATAACGCTATCGACGAAGCGCTCGCGGGTCACTGTAAAGAAATTATCGTCACCATTCATGCCGACAACTCTGTCTCTGTACAGGACGACGGGCGCGGCATTCCGACCGGTATTCACCCGGAAGAGGGCGTATCGGCGGCGGAAGTGATCATGACCGTTCTGCACGCAGGCGGTAAATTCGACGATAACTCCTATAAAGTGTCCGGCGGTCTGCACGGCGTTGGTGTTTCGGTAGTAAACGCCCTGTCGCAAAAACTGGAGCTGGTTATCCAGCGCGAGGGTAAAATTCACCGTCAGATCTACGAACACGGTGTACCGCAGGCCCCGCTGGCGGTTACCGGCGAGACTGAAAAAACCGGCACCATGGTGCGTTTCTGGCCTAGCCTCGAAACTTTCACCAATGTGACCGAGTTCGAATATGAAATTCTGGCGAAACGTCTGCGTGAGTTGTCGTTCCTCAACTCCGGCGTTTCCATTCGTCTGCGCGACAAGCGCGACGGCAAAGAAGACCACTTCCACTATGAAGGCGGCATCAAGGCGTTCGTTGAATATCTGAACAAGAACAAAACGCCGATCCACCCGAATATCTTCTACTTCTCCACTGAAAAAGACGGTATTGGCGTCGAAGTGGCGTTGCAGTGGAACGATGGCTTCCAGGAAAACATCTACTGCTTTACCAACAACATTCCGCAGCGTGACGGCGGTACTCACCTGGCAGGCTTCCGTGCGGCGATGACCCGTACCCTGAACGCCTACATGGACAAAGAAGGCTACAGCAAAAAAGCCAAAGTTAGCGCCACCGGTGACGATGCGCGTAAAGGCCCCTGTATATAAA
