# Supplementary material for: Antibiotic-resistant characteristics and horizontal gene transfer ability analysis of extended-spectrum β-lactamase-producing Escherichia coli isolated from giant pandas
Source: Front Vet Sci. 2024 Jul 26;11:1394814. doi: 10.3389/fvets.2024.1394814 (PMC11310934; doi:10.3389/fvets.2024.1394814)
Supplement: Supplementary file 4 [file Data_Sheet_4.docx]

>seq1[organism=Giant Panda Escherichia coli] Giant Panda Escherichia coli strain GP001, NADP-dependent isocitrate dehydrogenase gene.

TTAAAGAAAAAAAAAATGTTCCGGCACAAGGCAAGAAGATCACCCTGCAAAACGGCAAACTCAACGTTCCTGAAAATCCGATTATCCCTTACATTGAAGGTGATGGAATCGGTGTAGATGTAACCCCAGCCATGCTGAAAGTGGTCGACGCTGCAGTCGAGAAAGCCTATAAAGGCGAGCGTAAAATCTCCTGGATGGAAATTTACACCGGTGAAAAATCCACACAGGTTTATGGTCAGGACGTCTGGTTGCCTGCTGAAACCCTTGATCTGATTCGTGAATATCGCGTTGCCATTAAAGGCCCGCTGACCACTCCGGTTGGTGGCGGTATTCGCTCTCTGAACGTTGCCCTGCGCCAGGAACTGGATCTCTACATCTGCCTGCGTCCGGTACGTTACTATCAGGGCACTCCAAGCCCGGTTAAACACCCTGAACTGACCGATATGGTTATCTTCCGTGAAAACTCGGAAGACATTTATGCGGGTATCGAATGGAAAGCTGACTCTGCCGACGCCGAGAAAGTGATTAAATTCCTGCGTGAAGAGATGGGCGTGAAGAAAATTCGCTTCCCGGAACATTGCGGCATCGGTATTAAGCCGTGTTCTGAAGAAGGCACCAAACGTCTGGTTCGTGCAGCGATCGAATACGCAATTGCTAACGATCGTGACTCTGTGACCCTGGTGCACAAAGGCAACATCATGAAGTTCACCGAAGGCGCGTTTAAAGACTGGGGCTACCAGCTGGCGCGTGAAGAATTTGGCGGTGAACTGATCGACGGCGGCCCGTGGCTGAAAGTTAAAAACCCGAATACCGGCAAAGAGATCGTCATAAAGATAGGATGACAG

>seq2[organism=Giant Panda Escherichia coli] Giant Panda Escherichia coli strain GP003, NADP-dependent isocitrate dehydrogenase gene.

TAGTGTTCCGGCACAAGGCAAGAAGATCACCCTGCAAAACGGCAAACTCAACGTTCCTGATAATCCGATTATCCCTTACATTGAAGGTGATGGAATCGGTGTAGATGTAACCCCAGCCATGCTGAAAGTGGTCGACGCTGCAGTCGAGAAAGCCTATAAAGGCGAGCGTAAAATCTCCTGGATGGAAATTTACACCGGTGAAAAATCCACACAGGTTTATGGTCAGGACGTCTGGCTGCCTGCTGAAACTCTTGATCTGATTCGTGAATATCGCGTTGCCATTAAAGGTCCGCTGACCACTCCGGTTGGTGGCGGTATTCGCTCTCTGAACGTTGCCCTGCGCCAGGAACTGGATCTCTACATCTGCCTGCGTCCGGTACGTTACTATCAGGGCACTCCAAGCCCGGTTAAACACCCTGAACTGACCGATATGGTTATCTTCCGTGAAAACTCGGAAGACATTTATGCGGGTATCGAATGGAAAGCAGACTCTGCCGACGCCGAGAAAGTGATTAAATTCCTGCGTGAAGAGATGGGGGTGAAGAAAATTCGCTTCCCGGAACATTGTGGTATCGGTATTAAGCCGTGTTCGGAAGAAGGCACCAAACGTCTGGTTCGTGCAGCGATCGAATACGCAATTGCTAACGATCGTGACTCTGTGACTCTGGTGCACAAAGGCAACATCATGAAGTTCACCGAAGGAGCGTTTAAAGACTGGGGCTACCAGCTGGCGCGTGAAGAGTTTGGCGGTGAACTGATCGACGGTGGCCCGTGGCTGAAAGTTAAAAACCCGAACACTGGCAAAGAGATCGTCATAAAGACGGATAAGCCAC

>seq3[organism=Giant Panda Escherichia coli] Giant Panda Escherichia coli strain GP004, NADP-dependent isocitrate dehydrogenase gene.

TTTTTTGTGTTGGGGTTTGTTTGATGTTCCGGCACAAGGCAAGAAGATCACCCTGCAAAACGGCAAACTCAACGTTCCTGAAAATCCGATTATCCCTTACATTGAAGGTGATGGAATCGGTGTAGATGTAACCCCAGCCATGCTGAAAGTGGTCGACGCTGCAGTCGAGAAAGCCTATAAAGGCGAGCGTAAAATCTCCTGGATGGAAATTTACACCGGTGAAAAATCCACACAGGTTTATGGTCAGGACGTCTGGCTGCCTGCTGAAACTCTTGATCTGATTCGTGAATATCGCGTTGCCATTAAAGGTCCGCTGACCACTCCGGTTGGTGGCGGTATTCGCTCTCTGAACGTTGCCCTGCGCCAGGAACTGGATCTCTACATCTGCCTGCGTCCGGTACGTTACTATCAGGGCACTCCAAGCCCGGTTAAACACCCTGAACTGACCGATATGGTTATCTTCCGTGAAAACTCGGAAGACATTTATGCGGGTATCGAATGGAAAGCAGACTCTGCCGACGCCGAGAAAGTGATTAAATTCCTGCGTGAAGAGATGGGGGTGAAGAAAATTCGCTTCCCGGAACATTGTGGTATCGGTATTAAGCCGTGTTCGGAAGAAGGCACCAAACGTCTGGTTCGTGCAGCGATCGAATACGCAATTGCTAACGATCGTGACTCTGTGACTCTGGTGCACAAAGGCAACATCATGAAGTTCACCGAAGGAGCGTTTAAAGACTGGGGCTACCAGCTGGCGCGTGAAGAGTTTGGCGGTGAACTGATCGACGGTGGCCCGTGGCTGAAAGTTAAAAACCCGAACACTGGCAAAGAGATCGTCATAAAGACGGATAAGCC

>seq4[organism=Giant Panda Escherichia coli] Giant Panda Escherichia coli strain GP012, NADP-dependent isocitrate dehydrogenase gene.

AGTGTTCCGGCACAAGGCAAGAAGATCACCCTGCAAAACGGCAAACTCAACGTTCCTGAAAATCCGATTATCCCTTACATTGAAGGTGATGGAATCGGTGTAGATGTAACCCCAGCCATGCTGAAAGTGGTCGACGCTGCAGTCGAGAAAGCCTATAAAGGCGAGCGTAAAATCTCCTGGATGGAAATTTACACCGGTGAAAAATCCACACAGGTTTATGGTCAGGACGTCTGGTTGCCTGCTGAAACCCTTGATCTGATTCGTGAATATCGCGTTGCCATTAAAGGTCCGCTGACCACTCCGGTTGGTGGCGGTATTCGCTCTCTGAACGTTGCCCTGCGCCAGGAACTGGATCTCTACATCTGCCTGCGTCCGGTACGTTACTATCAGGGCACTCCAAGCCCGGTTAAACACCCTGAACTGACCGATATGGTTATCTTCCGTGAAAACTCGGAAGACATTTATGCGGGTATCGAATGGAAAGCAGACTCTGCCGACGCTGAGAAAGTGATTAAATTCCTGCGTGAAGAGATGGGCGTGAAGAAAATTCGCTTCCCGGAACATTGCGGTATCGGTATTAAGCCGTGTTCGGAAGAAGGCACCAAACGTCTGGTTCGTGCAGCGATCGAATACGCAATTGCTAACGATCGTGACTCTGTGACTCTGGTGCACAAAGGCAACATCATGAAGTTCACCGAAGGCGCGTTTAAAGACTGGGGCTACCAACTGGCGCGTGAAGAATTTGGCGGTGAACTGATCGACGGCGGCCCGTGGCTGAAAGTTAAAAACCCGAACACCGGCAAAGAGATCGTCATAAAGACGTATGATCAAG

>seq5[organism=Giant Panda Escherichia coli] Giant Panda Escherichia coli strain GP014, NADP-dependent isocitrate dehydrogenase gene.

ATAAAAGAAAAAGAGTTAGTAGTTGTTCCGGCACAAGGCAAGAAGATCACCCTGCAAAACGGCAAACTCAACGTTCCTGAAAATCCGATTATCCCTTACATTGAAGGTGATGGAATCGGTGTAGATGTAACCCCAGCCATGCTGAAAGTGGTCGACGCTGCAGTCGAGAAAGCCTATAAAGGCGAGCGTAAAATCTCCTGGATGGAAATTTACACCGGTGAAAAATCCACACAGGTTTATGGTCAGGATGTCTGGCTGCCTGCTGAAACCCTTGATCTGATTCGTGAATATCGCGTTGCCATTAAAGGTCCGCTGACCACTCCTGTTGGTGGCGGTATTCGCTCTCTGAACGTTGCCCTGCGCCAGGAACTGGATCTCTACATCTGCCTGCGTCCGGTACGTTACTATCAGGGCACTCCAAGCCCGGTTAAACACCCTGAACTGACCGATATGGTTATCTTCCGTGAAAACTCGGAAGACATTTATGCGGGTATCGAATGGAAAGCAGACTCTGCCGACGCCGAGAAAGTGATTAAATTCCTGCGTGAAGAGATGGGGGTGAAGAAAATTCGCTTCCCGGAACATTGTGGTATCGGTATTAAGCCGTGTTCGGAAGAAGGCACCAAACGTCTGGTTCGTGCAGCGATCGAATACGCAATTGCTAACGATCGTGACTCTGTGACTCTGGTGCACAAAGGCAACATCATGAAGTTCACCGAAGGAGCGTTTAAAGACTGGGGCTACCAGCTGGCGCGTGAAGAGTTTGGCGGTGAACTGATCGACGGTGGCCCGTGGCTGAAAGTTAAAAACCCGAACACTGGCAAAAGAGATCGTCATTAAAGACGTGATCGCTGATGCATTCCTGCAACAGATCCGGCTGCGGTCCCA

>seq6[organism=Giant Panda Escherichia coli] Giant Panda Escherichia coli strain GP022, NADP-dependent isocitrate dehydrogenase gene.

TAGTGTTCCGGCACAAGGCAAGAAGATCACCCTGCAAAACGGCAAACTCAACGTTCCTGAAAATCCGATTATCCCTTACATTGAAGGTGATGGAATCGGTGTAGATGTAACCCCAGCCATGCTGAAAGTGGTCGACGCTGCAGTCGAGAAAGCCTATAAAGGCGAGCGTAAAATCTCCTGGATGGAAATTTACACCGGTGAAAAATCCACACAGGTTTATGGTCAGGATGTCTGGCTACCTGCTGAAACCCTTGATCTGATTCGTGAATATCGCGTTGCCATCAAAGGCCCGCTGACCACTCCGGTTGGTGGTGGTATTCGCTCTCTCAACGTTGCTCTGCGCCAGGAACTGGATCTCTACATCTGCCTGCGTCCGGTACGTTACTATCAGGGCACTCCAAGCCCGGTTAAACACCCTGAACTGACCGATATGGTTATCTTCCGTGAAAACTCGGAAGACATTTATGCGGGTATCGAATGGAAAGCAGACTCTGCCGACGCTGAGAAAGTGATTAAATTCCTGCGTGAAGAGATGGGCGTGAAGAAAATTCGCTTCCCGGAACATTGCGGCATCGGTATTAAGCCGTGTTCTGAAGAAGGCACCAAACGTCTGGTTCGTGCAGCGATCGAATACGCAATTGCTAACGATCGTGACTCTGTGACCCTGGTGCACAAAGGCAACATCATGAAGTTCACCGAAGGCGCGTTTAAAGACTGGGGCTACCAGCTGGCGCGTGAAGAGTTTGGCGGTGAACTGATCGACGGCGGCCCGTGGCTGAAAGTTAAAAACCCGAACACCGGCAAAGAGATCGTCATTAAAGACGTGATTGCTGATGCATTCCTGCAACAGATCCTGACTGGGCT

>seq7[organism=Giant Panda Escherichia coli] Giant Panda Escherichia coli strain GP030, NADP-dependent isocitrate dehydrogenase gene.

TAGTGTTCCGGCACAAGGCAAGAAGATCACCCTGCAAAACGGCAAACTCAACGTTCCTGAAAATCCGATTATCCCTTACATTGAAGGTGATGGAATCGGTGTAGATGTAACCCCAGCCATGCTGAAAGTGGTCGACGCTGCAGTCGAGAAAGCCTATAAAGGCGAGCGTAAAATCTCCTGGATGGAAATTTACACCGGTGAAAAATCCACACAGGTTTATGGTCAGGACGTCTGGCTGCCTGCTGAAACTCTTGATCTGATTCGTGAATATCGCGTTGCCATTAAAGGTCCGCTGACCACTCCGGTTGGTGGCGGTATTCGCTCTCTGAACGTTGCCCTGCGCCAGGAACTGGATCTCTACATCTGCCTGCGTCCGGTACGTTACTATCAGGGCACTCCAAGCCCGGTTAAACACCCTGAACTGACCGATATGGTTATCTTCCGTGAAAACTCGGAAGACATTTATGCGGGTATCGAATGGAAAGCAGACTCTGCCGACGCCGAGAAAGTGATTAAATTCCTGCGTGAAGAGATGGGGGTGAAGAAAATTCGCTTCCCGGAACATTGTGGTATCGGTATTAAGCCGTGTTCGGAAGAAGGCACCAAACGTCTGGTTCGTGCAGCGATCGAATACGCAATTGCTAACGATCGTGACTCTGTGACTCTGGTGCACAAAGGCAACATCATGAAGTTCACCGAAGGAGCGTTTAAAGACTGGGGCTACCAGCTGGCGCGTGAAGAGTTTGGCGGTGAACTGATCGACGGTGGCCCGTGGCTGAAAGTTAAAAACCCGAACACTGGCAAAGAGATCGTCATTAAAGACGTGATTGCTGATGCATTCCTGCAACAGATCTGCCCTGCGTCCAA

>seq8[organism=Giant Panda Escherichia coli] Giant Panda Escherichia coli strain GP032, NADP-dependent isocitrate dehydrogenase gene.

AGTGTTCCGGCACAAGGCAAGAAGATCACCCTGCAAAACGGCAAACTCAACGTTCCTGAAAATCCGATTATCCCTTACATTGAAGGTGATGGAATCGGTGTAGATGTAACCCCAGCCATGCTGAAAGTGGTCGACGCTGCAGTCGAGAAAGCCTATAAAGGCGAGCGTAAAATCTCCTGGATGGAAATTTACACCGGTGAAAAATCCACACAGGTTTATGGTCAGGATGTCTGGCTACCTGCTGAAACCCTTGATCTGATTCGTGAATATCGCGTTGCCATCAAAGGCCCGCTGACCACTCCGGTTGGTGGCGGTATTCGCTCTCTCAACGTTGCTCTGCGCCAGGAACTGGATCTCTACATCTGCCTGCGTCCGGTACGTTACTATCAGGGCACTCCAAGCCCGGTTAAACACCCTGAACTGACCGATATGGTTATCTTCCGTGAAAACTCGGAAGACATTTATGCGGGTATCGAATGGAAAGCAGACTCTGCCGACGCTGAGAAAGTGATTAAATTCCTGCGTGAAGAGATGGGCGTGAAGAAAATTCGCTTCCCGGAACATTGCGGTATCGGTATTAAGCCGTGTTCTGAAGAAGGCACCAAACGTCTGGTTCGTGCAGCGATCGAATACGCAATTGCTAACGATCGTGACTCTGTGACCCTGGTGCACAAAGGCAACATCATGAAGTTCACCGAAGGCGCGTTTAAAGACTGGGGCTACCAGCTGGCGCGTGAAGAGTTTGGCGGTGAACTGATCGACGGCGGCCCGTGGCTGAAAGTTAAAAACCCGAACACCGGCAAAGAGATCGTCATAAAGACGGATGAGCCA

>seq9[organism=Giant Panda Escherichia coli] Giant Panda Escherichia coli strain GP050, NADP-dependent isocitrate dehydrogenase gene.

TAAGGAAAAAAGTGTTAGTAGTTGTTCCGGCACAAGGCAAGAAGATCACCCTGCAAAACGGCAAACTCAACGTTCCTGAAAATCCGATTATCCCTTACATTGAAGGTGATGGAATCGGTGTAGATGTAACCCCAGCCATGCTGAAAGTGGTCGACGCTGCAGTTGAGAAAGCCTATAAAGGCGAGCGTAAAATCTCCTGGATGGAAATTTACACCGGTGAAAAATCCACACAGGTTTATGGTCAGGACGTCTGGTTGCCTGCTGAAACCCTTGATCTGATTCGTGAATATCGCGTTGCCATTAAAGGCCCGCTGACCACTCCGGTTGGTGGCGGTATTCGCTCTCTGAACGTTGCCCTGCGCCAGGAACTGGATCTCTACATCTGCCTGCGTCCGGTACGTTACTATCAGGGCACTCCAAGCCCGGTTAAACACCCTGAACTGACCGATATGGTTATCTTCCGTGAAAACTCGGAAGACATTTATGCGGGTATCGAATGGAAAGCAGACTCTGCCGACGCCGAGAAAGTGATTAAATTCCTGCGTGAAGAGATGGGCGTGAAGAAAATTCGTTTCCCGGAACATTGCGGTATCGGTATTAAGCCGTGTTCTGAAGAAGGCACCAAACGTCTGGTGCGTGCAGCGATCGAATACGCAATTGCGAACGATCGTGACTCTGTGACCCTGGTGCACAAAGGCAACATCATGAAGTTCACCGAAGGCGCGTTTAAAGACTGGGGCTACCAGCTGGCGCGTGAAGAGTTTGGCGGTGAACTGATCGACGGCGGCCCGTGGCTGAAAGTTAAAAACCCGAATACCGGCAAAGAGATCGTCATTAAAGATGTGATTGCTGATGCATTCCTGCAACAGATCCTGGCTGCATTCCA

>seq10[organism=Giant Panda Escherichia coli] Giant Panda Escherichia coli strain GP065, NADP-dependent isocitrate dehydrogenase gene.

TAAGTAGGAGAAAATGTTATGTAGTTGTTCCGGCACAAGGCAAGAAGATCACCCTGCAAAACGGCAAACTCAACGTTCCTGATAATCCGATTATCCCTTACATTGAAGGTGATGGAATCGGTGTAGATGTAACCCCAGCCATGCTGAAAGTGGTCGACGCTGCAGTCGAGAAAGCCTATAAAGGCGAGCGTAAAATCTCCTGGATGGAAATTTACACCGGTGAAAAATCCACACAGGTTTATGGTCAGGACGTCTGGCTGCCTGCTGAAACTCTTGATCTGATTCGTGAATATCGCGTTGCCATTAAAGGTCCGCTGACCACTCCGGTTGGTGGCGGTATTCGCTCTCTGAACGTTGCCCTGCGCCAGGAACTGGATCTCTACATCTGCCTGCGTCCGGTACGTTACTATCAGGGCACTCCAAGCCCGGTTAAACACCCTGAACTGACCGATATGGTTATCTTCCGTGAAAACTCGGAAGACATTTATGCGGGTATCGAATGGAAAGCAGACTCTGCCGACGCCGAGAAAGTGATTAAATTCCTGCGTGAAGAGATGGGGGTGAAGAAAATTCGCTTCCCGGAACATTGTGGTATCGGTATTAAGCCGTGTTCGGAAGAAGGCACCAAACGTCTGGTTCGTGCAGCGATCGAATACGCAATTGCTAACGATCGTGACTCTGTGACTCTGGTGCACAAAGGCAACATCATGAAGTTCACCGAAGGAGCGTTTAAAGACTGGGGCTACCAGCTGGCGCGTGAAGAGTTTGGCGGTGAACTGATCGACGGTGGCCCGTGGCTGAAAGTTAAAAACCCGAACACTGGCAAAGAGATCGTCATAAAGACGGATGACG

>seq11[organism=Giant Panda Escherichia coli] Giant Panda Escherichia coli strain GP095, NADP-dependent isocitrate dehydrogenase gene.

TAGTTGTTCCGGCACAAGGCAAGAAGATCACCCTGCAAAACGGCAAACTCAACGTTCCTGAAAATCCGATTATCCCTTACATTGAAGGTGATGGAATCGGTGTAGATGTAACCCCAGCCATGCTGAAAGTGGTCGACGCTGCAGTCGAGAAAGCCTATAAAGGCGAGCGTAAAATCTCCTGGATGGAAATTTACACCGGTGAAAAATCCACACAGGTTTATGGTCAGGATGTCTGGCTACCTGCTGAAACCCTTGATCTGATTCGTGAATATCGCGTTGCCATCAAAGGCCCGCTGACCACTCCGGTTGGTGGCGGTATTCGCTCTCTCAACGTTGCTCTGCGCCAGGAACTGGATCTCTACATCTGCCTGCGTCCGGTACGTTACTATCAGGGCACTCCAAGCCCGGTTAAACACCCTGAACTGACCGATATGGTTATCTTCCGTGAAAACTCGGAAGACATTTATGCGGGTATCGAATGGAAAGCAGACTCTGCCGACGCTGAGAAAGTGATTAAATTCCTGCGTGAAGAGATGGGCGTGAAGAAAATTCGCTTCCCGGAACATTGCGGTATCGGTATTAAGCCGTGTTCTGAAGAAGGCACCAAACGTCTGGTTCGTGCAGCGATCGAATACGCAATTGCTAACGATCGTGACTCTGTGACCCTGGTGCACAAAGGCAACATCATGAAGTTCACCGAAGGCGCGTTTAAAGACTGGGGCTACCAGCTGGCGCGTGAAGAGTTTGGCGGTGAACTGATCGACGGCGGCCCGTGGCTGAAAGTTAAAAACCCGAACACCGGCAAAGAGATCGTCATTAAAGACGTGATTGCTGATGCATTCCTGCAACAGATCTGCCCCTGGGGTCCA

>seq12[organism=Giant Panda Escherichia coli] Giant Panda Escherichia coli strain GP101, NADP-dependent isocitrate dehydrogenase gene.

CTAGTGAGGAGAAAAAGTTATAGTTGTTCCGGCACAAGGCAAGAAGATCACCCTGCAAAACGGCAAACTCAACGTTCCTGAAAATCCGATTATCCCTTACATTGAAGGTGATGGAATCGGTGTAGATGTAACCCCAGCCATGCTGAAAGTGGTCGACGCTGCAGTCGAGAAAGCCTATAAAGGCGAGCGTAAAATCTCCTGGATGGAAATTTACACCGGTGAAAAATCCACACAGGTTTATGGTCAGGACGTCTGGCTGCCTGCTGAAACTCTTGATCTGATTCGTGAATATCGCGTTGCCATTAAAGGTCCGCTGACCACTCCTGTTGGTGGCGGTATTCGCTCTCTGAACGTTGCCCTGCGCCAGGAACTGGATCTCTACATCTGCCTGCGTCCGGTACGTTACTATCAAGGCACTCCAAGCCCGGTTAAACACCCTGAACTGACCGATATGGTTATCTTCCGTGAAAACTCGGAAGACATTTATGCGGGTATCGAATGGAAAGCAGACTCTGCCGACGCCGAGAAAGTGATTAAATTCCTGCGTGAAGAGATGGGGGTGAAGAAAATTCGCTTCCCGGAACATTGTGGTATCGGTATTAAGCCGTGTTCGGAAGAAGGCACCAAACGTCTGGTTCGTGCAGCGATCGAATACGCAATTGCTAACGATCGTGACTCTGTGACTCTGGTGCACAAAGGCAACATCATGAAGTTCACCGAAGGCGCGTTTAAAGACTGGGGCTACCAGCTGGCGCGTGAAGAGTTTGGCGGTGAACTGATCGACGGCGGCCCGTGGCTGAAAGTTAAAAACCCGAACACCGGCAAAGAGATCGTCATTAAAGACGTGATTGCTGATGCATTCCTGCAACAGATCTGGCTGGCGGTCCA
