# Supplementary material for: Antibiotic-resistant characteristics and horizontal gene transfer ability analysis of extended-spectrum β-lactamase-producing Escherichia coli isolated from giant pandas
Source: Front Vet Sci. 2024 Jul 26;11:1394814. doi: 10.3389/fvets.2024.1394814 (PMC11310934; doi:10.3389/fvets.2024.1394814)
Supplement: Supplementary file 5 [file Data_Sheet_5.docx]

>seq1[organism=Giant Panda Escherichia coli] Giant Panda Escherichia coli strain GP001, malate dehydrogenase gene.

TTTAAGAAATGTTCAGATCTCGGCGCTGCTGGCGGTATTGGCCAGGCGCTTGCACTACTGTTAAAAACCCAACTGCCTTCAGGTTCAGAACTCTCTCTGTATGATATCGCTCCAGTGACTCCCGGTGTGGCTGTCGATCTGAGCCATATCCCTACTGCTGTGAAAATCAAAGGTTTTTCAGGTGAAGATGCGACTCCGGCGCTGGAAGGTGCAGATGTCGTTCTTATCTCTGCAGGCGTAGCGCGTAAACCGGGTATGGATCGTTCCGACCTGTTTAACGTTAACGCCGGCATCGTGAAAAACCTGGTACAGCAAGTTGCGAAAACCTGCCCGAAAGCGTGCATTGGTATTATTACTAACCCGGTTAACACTACAGTTGCGATTGCTGCTGAAGTGCTGAAAAAAGCCGGTGTTTATGACAAAAACAAACTGTTCGGCGTTACCACGCTGGATATCATTCGTTCCAACACCTTTGTTGCGGAACTGAAAGGCAAACAGCCAGGCGAAGTTGAAGTGCCGGTTATTGGTGGTCACTCTGGTGTTACCATTCTGCCGCTGCTGTCACAGGTTCCTGGCGTTAGTTTTACCGAGCAGGAAGTGGCTGATCTGACCAAACGTATCCAGAACGCGGGTACTGAGGTGGTTGAAGCGAAAGCCGGTGGCGGGTCTGCAACCCTGTCTATGGGCCAGGCAGCTGCACGTTTTGGTCTGTCTCTGGTTCGCGCACTGCAGGGCGAACAAGGCGTTATCGAATGTGCCTACGTTGAAGGCGACGGTCAGTACGCTCGTTTCTTCTCTCAACCGCTGCTGCTGGGTAAAAACGGCGTGGAAGAGCGTAAATCTATCGGTACTCTGAGCGCATTTGAACAGAACGCGCTGGAAGGTATGCTGGATACGCTGAAGAAAGATATCGCTCTGGGGCAGATTTCGGTAAAAA

>seq2[organism=Giant Panda Escherichia coli] Giant Panda Escherichia coli strain GP003, malate dehydrogenase gene.

ATCTCGGCGCTGCTGGCGGTATTGGCCAGGCGCTTGCACTACTGTTAAAAACCCAACTGCCTTCAGGTTCAGAACTCTCTCTGTATGATATCGCTCCAGTGACTCCCGGTGTGGCTGTCGATCTGAGCCATATCCCTACTGCTGTGAAAATCAAAGGTTTTTCTGGTGAAGATGCGACTCCGGCGCTGGAAGGCGCAGATGTCGTTCTTATCTCTGCAGGCGTAGCGCGTAAACCGGGTATGGATCGTTCCGACCTGTTTAACGTTAACGCCGGCATCGTGAAAAACCTGGTACAGCAAGTTGCGAAAACCTGCCCGAAAGCGTGCATTGGTATTATCACTAACCCGGTTAACACCACAGTTGCAATTGCTGCTGAAGTGCTGAAAAAAGCCGGTGTTTATGACAAAAACAAACTGTTCGGCGTTACCACGCTGGATATCATTCGTTCCAACACCTTTGTTGCGGAACTGAAAGGCAAACAGCCAGGCGAAGTTGAAGTGCCGGTTATTGGCGGTCACTCTGGTGTTACCATTCTGCCGCTGCTGTCACAGGTTCCTGGCGTTAGTTTTACCGAGCAGGAAGTGGCTGATCTGACCAAACGCATCCAGAACGCGGGTACTGAAGTGGTTGAAGCGAAGGCCGGTGGCGGGTCTGCAACCCTGTCTATGGGCCAGGCAGCTGCACGTTTTGGTCTGTCTCTGGTTCGTGCACTGCAGGGCGAACAAGGCGTTGTCGAATGTGCCTACGTTGAAGGCGACGGTCAGTACGCCCGTTTCTTCTCTCAACCGCTGCTGCTGGGTAAAAACGGCGTGGAAGAGCGTAAATCTATCGGTACCCTGAGCGCATTTGAACAGAACGCGCTGGAAGGTATGCTGGATACGCTGAAGAAAGATATCGCTCTGGGGCAGGG

>seq3[organism=Giant Panda Escherichia coli] Giant Panda Escherichia coli strain GP004, malate dehydrogenase gene.

TAATAGAATGTGTCCAGATCTCGGCGCTGCTGGCGGTATTGGCCAGGCGCTTGCACTACTGTTAAAAACCCAACTGCCTTCAGGTTCAGAACTCTCTCTGTATGATATCGCTCCAGTGACTCCCGGTGTGGCTGTCGATCTGAGCCATATCCCTACTGCTGTGAAAATCAAAGGTTTTTCTGGTGAAGATGCGACTCCGGCGCTGGAAGGCGCAGATGTCGTTCTTATCTCTGCAGGCGTAGCGCGTAAACCGGGTATGGATCGTTCCGACCTGTTTAACGTTAACGCCGGCATCGTGAAAAACCTGGTACAGCAAGTTGCGAAAACCTGCCCGAAAGCGTGCATTGGTATTATCACTAACCCGGTTAACACCACAGTTGCAATTGCTGCTGAAGTGCTGAAAAAAGCCGGTGTTTATGACAAAAACAAACTGTTCGGCGTTACCACGCTGGATATCATTCGTTCCAACACCTTTGTTGCGGAACTGAAAGGCAAACAGCCAGGCGAAGTTGAAGTGCCGGTTATTGGCGGTCACTCTGGTGTTACCATTCTGCCGCTGCTGTCACAGGTTCCTGGCGTTAGTTTTACCGAGCAGGAAGTGGCTGATCTGACCAAACGCATCCAGAACGCGGGTACTGAAGTGGTTGAAGCGAAGGCCGGTGGCGGGTCTGCAACCCTGTCTATGGGCCAGGCAGCTGCACGTTTTGGTCTGTCTCTGGTTCGTGCACTGCAGGGCGAACAAGGCGTTGTCGAATGTGCCTACGTTGAAGGCGACGGTCAGTACGCCCGTTTCTTCTCTCAACCGCTGCTGCTGGGTAAAAACGGCGTGGAAGAGCGTAAATCTATCGGTACCCTGAGCGCATTTGAACAGAACGCGCTGGAAGGTATGCTGGATACGCTGAAGAAAGATATCGCTCTGGGGCAGGG

>seq4[organism=Giant Panda Escherichia coli] Giant Panda Escherichia coli strain GP012, malate dehydrogenase gene.

ATATGAAAGGGTCGGATCCTCGGCGCTGCTGGCGGTATTGGCCAGGCGCTTGCACTACTGTTAAAAACCCAACTGCCTTCAGGTTCAGAACTCTCTCTGTATGATATCGCTCCAGTGACTCCCGGTGTGGCTGTCGATCTGAGCCATATCCCTACTGCTGTGAAAATCAAAGGTTTTTCAGGTGAAGATGCGACTCCGGCGCTGGAAGGTGCAGATGTCGTTCTTATCTCTGCAGGCGTAGCGCGTAAACCGGGTATGGATCGTTCCGACCTGTTTAACGTTAACGCCGGTATCGTGAAAAACCTGGTACAGCAAGTTGCGAAAACCTGCCCGAAAGCGTGCATTGGTATTATCACTAACCCGGTTAACACTACAGTTGCGATTGCTGCTGAAGTGCTGAAAAAAGCCGGTGTTTATGACAAAAACAAACTGTTCGGCGTTACCACGCTGGATATCATTCGTTCCAACACCTTTGTTGCGGAACTGAAAGGCAAACAGCCAGGCGAAGTTGAAGTGCCGGTTATTGGTGGTCACTCTGGTGTTACCATTCTGCCGCTGCTGTCACAGGTTCCTGGCGTTAGTTTTACCGAGCAGGAAGTGGCTGATCTGACCAAACGTATCCAGAACGCGGGTACTGAGGTGGTTGAAGCGAAAGCCGGTGGCGGGTCTGCAACCCTGTCTATGGGCCAGGCAGCTGCACGTTTTGGTCTGTCTCTGGTTCGCGCACTGCAGGGCGAACAAGGCGTTGTCGAATGTGCCTACGTTGAAGGCGACGGTCAGTACGCTCGTTTCTTCTCTCAACCGCTGCTGCTGGGTAAAAACGGCGTGGAAGAGCGTAAATCTATCGGTACTCTGAGCGCATTTGAACAGAACGCGCTGGAAGTATGCTGGATACGCTGAAGAAAGATATCGCTCTGGGCAGAAGTTCTCCGTTAAAA

>seq5[organism=Giant Panda Escherichia coli] Giant Panda Escherichia coli strain GP014, malate dehydrogenase gene.

TATTGAAAAGTTTCGATCCTCGGCGCTGCTGGCGGTATTGGCCAGGCGCTTGCACTACTGTTAAAAACCCAACTGCCTTCAGGTTCAGAACTCTCTCTGTATGATATCGCTCCAGTGACTCCCGGTGTGGCTGTCGATCTGAGCCATATCCCTACTGCTGTGAAAATCAAAGGTTTTTCTGGTGAAGATGCGACTCCGGCGCTGGAAGGCGCAGATGTCGTTCTTATCTCTGCAGGCGTAGCGCGTAAACCGGGTATGGATCGTTCCGACCTGTTTAACGTTAACGCCGGCATCGTGAAAAACCTGGTACAGCAAGTTGCGAAAACCTGCCCGAAAGCGTGCATTGGTATTATCACTAACCCGGTTAACACCACAGTTGCAATTGCTGCTGAAGTGCTGAAAAAAGCCGGTGTTTATGACAAAAACAAACTGTTCGGCGTTACCACGCTGGATATCATTCGTTCCAACACCTTTGTTGCGGAACTGAAAGGCAAACAGCCAGGCGAAGTTGAAGTGCCGGTTATTGGCGGTCACTCTGGTGTTACCATTCTGCCGCTGCTGTCACAGGTTCCTGGCGTTAGTTTTACCGAGCAGGAAGTGGCTGATCTGACCAAACGCATCCAGAACGCGGGTACTGAAGTGGTTGAAGCGAAGGCCGGTGGCGGGTCTGCAACCCTGTCTATGGGCCAGGCAGCTGCACGTTTTGGTCTGTCTCTGGTTCGTGCACTGCAGGGCGAACAAGGCGTTGTCGAATGTGCCTACGTTGAAGGCGACGGTCAGTACGCCCGTTTCTTCTCTCAACCGCTGCTGCTGGGTAAAAACGGCGTGGAAGAGCGTAAATCTATCGGTACCCTGAGCGCATTTGAACAGAACGCGCTGGAAGGTATGCTGGATACGCTGAAGAAAGATATCGCTCTGGGGCAGGAGTTTCGATTTAATATATAAAAAAA

>seq6[organism=Giant Panda Escherichia coli] Giant Panda Escherichia coli strain GP022, malate dehydrogenase gene.

TAAATGAGTTGTGTCGCATCCTCGGCGCTGCTGGCGGTATTGGCCAGGCGCTTGCACTACTGTTAAAAACCCAACTGCCTTCAGGTTCAGAACTCTCTCTGTATGATATCGCTCCAGTGACTCCCGGTGTGGCTGTCGATCTGAGCCATATCCCTACTGCTGTGAAAATCAAAGGTTTTTCTGGTGAAGATGCGACTCCGGCGCTGGAAGGCGCAGATGTCGTTCTTATCTCTGCAGGTGTAGCGCGTAAACCGGGTATGGATCGTTCCGACCTGTTTAACGTTAACGCCGGCATCGTGAAAAACCTGGTACAGCAAGTTGCGAAAACCTGCCCGAAAGCGTGCATTGGTATTATCACTAACCCGGTTAACACCACAGTGGCGATTGCTGCTGAAGTGCTGAAAAAAGCCGGTGTTTATGACAAAAACAAACTGTTCGGCGTTACCACGCTGGATATCATTCGTTCCAACACCTTTGTTGCGGAACTGAAAGGCAAACAGCCAGGCGAAGTTGAAGTGCCGGTTATTGGCGGTCACTCTGGTGTTACCATTCTGCCGCTGCTGTCACAGGTTCCTGGCGTTAGTTTTACCGAGCAGGAAGTGGCTGATCTGACCAAACGTATCCAGAACGCGGGTACTGAGGTGGTTGAAGCGAAAGCCGGTGGCGGGTCTGCAACCCTGTCTATGGGTCAGGCAGCTGCACGTTTTGGCCTGTCTCTGGTTCGCGCACTGCAAGGTGAACAAGGCGTTGTCGAATGTGCCTACGTTGAAGGTGACGGTCAGTACGCACGTTTCTTCTCTCAACCGCTGCTGCTGGGTAAAAACGGCGTGGAAGAGCGTAAATCTATCGGCACTCTGAGCGCATTTGAAAAGAACGCGCTGGAAGGTATGCTGGATACGCTGAAGAAAGATATCGCTCTGGGGCAGATTTCTCTTTAAAATTA

>seq7[organism=Giant Panda Escherichia coli] Giant Panda Escherichia coli strain GP030, malate dehydrogenase gene.

TATAGTAAATTGTGGGATCCTCGGCGCTGCTGGCGGTATTGGCCAGGCGCTTGCACTACTGTTAAAAACCCAACTGCCTTCAGGTTCAGAACTCTCTCTGTATGATATCGCTCCAGTGACTCCCGGTGTGGCTGTCGATCTGAGCCATATCCCTACTGCTGTGAAAATCAAAGGTTTTTCTGGTGAAGATGCGACTCCGGCGCTGGAAGGCGCAGATGTCGTTCTTATCTCTGCAGGCGTAGCGCGTAAACCGGGTATGGATCGTTCCGACCTGTTTAACGTTAACGCCGGCATCGTGAAAAACCTGGTACAGCAAGTTGCGAAAACCTGCCCGAAAGCGTGCATTGGTATTATCACTAACCCGGTTAACACCACAGTTGCAATTGCTGCTGAAGTGCTGAAAAAAGCCGGTGTTTATGACAAAAACAAACTGTTCGGCGTTACCACGCTGGATATCATTCGTTCCAACACCTTTGTTGCGGAACTGAAAGGCAAACAGCCAGGCGAAGTTGAAGTGCCGGTTATTGGCGGTCACTCTGGTGTTACCATTCTGCCGCTGCTGTCACAGGTTCCTGGCGTTAGTTTTACCGAGCAGGAAGTGGCTGATCTGACCAAACGCATCCAGAACGCGGGTACTGAAGTGGTTGAAGCGAAGGCCGGTGGCGGGTCTGCAACCCTGTCTATGGGCCAGGCAGCTGCACGTTTTGGTCTGTCTCTGGTTCGTGCACTGCAGGGCGAACAAGGCGTTGTCGAATGTGCCTACGTTGAAGGCGACGGTCAGTACGCCCGTTTCTTCTCTCAACCGCTGCTGCTGGGTAAAAACGGCGTGGAAGAGCGTAAATCTATCGGTACCCTGAGCGCATTTGAACAGAACGCGCTGGAAGGTATGCTGGATACGCTGAAGAAAGATATCGCTCTGGGGCAGAATTTCGTTAAAA

>seq8[organism=Giant Panda Escherichia coli] Giant Panda Escherichia coli strain GP032, malate dehydrogenase gene.

GTCCCATCTCGGCGCTGCTGGCGGTATTGGCCAGGCGCTTGCACTACTGTTAAAAACCCAACTGCCTTCAGGTTCAGAACTCTCTCTGTATGATATCGCTCCAGTGACTCCCGGTGTGGCTGTCGATCTGAGCCATATCCCTACTGCTGTGAAAATCAAAGGTTTTTCTGGTGAAGATGCGACTCCGGCGCTGGAAGGCGCAGATGTCGTTCTTATCTCTGCAGGCGTAGCGCGTAAACCGGGTATGGATCGTTCCGACCTGTTTAACGTTAACGCTGGCATCGTAAAAAACCTGGTACAGCAAGTTGCGAAAACTTGCCCGAAAGCGTGCATTGGTATTATCACTAACCCGGTTAACACTACAGTGGCGATTGCTGCTGAAGTGCTGAAAAAAGCCGGTGTTTATGACAAAAACAAACTGTTCGGCGTTACCACGCTGGATATCATTCGTTCCAACACCTTTGTTGCGGAACTGAAAGGCAAACAGCCAGGCGAAGTTGAAGTGCCGGTTATTGGCGGTCACTCTGGTGTTACCATTCTGCCGCTGCTGTCACAGGTTCCTGGCGTTAGTTTTACCGAGCAGGAAGTGGCTGATCTGACCAAACGTATCCAGAACGCGGGTACTGAGGTGGTTGAAGCGAAAGCCGGTGGCGGGTCTGCAACCCTGTCTATGGGTCAGGCAGCTGCACGTTTTGGTCTGTCTCTGGTACGCGCACTGCAGGGCGAACAAGGCGTTGTCGAATGTGCCTACGTTGAAGGTGACGGTCAGTACGCACGTTTCTTCTCTCAACCGCTGCTGCTGGGTAAAAACGGCGTGGAAGAGCGTAAATCTATCGGCACTCTGAGCGCATTTGAAAAGAACGCGCTGGAAGGTATGCTGGATACGCTGAAGAAAGATATCGCTCTGGGGAAGGAT

>seq9[organism=Giant Panda Escherichia coli] Giant Panda Escherichia coli strain GP050, malate dehydrogenase gene.

TGGCTATGAAAAATGTTCGATCCTCGGCGCTGCTGGCGGTATTGGCCAGGCGCTTGCACTACTGTTAAAAACCCAACTGCCTTCAGGTTCAGAACTCTCTCTGTATGATATCGCTCCAGTGACTCCCGGTGTGGCTGTCGATCTGAGCCATATCCCTACTGCTGTGAAAATCAAAGGTTTTTCAGGTGAAGATGCGACTCCGGCGCTGGAAGGTGCAGATGTCGTTCTTATCTCTGCAGGCGTAGCGCGTAAACCGGGTATGGATCGTTCCGACCTGTTTAACGTTAACGCCGGCATCGTGAAAAACTTGGTACAGCAAGTTGCGAAAACCTGCCCGAAAGCGTGCATTGGTATTATCACTAACCCGGTTAACACTACAGTTGCGATTGCTGCTGAAGTGCTGAAAAAAGCCGGTGTTTATGACAAAAACAAACTGTTCGGCGTTACCACGCTGGATATCATTCGTTCCAACACCTTTGTTGCGGAACTGAAAGGCAAACAGCCAGGCGAAGTTGAAGTGCCGGTTATTGGTGGTCACTCTGGTGTTACCATTCTGCCGCTGCTGTCACAGGTTCCTGGCGTTAGTTTTACCGAGCAGGAAGTGGCTGATCTGACCAAACGTATCCAGAACGCGGGTACTGAGGTGGTTGAAGCGAAAGCCGGTGGCGGGTCTGCAACCCTGTCTATGGGCCAGGCAGCTGCACGTTTTGGTCTGTCTCTGGTTCGCGCACTGCAGGGCGAACAAGGCGTTGTCGAATGTGCCTACGTTGAAGGCGACGGTCAGTACGCTCGTTTCTTCTCTCAACCGCTGCTGCTGGGTAAAAACGGCGTGGAAGAGCGTAAATCTATCGGTACTCTGAGCGCATTTGAACAGAACGCGCTGGAAGGTATGCTGGATACGCTGAAGAAAGATATCGCTCTGGGGCAGGATTTTCGTAAAAAAAAA

>seq10[organism=Giant Panda Escherichia coli] Giant Panda Escherichia coli strain GP065, malate dehydrogenase gene.

TTAGAGAAGATTGCGGATCCTCGGCGCTGCTGGCGGTATTGGCCAGGCGCTTGCACTACTGTTAAAAACCCAACTGCCTTCAGGTTCAGAACTCTCTCTGTATGATATCGCTCCAGTGACTCCCGGTGTGGCTGTCGATCTGAGCCATATCCCTACTGCTGTGAAAATCAAAGGTTTTTCTGGTGAAGATGCGACTCCGGCGCTGGAAGGCGCAGATGTCGTTCTTATCTCTGCAGGCGTAGCGCGTAAACCGGGTATGGATCGTTCCGACCTGTTTAACGTTAACGCCGGCATCGTGAAAAACCTGGTACAGCAAGTTGCGAAAACCTGCCCGAAAGCGTGCATTGGTATTATCACTAACCCGGTTAACACCACAGTTGCAATTGCTGCTGAAGTGCTGAAAAAAGCCGGTGTTTATGACAAAAACAAACTGTTCGGCGTTACCACGCTGGATATCATTCGTTCCAACACCTTTGTTGCGGAACTGAAAGGCAAACAGCCAGGCGAAGTTGAAGTGCCGGTTATTGGCGGTCACTCTGGTGTTACCATTCTGCCGCTGCTGTCACAGGTTCCTGGCGTTAGTTTTACCGAGCAGGAAGTGGCTGATCTGACCAAACGCATCCAGAACGCGGGTACTGAAGTGGTTGAAGCGAAGGCCGGTGGCGGGTCTGCAACCCTGTCTATGGGCCAGGCAGCTGCACGTTTTGGTCTGTCTCTGGTTCGTGCACTGCAGGGCGAACAAGGCGTTGTCGAATGTGCCTACGTTGAAGGCGACGGTCAGTACGCCCGTTTCTTCTCTCAACCGCTGCTGCTGGGTAAAAACGGCGTGGAAGAGCGTAAATCTATCGGTACCCTGAGCGCATTTGAACAGAACGCGCTGGAAGGTATGCTGGATACGCTGAAGAAAGATATCGCTCTGGGGCAGAG

>seq11[organism=Giant Panda Escherichia coli] Giant Panda Escherichia coli strain GP095, malate dehydrogenase gene.

AGATATAGAAAATGTTGGAGTCCTCGGCGCTGCTGGCGGTATTGGCCAGGCGCTTTGCACTACTGTTAAAAACCCAACTGCCTTCAGGTTCAGAACTCTCTCTGTATGATATCGCTCCAGTGACTCCCGGTGTGGCTGTCGATCTGAGCCATATCCCTACTGCTGTGAAAATCAAAGGTTTTTCTGGTGAAGATGCGACTCCGGCGCTGGAAGGCGCAGATGTCGTTCTTATCTCTGCAGGTGTAGCGCGTAAACCGGGTATGGATCGTTCCGACCTGTTTAACGTTAACGCCGGCATCGTGAAAAACCTGGTACAGCAAGTTGCGAAAACCTGCCCGAAAGCGTGCATTGGTATTATCACTAACCCGGTTAACACCACAGTGGCGATTGCTGCTGAAGTGCTGAAAAAAGCCGGTGTTTATGACAAAAACAAACTGTTCGGCGTTACCACGCTGGATATCATTCGTTCCAACACCTTTGTTGCGGAACTGAAAGGCAAACAGCCAGGCGAAGTTGAAGTGCCGGTTATTGGCGGTCACTCTGGTGTTACCATTCTGCCGCTGCTGTCACAGGTTCCTGGCGTTAGTTTTACCGAGCAGGAAGTGGCTGATCTGACCAAACGTATCCAGAACGCGGGTACTGAGGTGGTTGAAGCGAAAGCCGGTGGCGGGTCTGCAACCCTGTCTATGGGTCAGGCAGCTGCACGTTTTGGCCTGTCTCTGGTTCGCGCACTGCAAGGTGAACAAGGCGTTGTCGAATGTGCCTACGTTGAAGGCGACGGTCAGTACGCACGTTTCTTCTCTCAACCGCTGCTGCTGGGTAAAAACGGCGTGGAAGAGCGTAAATCTATCGGCACTCTGAGCGCATTTGAACAGAACGCGCTGGAAGGGTATGCTGGATACGCTGAAGAAAGATATCGCTCTGGGGCAGAGTTTCCGTTAAAATTACAGA

>seq12[organism=Giant Panda Escherichia coli] Giant Panda Escherichia coli strain GP101, malate dehydrogenase gene.

TAAAGAAAAATTTCCATCCTCGGCGCTGCTGGCGGTATTGGCCAGGCGCTTGCACTACTGTTAAAAACCCAACTGCCTTCAGGTTCAGAACTCTCTCTGTATGATATCGCTCCAGTGACTCCCGGTGTGGCTGTCGATCTGAGCCATATCCCTACTGCTGTGAAAATCAAAGGTTTTTCTGGTGAAGATGCGACTCCGGCGCTGGAAGGCGCAGATGTCGTTCTTATCTCTGCAGGTGTAGCGCGTAAACCGGGTATGGATCGTTCCGACCTGTTTAACGTTAACGCCGGCATCGTGAAAAACCTGGTACAGCAAGTTTCGAAAACCTGCCCGAAAGCGTGCATTGGTATTATCACTAACCCGGTTAACACCACAGTTGCGATTGCTGCTGAAGTGCTGAAAAAAGCCGGTGTTTATGACAAAAACAAACTGTTCGGCGTTACCACGCTGGATATCATTCGTTCCAACACCTTTGTTGCGGAACTGAAAGGCAAACAGCCAGGCGAAGTTGAAGTGCCGGTTATTGGCGGTCACTCTGGTGTTACCATTCTGCCGCTGCTGTCACAGGTTCCTGGCGTTAGTTTTACCGAGCAGGAAGTGGCTGATCTGACCAAACGTATCCAGAACGCAGGTACTGAAGTGGTTGAAGCGAAAGCCGGTGGCGGGTCTGCAACCCTGTCTATGGGCCAGGCAGCTGCACGTTTTGGTCTGTCTCTGGTACGCGCACTGCAGGGCGAACAAGGCGTTGTCGAATGTGCCTATGTTGAAGGCGACGGTCAGTACGCACGTTTCTTCTCTCAACCGCTGCTGCTGGGTAAAAACGGCGTGGAAGAGCGTAAATCTATCGGTACCCTGAGCGCATTTGAACAGAGCGCACTGGAAGGTATGCTGGATACGCTGAAGAAAGATATCGCTCTGGGGCAGATC
