# Supplementary material for: Antibiotic-resistant characteristics and horizontal gene transfer ability analysis of extended-spectrum β-lactamase-producing Escherichia coli isolated from giant pandas
Source: Front Vet Sci. 2024 Jul 26;11:1394814. doi: 10.3389/fvets.2024.1394814 (PMC11310934; doi:10.3389/fvets.2024.1394814)
Supplement: Supplementary file 6 [file Data_Sheet_6.docx]

>seq1[organism=Giant Panda Escherichia coli] Giant Panda Escherichia coli strain GP001, adenylosuccinate synthase gene.

GGGTTTGCTGTCTCCGGCTGCGCTGATGAAAGAGATGAAAGAACTGGAAGACCGTGGCATCCCCGTTCGTGAGCGTCTGCTGCTGTCCGAAGCATGTCCGCTGATCCTTGATTATCACGTTGCGCTGGATAACGCGCGTGAGAAAGCGCGTGGCGCGAAAGCGATCGGCACCACCGGTCGTGGTATCGGGCCTGCTTATGAAGATAAAGTGGCACGTCGCGGTCTGCGTGTTGGCGACCTTTTCGACAAAGAAACCTTCGCTGAAAAACTGAAAGAAGTGATGGAATATCACAACTTCCAGTTGGTTAACTACTACAAAGCTGAAGCGGTTGATTACCAGAAAGTTCTGGATGATACGATGGCTGTTGCCGACATCCTGACTTCTATGGTTGTTGACGTTTCTGATCTGCTCGACCAGGCGCGTCAGCGTGGCGATTTCGTCATGTTTGAAGGTGCGCAGGGTACGCTGCTGGATATCGACCACGGCACTTATCCGTACGTAACTTCTTCCAACACCACTGCTGGTGGCGTGGCGACCGGTTCCGGCCTGGGCCCGCGTTATGTTGATTACGTTCTGGGTATCCTCAAAGCTTACTCCACTCGTGTGGGTGCAGGTCCGTTCCCGACCGAACTGTTTGATGAAACTGGCGAGTTCCTCTGCAAGCAGGGTAACGAATTCGGCGCAACTACGGGTCGTCGTCGTCGTACCGGCTGGCTGGACACCGTTGCCGTTCGTCGTGCGGTACAGCTGAACTCTCTGTCTGGCTTCTGCCTGACTAAACTGGACGTTCTGGATGGCCTGAAAGAGGTTAAACTCTGCTTCT

>seq2[organism=Giant Panda Escherichia coli] Giant Panda Escherichia coli strain GP003, adenylosuccinate synthase gene.

GGGGGGCTGTCTCCGGCCGCGCTGATGAAAGAGATGAAAGAACTGGAAGACCGTGGCATCCCCGTTCGTGAGCGTCTGCTGCTGTCTGAAGCATGTCCGCTGATCCTTGATTATCACGTTGCGCTGGATAACGCGCGTGAGAAAGCGCGTGGCGCGAAAGCGATCGGCACCACCGGTCGTGGTATCGGGCCTGCTTATGAAGATAAAGTAGCACGTCGCGGTCTGCGTGTTGGCGACCTTTTCGACAAAGAAACCTTCGCTGAAAAACTGAAAGAAGTGATGGAATATCACAACTTCCAGTTGGTTAACTACTACAAAGCTGAAGCGGTTGATTACCAGAAAGTTCTGGATGATACGATGGCTGTTGCCGACATCCTGACTTCTATGGTGGTTGACGTTTCTGACCTGCTCGACCAGGCGCGTCAGCGTGGCGATTTCGTCATGTTTGAAGGTGCGCAGGGTACGCTGCTGGATATCGACCACGGTACTTATCCGTACGTAACTTCTTCCAACACCACTGCTGGTGGCGTGGCGACCGGTTCCGGCCTGGGCCCGCGTTATGTTGATTACGTTCTGGGTATCCTCAAAGCTTACTCCACTCGTGTAGGTGCAGGTCCGTTCCCGACCGAACTGTTTGATGAAACTGGCGAGTTCCTCTGCAAGCAGGGTAACGAATTCGGCGCAACTACGGGGCGTCGTCGTCGTACCGGCTGGCTGGACACCGTTGCCGTTCGTCGTGCGGTACAGCTGAACTCCCTGTCTGGCTTCTGCCTGACTAAACTGGACGTTCTGGATGGCCTGAAAGAGGTTAAACTCTGCT

>seq3[organism=Giant Panda Escherichia coli] Giant Panda Escherichia coli strain GP004, adenylosuccinate synthase gene.

GGGTTTGCTGTCTCCGGCCGCGCTGATGAAAGAGATGAAAGAACTGGAAGACCGTGGCATCCCCGTTCGTGAGCGTCTGCTGCTGTCTGAAGCATGTCCGCTGATCCTTGATTATCACGTTGCGCTGGATAACGCGCGTGAGAAAGCGCGTGGCGCGAAAGCGATCGGCACCACCGGTCGTGGTATCGGGCCTGCTTATGAAGATAAAGTAGCACGTCGCGGTCTGCGTGTTGGCGACCTTTTCGACAAAGAAACCTTCGCTGAAAAACTGAAAGAAGTGATGGAATATCACAACTTCCAGTTGGTTAACTACTACAAAGCTGAAGCGGTTGATTACCAGAAAGTTCTGGATGATACGATGGCTGTTGCCGACATCCTGACTTCTATGGTGGTTGACGTTTCTGACCTGCTCGACCAGGCGCGTCAGCGTGGCGATTTCGTCATGTTTGAAGGTGCGCAGGGTACGCTGCTGGATATCGACCACGGTACTTATCCGTACGTAACTTCTTCCAACACCACTGCTGGTGGCGTGGCGACCGGTTCCGGCCTGGGCCCGCGTTATGTTGATTACGTTCTGGGTATCCTCAAAGCTTACTCAACTCGTGTAGGTGCAGGTCCTTTCCCGACCGAACTGTTTGATGAAACTGGCGAGTTCCTCTGCAAGCAGGGTAACGAATTCGGCGCAACTACGGGGCGTCGTCGTCGTACCGGCTGGCTGGACACCGTTGCCGTTCGTCGTGCGGTACAGCTGAACTCCCTGTCTGGCTTCTGCCTGACTAAACTGGACGTTCTGGATGGCCTGAAAGAGGTTAAACTCTGT

>seq4[organism=Giant Panda Escherichia coli] Giant Panda Escherichia coli strain GP012, adenylosuccinate synthase gene.

GCTGGCTGTCTCCGGCTGCGCTGATGAAAGAGATGAAAGAACTGGAAGACCGTGGCATCCCCGTTCGTGAGCGTCTGCTGCTGTCCGAAGCATGTCCGCTGATCCTTGATTATCACGTTGCGCTGGATAACGCGCGTGAGAAAGCGCGTGGCGCGAAAGCGATCGGCACCACCGGTCGTGGTATCGGGCCTGCTTATGAAGATAAAGTAGCACGTCGCGGTCTGCGTGTTGGCGACCTTTTCGACAAAGAAACCTTCGCTGAAAAACTGAAAGAAGTGATGGAATATCACAACTTCCAGTTGGTTAACTACTACAAAGCTGAAGCGGTTGATTACCAGAAAGTTCTGGATGATACGATGGCTGTTGCCGACATCCTGACTTCTATGGTTGTTGACGTTTCTGACCTGCTCGACCAGGCGCGTCAGCGTGGCGATTTCGTCATGTTTGAAGGTGCGCAGGGTACGCTGCTGGATATCGACCACGGTACATATCCGTACGTAACTTCTTCCAACACCACTGCTGGTGGCGTGGCGACCGGTTCCGGCCTGGGCCCGCGTTATGTTGATTACGTTCTGGGTATCCTCAAAGCTTACTCCACTCGTGTGGGGGCAGGTCCGTTCCCGACCGAACTGTTTGATGAAACTGGCGAGTTCCTCTGCAAGCAGGGTAACGAATTCGGCGCAACTACGGGTCGTCGTCGTCGTACCGGCTGGCTGGACACCGTTGCCGTTCGTCGTGCGGTACAGTTGAACTCCCTGTCTGGCTTCTGCCTGACTAAACTGGACGTTCTGGATGGCCTGAAAGAGGTTAAACTCTGTTTT

>seq5[organism=Giant Panda Escherichia coli] Giant Panda Escherichia coli strain GP014, adenylosuccinate synthase gene.

GGGTTTGCTGTCTCCGGCCGCGCTGATGAAAGAGATGAAAGAACTGGAAGACCGTGGCATCCCCGTTCGTGAGCGTCTGCTGCTGTCTGAAGCATGTCCGCTGATCCTTGATTATCACGTTGCGCTGGATAACGCGCGTGAGAAAGCGCGTGGCGCGAAAGCGATCGGCACCACCGGTCGTGGTATCGGGCCTGCTTATGAAGATAAAGTAGCACGTCGCGGTCTGCGTGTTGGCGACCTTTTCGACAAAGAAACCTTCGCTGAAAAACTGAAAGAAGTGATGGAATATCACAACTTCCAGTTGGTTAACTACTACAAAGCTGAAGCGGTTGATTACCAGAAAGTTCTGGATGATACGATGGCTGTTGCCGACATCCTGACTTCTATGGTGGTTGACGTTTCTGACCTGCTCGACCAGGCGCGTCAGCGTGGCGATTTCGTCATGTTTGAAGGTGCGCAGGGTACGCTGCTGGATATCGACCACGGTACTTATCCGTACGTAACTTCTTCCAACACCACTGCTGGTGGCGTGGCGACCGGTTCCGGCCTGGGCCCGCGTTATGTTGATTACGTTCTGGGTATCCTCAAAGCTTACTCAACTCGTGTAGGTGCAGGTCCTTTCCCGACCGAACTGTTTGATGAAACTGGCGAGTTCCTCTGCAAGCAGGGTAACGAATTCGGCGCAACTACGGGGCGTCGTCGTCGTACCGGCTGGCTGGACACCGTTGCCGTTCGTCGTGCGGTACAGCTGAACTCCCTGTCTGGCTTCTGCCTGACTAAACTGGACGTTCTGGATGGCCTGAAAGAGGTTAAACTCTGCTTTTT

>seq6[organism=Giant Panda Escherichia coli] Giant Panda Escherichia coli strain GP022, adenylosuccinate synthase gene.

GGGTTGTGCTGTCTCCGGCTGCGCTGATGAAAGAGATGAAAGAACTGGAAGACCGTGGCATCCCCGTTCGTGAGCGTCTGCTGCTGTCTGAAGCATGTCCGCTGATCCTTGATTATCACGTTGCGCTGGATAACGCGCGTGAGAAAGCGCGTGGCGCGAAAGCGATCGGCACCACCGGTCGTGGTATCGGGCCTGCTTATGAAGATAAAGTGGCACGTCGCGGTCTGCGTGTTGGCGACCTTTTCGACAAAGAAACCTTCGCTGAAAAACTGAAAGAAGTGATGGAATATCACAACTTCCAGTTGGTTAACTACTACAAAGCTGAAGCGGTTGATTACCAGAAAGTTCTGGATGATACGATGGCTGTTGCCGACATCCTGACTTCTATGGTTGTTGACGTTTCTGATCTGCTCGACCAGGCGCGTCAGCGTGGCGATTTCGTCATGTTTGAAGGTGCGCAGGGTACGCTGCTGGATATCGACCACGGTACTTATCCGTACGTAACTTCTTCCAACACCACTGCTGGTGGCGTGGCGACCGGTTCCGGCCTGGGCCCACGTTATGTTGATTACGTTCTGGGTATCCTCAAAGCTTACTCCACTCGTGTGGGTGCAGGTCCGTTCCCGACTGAACTGTTTGATGAAACTGGCGAGTTCCTCTGCAAGCAGGGTAACGAATTCGGCGCAACTACGGGTCGTCGTCGTCGTACCGGCTGGCTGGACACCGTTGCCGTTCGTCGTGCGGTACAGCTGAACTCCCTGTCTGGCTTCTGCCTGACCAAGCTGGACGTTCTGGATGGCCTGAAAGAGGTGAAACTCTGCTTTT

>seq7[organism=Giant Panda Escherichia coli] Giant Panda Escherichia coli strain GP030, adenylosuccinate synthase gene.

GGGGGCTGTCTCCGGCCGCGCTGATGAAAGAGATGAAAGAACTGGAAGACCGTGGCATCCCCGTTCGTGAGCGTCTGCTGCTGTCTGAAGCATGTCCGCTGATCCTTGATTATCACGTTGCGCTGGATAACGCGCGTGAGAAAGCGCGTGGCGCGAAAGCGATCGGCACCACCGGTCGTGGTATCGGGCCTGCTTATGAAGATAAAGTAGCACGTCGCGGTCTGCGTGTTGGCGACCTTTTCGACAAAGAAACCTTCGCTGAAAAACTGAAAGAAGTGATGGAATATCACAACTTCCAGTTGGTTAACTACTACAAAGCTGAAGCGGTTGATTACCAGAAAGTTCTGGATGATACGATGGCTGTTGCCGACATCCTGACTTCTATGGTGGTTGACGTTTCTGACCTGCTCGACCAGGCGCGTCAGCGTGGCGATTTCGTCATGTTTGAAGGTGCGCAGGGTACGCTGCTGGATATCGACCACGGTACTTATCCGTACGTAACTTCTTCCAACACCACTGCTGGTGGCGTGGCGACCGGTTCCGGCCTGGGCCCGCGTTATGTTGATTACGTTCTGGGTATCCTCAAAGCTTACTCCACTCGTGTAGGTGCAGGTCCGTTCCCGACCGAACTGTTTGATGAAACTGGCGAGTTCCTCTGCAAGCAGGGTAACGAATTCGGCGCAACTACGGGGCGTCGTCGTCGTACCGGCTGGCTGGACACCGTTGCCGTTCGTCGTGCGGTACAGCTGAACTCCCTGTCTGGCTTCTGCCTGACTAAACTGGACGTTCTGGATGGCCTGAAAGAGGTTAAACTCTGTTTTT

>seq8[organism=Giant Panda Escherichia coli] Giant Panda Escherichia coli strain GP032, adenylosuccinate synthase gene.

GGCTGTGCTGTCTCCGGCTGCGCTGATGAAAGAGATGAAAGAACTGGAAGACCGTGGCATCCCCGTTCGTGAGCGTCTGCTGCTGTCTGAAGCATGTCCGCTGATCCTTGATTATCACGTTGCGCTGGATAACGCGCGTGAGAAAGCGCGTGGCGCGAAAGCGATCGGCACCACCGGTCGTGGTATCGGGCCTGCTTATGAAGATAAAGTGGCACGTCGCGGTCTGCGTGTTGGCGACCTTTTCGACAAAGAAACCTTCGCTGAAAAACTGAAAGAAGTGATGGAATATCACAACTTCCAGTTGGTTAACTACTACAAAGCTGAAGCGGTTGATTACCAGAAAGTTCTGGATGATACGATGGCTGTTGCCGACATCCTGACTTCTATGGTTGTTGACGTTTCTGATCTGCTCGACCAGGCGCGTCAGCGTGGCGATTTCGTCATGTTTGAAGGTGCGCAGGGTACGCTGCTGGATATCGACCACGGTACTTATCCGTACGTAACTTCTTCCAACACCACTGCTGGTGGCGTGGCGACCGGTTCCGGCCTGGGCCCACGTTATGTTGATTACGTTCTGGGTATCCTCAAAGCTTACTCCACTCGTGTGGGTGCAGGTCCGTTCCCGACTGAACTGTTTGATGAAACTGGCGAGTTCCTCTGCAAGCAGGGTAACGAATTCGGCGCAACTACGGGTCGTCGTCGTCGTACCGGCTGGCTGGACACCGTTGCCGTTCGTCGTGCGGTACAGCTGAACTCCCTGTCTGGCTTCTGCCTGACCAAGCTGGACGTTCTGGATGGCCTGAAAGAGGTGAAACTCTGCTTC

>seq9[organism=Giant Panda Escherichia coli] Giant Panda Escherichia coli strain GP050, adenylosuccinate synthase gene.

GGGGGCTGTCTCCGGCTGCGCTGATGAAAGAGATGAAAGAACTGGAAGACCGTGGCATCCCCGTTCGTGAGCGTCTGCTGCTGTCCGAAGCATGTCCGCTGATCCTTGATTATCACGTTGCGCTGGATAACGCGCGTGAGAAAGCGCGTGGCGCGAAAGCGATCGGCACCACCGGTCGAGGTATCGGGCCTGCTTATGAAGATAAAGTGGCACGTCGCGGTCTGCGTGTTGGCGACCTTTTCGACAAAGAAACCTTCGCTGAAAAACTGAAAGAAGTGATGGAATATCACAACTTCCAGTTGGTTAACTACTACAAAGCTGAAGCGGTTGATTACCAGAAAGTTCTGGATGATACGATGGCTGTTGCCGACATCCTGACTTCTATGGTTGTTGACGTTTCTGACCTGCTTGACCAGGCGCGTCAGCGTGGCGATTTCGTCATGTTTGAAGGTGCGCAGGGTACGCTGCTGGATATCGACCACGGTACATATCCGTACGTAACTTCTTCCAACACCACTGCTGGTGGCGTGGCGACCGGTTCCGGCCTGGGCCCGCGTTATGTTGATTACGTTCTGGGTATCCTCAAAGCTTACTCCACTCGTGTGGGTGCAGGTCCGTTCCCGACCGAACTGTTTGATGAAACTGGCGAGTTCCTCTGCAAGCAGGGTAACGAATTCGGCGCAACTACGGGTCGTCGTCGTCGTACCGGCTGGCTGGACACCGTTGCCGTTCGTCGTGCGGTACAGCTGAACTCTCTGTCTGGCTTCTGCCTGACTAAACTGGACGTTCTGGATGGCCTGAAAGAGGTTAAACTCTGGGCT

>seq10[organism=Giant Panda Escherichia coli] Giant Panda Escherichia coli strain GP065, adenylosuccinate synthase gene.

GGGGTTGCTGTCTCCGGCCGCGCTGATGAAAGAGATGAAAGAACTGGAAGACCGTGGCATCCCCGTTCGTGAGCGTCTGCTGCTGTCTGAAGCATGTCCGCTGATCCTTGATTATCACGTTGCGCTGGATAACGCGCGTGAGAAAGCGCGTGGCGCGAAAGCGATCGGCACCACCGGTCGTGGTATCGGGCCTGCTTATGAAGATAAAGTAGCACGTCGCGGTCTGCGTGTTGGCGACCTTTTCGACAAAGAAACCTTCGCTGAAAAACTGAAAGAAGTGATGGAATATCACAACTTCCAGTTGGTTAACTACTACAAAGCTGAAGCGGTTGATTACCAGAAAGTTCTGGATGATACGATGGCTGTTGCCGACATCCTGACTTCTATGGTGGTTGACGTTTCTGACCTGCTCGACCAGGCGCGTCAGCGTGGCGATTTCGTCATGTTTGAAGGTGCGCAGGGTACGCTGCTGGATATCGACCACGGTACTTATCCGTACGTAACTTCTTCCAACACCACTGCTGGTGGCGTGGCGACCGGTTCCGGCCTGGGCCCGCGTTATGTTGATTACGTTCTGGGTATCCTCAAAGCTTACTCCACTCGTGTAGGTGCAGGTCCGTTCCCGACCGAACTGTTTGATGAAACTGGCGAGTTCCTCTGCAAGCAGGGTAACGAATTCGGCGCAACTACGGGGCGTCGTCGTCGTACCGGCTGGCTGGACACCGTTGCCGTTCGTCGTGCGGTACAGCTGAACTCCCTGTCTGGCTTCTGCCTGACTAAACTGGACGTTCTGGATGGCCTGAAAGAGGTTAAACTCTGCTTC

>seq11[organism=Giant Panda Escherichia coli] Giant Panda Escherichia coli strain GP095, adenylosuccinate synthase gene.

GGGGTTGCTGTCTCCGGCCGCGCTGATGAAAGAGATGAAAGAACTGGAAGACCGTGGCATCCCCGTTCGTGAGCGTCTGCTGCTGTCTGAAGCATGTCCGCTGATCCTTGATTATCACGTTGCGCTGGATAACGCGCGTGAGAAAGCGCGTGGCGCGAAAGCGATCGGCACTACCGGTCGTGGTATCGGGCCTGCTTATGAAGATAAAGTGGCACGTCGCGGTCTGCGTGTTGGCGACCTTTTCGACAAAAAAACCTTCGCTGAAAAACTGAAAGAAGTGATGGAATATCACAACTTCCAGTTGGTTAACTACTACAAAGCTGAAGCGGTTGATTACCAGAAAGTTCTGGATGATACGATGGCTGTTGCCGACATCCTGACTTCTATGGTGGTTGACGTTTCTGATCTGCTCGACCAGGCGCGTCAGCGTGGCGATTTCGTCATGTTTGAAGGTGCGCAGGGTACGCTGCTGGATATCGACCACGGTACTTATCCGTACGTAACTTCTTCCAACACCACTGCAGGTGGCGTGGCGACCGGTTCCGGCCTGGGCCCGCGTTATGTTGATTACGTTCTGGGTATCCTCAAAGCTTACTCCACTCGTGTGGGTGCAGGTCCATTCCCGACTGAACTGTTTGATGAAACTGGCGAGTTCCTCTGCAAGCAGGGTAACGAATTCGGCGCAACTACGGGTCGTCGTCGTCGTACCGGCTGGCTGGACACCGTTGCCGTTCGTCGTGCGGTACAGCTGAACTCCCTGTCTGGCTTCTGCCTGACTAAACTGGACGTTCTGGATGGCCTGAAAGAGGTGAAACTCTGTTTTT

>seq12[organism=Giant Panda Escherichia coli] Giant Panda Escherichia coli strain GP101, adenylosuccinate synthase gene.

GGGGTTGCTGTCTCCGGCCGCGCTGATGAAAGAGATGAAAGAACTGGAAGACCGTGGCATCCCCGTTCGTGAGCGTCTGCTGCTGTCTGAAGCATGTCCGCTGATCCTTGATTATCACGTTGCGCTGGATAACGCGCGTGAGAAAGCGCGTGGCGCGAAAGCGATCGGCACCACCGGTCGTGGTATCGGGCCTGCTTATGAAGATAAAGTAGCACGTCGCGGTCTGCGTGTTGGCGACCTTTTCGACAAAGAAACCTTCGCTGAAAAACTGAAAGAAGTGATGGAATATCACAACTTCCAGTTGGTTAACTACTACAAAGCTGAAGCGGTTGATTACCAGAAAGTTCTGGATGATACGATGGCTGTTGCCGACATCCTGACTTCTATGGTGGTTGACGTTTCTGACCTGCTCGACCAGGCGCGTCAGCGTGGCGATTTCGTCATGTTTGAAGGTGCGCAGGGTACGCTGCTGGATATCGACCACGGTACTTATCCGTACGTAACTTCTTCCAACACCACTGCTGGTGGCGTGGCGACCGGTTCCGGCCTGGGCCCGCGTTATGTTGATTACGTTCTGGGTATCCTCAAAGCTTACTCCACTCGTGTAGGTGCAGGTCCGTTCCCGACCGAACTGTTTGATGAAACTGGCGAGTTCCTCTGCAAGCAGGGTAACGAATTCGGCGCAACTACGGGGCGTCGTCGTCGTACCGGCTGGCTGGACACCGTTGCCGTTCGTCGTGCGGTACAGCTGAACTCCCTGTCTGGCTTCTGCCTGACTAAACTGGACGTTCTGGATGGCCTGAAAGAGGTTAAACTCTGG
