# Supplementary material for: Antibiotic-resistant characteristics and horizontal gene transfer ability analysis of extended-spectrum β-lactamase-producing Escherichia coli isolated from giant pandas
Source: Front Vet Sci. 2024 Jul 26;11:1394814. doi: 10.3389/fvets.2024.1394814 (PMC11310934; doi:10.3389/fvets.2024.1394814)
Supplement: Supplementary file 7 [file Data_Sheet_7.docx]

>seq1[organism=Giant Panda Escherichia coli] Giant Panda Escherichia coli strain GP001, recombinase RecA gene.

TACCTGACCGATCTTCTCACCTTTGTAGCTGTACCACGCGCCTGCTTTCTCGATAAGCTTCTCTTTCACGCCCAGGTCAACCAGCTCGCCGTAGAAGTTGATACCTTCGCCGTAGAGGATCTGGAATTCAGCCTGTTTAAACGGCGCAGCGATTTTGTTCTTCACCACTTTCACGCGGGTTTCGCTACCCACCACGTTTTCGCCCTCTTTCACCGCGCCGATACGACGGATGTCGAGACGAACAGAGGCGTAGAATTTCAGCGCGTTACCACCGGTAGTGGTTTCCGGGTTACCAAACATCACACCAATTTTCATACGGATCTGGTTGATGAAGATCAGCAGCGTGTTGGACTGCTTCAGGTTACCCGCCAGCTTACGCATCGCCTGGCTCATCATACGTGCCGCAAGGCCCATGTGAGAGTCGCCGATTTCGCCTTCGATTTCCGCTTTCGGCGTCAGTGCCGCCACGGAGTCAACGACGATAACGTCTACTGCGCCAGAACGTGCCAGGGCGTCACAGATTTCCAGTGCCTGCTCGCCGGTATCCGGCTGGGAGCACAGCAGGTTGTCGATATCGACGCCCAGTTTACGTGCGTAGATTGGGTCCAGCGCGTGTTCAGCATCGATAAACGCACAGGTTTTACCTTCGCGCTGCGCTGCGGCGATCACCTGCAACGTCAGCGTGGTTTTACCGGAAGATTCCGGTCCGGA

>seq2[organism=Giant Panda Escherichia coli] Giant Panda Escherichia coli strain GP003, recombinase RecA gene.

TACCTGACCGATCTTCTCACCTTTGTAGCTGTACCACGCGCCTGCTTTCTCGATCAGCTTCTCTTTTACGCCCAGGTCAACCAGTTCGCCGTAGAAGTTGATACCTTCGCCGTAGAGGATCTGGAATTCAGCCTGTTTAAACGGCGCAGCGATTTTGTTCTTCACCACTTTCACGCGGGTTTCGCTACCCACCACGTTTTCGCCCTCTTTCACCGCGCCGATACGACGGATGTCGAGACGAACAGAGGCGTAGAATTTCAGCGCGTTACCACCGGTAGTGGTTTCCGGGTTACCGAACATCACACCAATTTTCATACGGATCTGGTTGATGAAGATCAGCAGCGTGTTGGACTGCTTCAGGTTACCCGCCAGCTTACGCATCGCCTGGCTCATCATACGTGCCGCAAGGCCCATGTGAGAGTCGCCGATTTCGCCTTCGATTTCCGCTTTCGGCGTCAGTGCCGCCACGGAGTCAACGACGATAACGTCTACTGCGCCAGAACGCGCCAGGGCGTCACAGATTTCCAGTGCCTGCTCGCCGGTGTCCGGCTGGGAGCACAGCAGGTTGTCGATATCGACGCCCAGTTTACGTGCGTAGATTGGGTCCAGCGCGTGTTCAGCATCGATAAACGCACAGGTTTTACCTTCACGCTGCGCTGCGGCGATCACCTGCAGCGTCAGCGTGGTTTTACCGGAAGATTCCGGTCCG

>seq3[organism=Giant Panda Escherichia coli] Giant Panda Escherichia coli strain GP004, recombinase RecA gene.

CTTACCTGACCGATCTTCTCACCTTTGTAGCTGTACCACGCGCCTGCTTTCTCGATCAGCTTCTCTTTTACGCCCAGGTCAACCAGTTCGCCGTAGAAGTTGATACCTTCGCCGTAGAGGATCTGGAATTCAGCCTGTTTAAACGGCGCAGCGATTTTGTTCTTCACCACTTTCACGCGGGTTTCGCTACCCACCACGTTTTCGCCCTCTTTCACCGCGCCGATACGACGGATGTCGAGACGAACAGAGGCGTAGAATTTCAGCGCGTTACCACCGGTAGTGGTTTCCGGGTTACCGAACATCACACCAATTTTCATACGGATCTGGTTGATGAAGATCAGCAGCGTGTTGGACTGCTTCAGGTTACCCGCCAGCTTACGCATCGCCTGGCTCATCATACGTGCCGCAAGGCCCATGTGAGAGTCGCCGATTTCGCCTTCGATTTCCGCTTTCGGCGTCAGTGCCGCCACGGAGTCAACGACGATAACGTCTACTGCGCCAGAACGCGCCAGGGCGTCACAGATTTCCAGTGCCTGCTCGCCGGTGTCCGGCTGGGAGCACAGCAGGTTGTCGATATCGACGCCCAGTTTACGTGCGTAGATTGGGTCCAGCGCGTGTTCAGCATCGATAAACGCACAGGTTTTACCTTCACGCTGCGCTGCGGCGATCACCTGCAGCGTCAGCGTGGTTTTACCGGAAGATTCCGGTCCGAG

>seq4[organism=Giant Panda Escherichia coli] Giant Panda Escherichia coli strain GP012, recombinase RecA gene.

CCCTGACCGATCTTCTCACCTTTGTAGCTGTACCACGCGCCTGCTTTCTCGATAAGCTTCTCTTTCACGCCCAGGTCAACCAGCTCGCCGTAGAAGTTGATACCTTCGCCGTAGAGGATCTGGAATTCAGCCTGTTTAAACGGCGCAGCGATTTTGTTCTTCACCACTTTCACGCGGGTTTCGCTACCCACCACGTTTTCGCCCTCTTTCACCGCGCCGATACGACGGATGTCGAGACGAACAGAGGCGTAGAATTTCAGCGCGTTACCACCAGTAGTGGTTTCCGGGTTACCGAACATCACACCAATTTTCATACGGATCTGGTTGATGAAGATCAGCAGCGTGTTGGACTGCTTCAGGTTACCCGCCAGCTTACGCATCGCCTGGCTCATCATACGTGCCGCAAGGCCCATGTGAGAGTCGCCGATTTCACCTTCGATTTCCGCTTTCGGCGTCAGTGCCGCCACGGAGTCAACGACGATAACGTCTACTGCGCCAGAACGTGCCAGGGCATCACAGATTTCCAGTGCCTGCTCGCCAGTGTCCGGCTGGGAGCACAGCAGGTTGTCGATATCGACGCCCAGTTTACGTGCGTAGATTGGGTCCAGCGCGTGTTCAGCATCGATAAACGCACAGGTTTTACCTTCGCGCTGCGCTGCGGCGATCACCTGCAACGTCAGCGTGGTTTTACCGGAAGATTCCGGTCCGATA

>seq5[organism=Giant Panda Escherichia coli] Giant Panda Escherichia coli strain GP014, recombinase RecA gene.

TTCCTGACCGATCTTCTCACCTTTGTAGCTGTACCACGCGCCTGCTTTCTCGATCAGCTTCTCTTTTACGCCCAGGTCAACCAGTTCGCCGTAGAAGTTGATACCTTCGCCGTAGAGGATCTGGAATTCAGCCTGTTTAAACGGCGCAGCGATTTTGTTCTTCACCACTTTCACGCGGGTTTCGCTACCCACCACGTTTTCGCCCTCTTTCACCGCGCCGATACGACGGATGTCGAGACGAACAGAGGCGTAGAATTTCAGCGCGTTACCACCGGTAGTGGTTTCCGGGTTACCGAACATCACACCAATTTTCATACGGATCTGGTTGATGAAGATCAGCAGCGTGTTGGACTGCTTCAGGTTACCCGCCAGCTTACGCATCGCCTGGCTCATCATACGTGCCGCAAGGCCCATGTGAGAGTCGCCGATTTCGCCTTCGATTTCCGCTTTCGGCGTCAGTGCCGCCACGGAGTCAACGACGATAACGTCTACTGCGCCAGAACGCGCCAGGGCGTCACAGATTTCCAGTGCCTGCTCGCCGGTGTCCGGCTGGGAGCACAGCAGGTTGTCGATATCGACGCCCAGTTTACGTGCGTAGATTGGGTCCAGCGCGTGTTCAGCATCGATAAACGCACAGGTTTTACCTTCACGCTGCGCTGCGGCGATCACCTGCAGCGTCAGCGTGGTTTTACCGGAAGATTCCGGTCCGGA

>seq6[organism=Giant Panda Escherichia coli] Giant Panda Escherichia coli strain GP022, recombinase RecA gene.

TTCCCTGACCGATCTTCTCACCTTTGTAGCTGTACCACGCGCCTGCTTTCTCGATCAGCTTCTCTTTCACGCCCAGGTCAACCAGCTCGCCGTAGAAGTTGATACCTTCGCCGTAGAGGATCTGGAATTCAGCCTGTTTAAACGGCGCAGCGATTTTGTTCTTCACCACTTTCACGCGGGTTTCGCTACCCACCACGTTTTCGCCCTCTTTCACCGCGCCGATACGACGGATGTCGAGACGAACAGAGGCGTAGAATTTCAGCGCGTTACCACCGGTAGTGGTTTCCGGGTTACCGAACATCACACCAATTTTCATACGGATCTGGTTGATGAAGATCAGCAGCGTGTTGGACTGCTTCAGGTTACCCGCCAGCTTACGCATCGCCTGGCTCATCATACGTGCCGCAAGGCCCATGTGAGAGTCGCCGATTTCGCCTTCGATTTCCGCTTTCGGCGTCAGTGCCGCCACGGAGTCAACGACGATAACGTCTACTGCGCCAGAACGCGCCAGGGCGTCACAGATTTCCAGTGCCTGCTCGCCGGTGTCCGGCTGGGAGCACAGCAGGTTGTCGATATCGACGCCCAGTTTACGTGCGTAGATTGGGTCCAGCGCGTGTTCAGCATCGATAAACGCACAAGTTTTACCTTCGCGCTGGGCTGCGGCGATCACCTGCAACGTCAGCGTGGTTTTACCGGAAGATTCCGGTCCG

>seq7[organism=Giant Panda Escherichia coli] Giant Panda Escherichia coli strain GP030, recombinase RecA gene.

CACCTGACCGATCTTCTCACCTTTGTAGCTGTACCACGCGCCTGCTTTCTCGATCAGCTTCTCTTTTACGCCCAGGTCAACCAGTTCGCCGTAGAAGTTGATACCTTCGCCGTAGAGGATCTGGAATTCAGCCTGTTTAAACGGCGCAGCGATTTTGTTCTTCACCACTTTCACGCGGGTTTCGCTACCCACCACGTTTTCGCCCTCTTTCACCGCGCCGATACGACGGATGTCGAGACGAACAGAGGCGTAGAATTTCAGCGCGTTACCACCGGTAGTGGTTTCCGGGTTACCGAACATCACACCAATTTTCATACGGATCTGGTTGATGAAGATCAGCAGCGTGTTGGACTGCTTCAGGTTACCCGCCAGCTTACGCATCGCCTGGCTCATCATACGTGCCGCAAGGCCCATGTGAGAGTCGCCGATTTCGCCTTCGATTTCCGCTTTCGGCGTCAGTGCCGCCACGGAGTCAACGACGATAACGTCTACTGCGCCAGAACGCGCCAGGGCGTCACAGATTTCCAGTGCCTGCTCGCCGGTGTCCGGCTGGGAGCACAGCAGGTTGTCGATATCGACGCCCAGTTTACGTGCGTAGATTGGGTCCAGCGCGTGTTCAGCATCGATAAACGCACAGGTTTTACCTTCACGCTGCGCTGCGGCGATCACCTGCAGCGTCAGCGTGGTTTTACCGGAAGATTCCGGTCCG

>seq8[organism=Giant Panda Escherichia coli] Giant Panda Escherichia coli strain GP032, recombinase RecA gene.

TCCCTGACCGATCTTCTCACCTTTGTAGCTGTACCATGCGCCTGCTTTCTCGATCAGCTTCTCTTTCACGCCCAGATCAACCAGTTCGCCATAGAAGTTGATACCTTCGCCGTAGAGGATCTGGAATTCAGCCTGTTTAAACGGCGCAGCGATTTTGTTCTTCACCACTTTCACACGGGTTTCGCTACCCACCACGTTTTCGCCCTCTTTCACCGCGCCGATACGACGGATGTCGAGACGAACAGAGGCGTAGAATTTCAGCGCGTTACCACCGGTAGTGGTTTCCGGGTTACCGAACATCACACCAATTTTCATACGGATCTGGTTGATGAAGATCAGCAGCGTGTTGGACTGCTTCAGGTTACCCGCCAGCTTACGCATCGCCTGGCTCATCATACGTGCCGCAAGGCCCATGTGAGAGTCGCCGATTTCGCCTTCGATTTCCGCTTTCGGCGTCAGTGCCGCCACGGAGTCAACGACGATAACGTCTACTGCGCCAGAACGTGCCAGGGCGTCACAGATTTCCAGTGCCTGCTCGCCAGTGTCCGGCTGGGAGCACAGCAGGTTGTCGATATCGACGCCCAGTTTACGTGCGTAGATTGGGTCCAGCGCGTGTTCAGCATCGATAAACGCACAAGTTTTACCTTCGCGCTGGGCTGCGGCGATCACCTGCAACGTCAGCGTGGTTTTACCGGAAGATTCCGGTCCGGATT

>seq9[organism=Giant Panda Escherichia coli] Giant Panda Escherichia coli strain GP050, recombinase RecA gene.

CCTGACCGATCTTCTCACCTTTGTAGCTGTACCACGCGCCTGCTTTCTCGATAAGCTTCTCTTTCACGCCCAGGTCAACCAGCTCGCCGTAGAAGTTGATACCTTCGCCGTAGAGGATCTGGAATTCAGCCTGTTTAAACGGCGCAGCGATTTTGTTCTTCACCACTTTCACGCGGGTTTCGCTACCCACCACGTTTTCGCCCTCTTTCACCGCGCCGATACGACGGATGTCGAGACGAACAGAGGCGTAGAATTTCAGCGCGTTACCACCGGTAGTGGTTTCCGGGTTACCGAACATCACACCAATTTTCATACGGATCTGGTTGATGAAGATCAGCAGCGTGTTGGACTGCTTCAGGTTACCCGCCAGCTTACGCATCGCCTGGCTCATCATACGTGCCGCAAGGCCCATGTGAGAGTCGCCGATTTCGCCTTCGATTTCCGCTTTCGGCGTCAGTGCCGCCACGGAGTCAACGACGATAACGTCTACTGCGCCAGAACGTGCCAGGGCATCACAGATTTCCAGTGCCTGCTCGCCGGTGTCCGGCTGGGAGCACAGCAGGTTGTCGATATCGACGCCCAGTTTACGTGCGTAGATTGGGTCCAGCGCGTGTTCAGCATCGATAAACGCACAAGTTTTACCTTCGCGCTGGGCTGCGGCGATCACCTGCAACGTCAGCGTGGTTTTACCGGAAGATTCCGGCCGGA

>seq10[organism=Giant Panda Escherichia coli] Giant Panda Escherichia coli strain GP065, recombinase RecA gene.

CTACCTGACCGATCTTCTCACCTTTGTAGCTGTACCACGCGCCTGCTTTCTCGATCAGCTTCTCTTTTACGCCCAGGTCAACCAGTTCGCCGTAGAAGTTGATACCTTCGCCGTAGAGGATCTGGAATTCAGCCTGTTTAAACGGCGCAGCGATTTTGTTCTTCACCACTTTCACGCGGGTTTCGCTACCCACCACGTTTTCGCCCTCTTTCACCGCGCCGATACGACGGATGTCGAGACGAACAGAGGCGTAGAATTTCAGCGCGTTACCACCGGTAGTGGTTTCCGGGTTACCGAACATCACACCAATTTTCATACGGATCTGGTTGATGAAGATCAGCAGCGTGTTGGACTGCTTCAGGTTACCCGCCAGCTTACGCATCGCCTGGCTCATCATACGTGCCGCAAGGCCCATGTGAGAGTCGCCGATTTCGCCTTCGATTTCCGCTTTCGGCGTCAGTGCCGCCACGGAGTCAACGACGATAACGTCTACTGCGCCAGAACGCGCCAGGGCGTCACAGATTTCCAGTGCCTGCTCGCCGGTGTCCGGCTGGGAGCACAGCAGGTTGTCGATATCGACGCCCAGTTTACGTGCGTAGATTGGGTCCAGCGCGTGTTCAGCATCGATAAACGCACAGGTTTTACCTTCACGCTGCGCTGCGGCGATCACCTGCAGCGTCAGCGTGGTTTTACCGGAAGATTCCGGTCCGA

>seq11[organism=Giant Panda Escherichia coli] Giant Panda Escherichia coli strain GP095, recombinase RecA gene.

TTCCTGACCGATCTTCTCACCTTTGTAGCTGTACCACGCGCCTGCTTTCTCGATCAGCTTCTCTTTCACGCCCAGGTCAACCAGCTCGCCGTAGAAGTTGATACCTTCGCCGTAGAGGATCTGGAATTCAGCCTGTTTAAACGGCGCAGCGATTTTGTTCTTCACCACTTTCACGCGGGTTTCGCTACCCACCACGTTTTCGCCCTCTTTCACCGCGCCGATACGACGGATGTCGAGACGAACAGAGGCGTAGAATTTCAGCGCGTTACCACCGGTAGTGGTTTCCGGGTTACCGAACATCACACCAATTTTCATACGGATCTGGTTGATGAAGATCAGCAGCGTGTTGGACTGCTTCAGGTTACCCGCCAGCTTACGCATCGCCTGGCTCATCATACGTGCCGCAAGGCCCATGTGAGAGTCGCCGATTTCGCCTTCGATTTCCGCTTTCGGCGTCAGTGCCGCCACGGAGTCAACGACGATAACGTCTACTGCGCCAGAACGCGCCAGGGCGTCACAGATTTCCAGTGCCTGCTCGCCGGTGTCCGGCTGGGAGCACAGCAGGTTGTCGATATCGACGCCCAGTTTACGTGCGTAGATTGGGTCCAGCGCGTGTTCAGCATCGATAAACGCACAAGTTTTACCTTCGCGCTGGGCTGCGGCGATCACCTGCAACGTCAGCGTGGTTTTACCGGAAGATTCCGGTCCGAATTT

>seq12[organism=Giant Panda Escherichia coli] Giant Panda Escherichia coli strain GP101, recombinase RecA gene.

CCTGACCGATCTTCTCACCTTTGTAGCTGTACCACGCGCCTGCTTTCTCGATCAGCTTCTCTTTTACGCCCAGGTCAACCAGTTCGCCGTAGAAGTTGATACCTTCGCCGTAGAGGATCTGGAATTCAGCCTGTTTAAACGGCGCAGCGATTTTGTTCTTCACCACTTTCACGCGGGTTTCGCTACCCACCACGTTTTCGCCCTCTTTCACCGCGCCGATACGACGGATGTCGAGACGAACAGAGGCGTAGAATTTCAGCGCGTTACCACCGGTAGTGGTTTCCGGGTTACCGAACATCACACCAATTTTCATACGGATCTGGTTGATGAAGATCAGCAGCGTGTTGGACTGCTTCAGGTTACCCGCCAGCTTACGCATCGCCTGGCTCATCATACGTGCCGCAAGGCCCATGTGAGAGTCGCCGATTTCGCCTTCGATTTCCGCTTTCGGCGTCAGTGCCGCCACGGAGTCAACGACGATAACGTCTACTGCGCCAGAACGCGCCAGGGCGTCACAGATTTCCAGTGCCTGCTCGCCGGTGTCCGGCTGGGAGCACAGCAGGTTATCGATATCGACGCCCAGTTTACGTGCGTAGATTGGGTCCAGCGCGTGTTCAGCATCGATAAACGCACAGGTTTTACCTTCACGCTGCGCTGCGGCGATCACCTGCAGCGTCAGCGTGGTTTTACCGGAAGATTCCGGCCGAA
